# Periostin as a novel biomarker for postoperative recurrence of chronic rhinosinitis with nasal polyps

**DOI:** 10.1038/s41598-018-29612-2

**Published:** 2018-07-30

**Authors:** Takahiro Ninomiya, Emiko Noguchi, Takenori Haruna, Masayo Hasegawa, Takuto Yoshida, Yukiko Yamashita, Mitsuhiro Okano, Naohiro Yoshida, Shinichi Haruna, Yasunori Sakuma, Shoichiro Ohta, Junya Ono, Kenji Izuhara, Masafumi Okada, Masanori Kidoguchi, Takahiro Tokunaga, Masayuki Okamoto, Masafumi Kanno, Masafumi Sakashita, Tetsuji Takabayashi, Norihiko Narita, Shigeharu Fujieda

**Affiliations:** 1 Departments of Otorhinolaryngology Head & Neck Surgery, Faculty of Medical Sciences, University of Fukui, Fukui, Japan; 2 Department of Medical Genetics, Faculty of Medicine, University of Tsukuba, Tsukuba, Japan; 3 Department of Otolaryngology-Head and Neck Surgery, Okayama University Graduate School of Medicine, Dentistry and Pharmaceutical Sciences, Okayama, Japan; 4 Department of Otolaryngology, Jichi Medical University, Saitama Medical Center, Saitama, Japan; 5 Department of Otorhinolaryngology Head & Neck Surgery, Dokkyo Medical University, Tochigi, Japan; 6 Department of Otorhinolaryngology, Yokohama City University Medical Center, Kanagawa, Japan; 7 Department of Otorhinolaryngology, International University of Health and Welfare School of Medicine, Narita, Japan; 8 Division of Medical Biochemistry, Department of Biomolecular Sciences, Saga Medical School, Saga, Japan; 9 Department of Laboratory Medicine, Department of Biomolecular Sciences, Saga Medical School, Saga, Japan; 10 Shino-test Co. Ltd., Sagamihara, Japan; 11 University Hospital Medical Information Network Research Center, University of Tokyo, Tokyo, Japan

## Abstract

We previously reported that chronic rhinosinusitis with nasal polyps (CRSwNP) was subdivided into four chronic rhinosinusitis (CRS) subtypes using the JESREC scoring system. We sought to identify the gene expression profile and biomarkers related with CRSwNP by RNA-sequence. RNA-sequencing was performed to identify differentially expressed genes between nasal polyps (NPs) and inferior turbinate mucosa from 6 patients with CRSwNP, and subsequently, quantitative real-time PCR was performed to verify the results. ELISA was performed to identify possible biomarkers for postoperative recurrence. In the RNA-sequencing results, *periostin* (*POSTN*) expression was the highest in NP. We focused on *POSTN* and investigated the protein level of POSTN by immunohistochemistry and ELISA. POSTN was diffusely expressed in moderate and severe eosinophilic CRS using immunohistochemistry, and its staining pattern was associated with the severity of the phenotype of the CRSwNP (*P* < 0.05). There was a significant difference between the POSTN high/low groups for postoperative recurrence when the cutoff point was set at 115.5 ng/ml (*P* = 0.0072). Our data suggests that the protein expression level of POSTN was associated with the severity of CRSwNP, and serum POSTN can be a novel biomarker for postoperative recurrence of CRSwNP.

## Introduction

Chronic rhinosinusitis (CRS) is one of the most common diseases worldwide. The prevalence of CRS is 10–14% in Europe and the US, and the economic burden was estimated at approximately 60 billion US dollars in 2011^1,2^. In East Asia, the prevalence of CRS is 5–10%, almost equivalent with Europe and the US^3^. In the guidelines of a European position paper on rhinosinusitis and nasal polyps in 2012 (EPOS 2012), rhinosinusitis is diagnosed based on symptoms, physical examination and radiographic findings: nasal obstruction, nasal discharge, endoscopic signs of nasal polyps (NPs) and mucopurulent discharge, and CT changes^2^. CRS is defined as the condition with the symptoms and examination findings lasting for more than 12 weeks^2^. CRS is categorized by being positive or negative of NPs; chronic rhinosinusitis with NPs (CRSwNP) and chronic rhinosinusitis without NPs (CRSsNP)^2^. In Western countries, most of the CRSwNP belong to eosinophilic CRS (ECRS), which is defined as >5 or >10 eosinophils per high-power field (HPF), and has been categorized to eosinophilic dominant inflammation^4,5^. Eosinophilic inflammation in CRS is considered to be reflected by the severity and poor outcome with treatment^6,7^. In contrast, in East Asia, CRSwNP has been reported to be neutrophil dominant inflammation^6^. Recently, however, we reported that the proportion of the eosinophil dominant type of CRSwNP in Japan was nearly equal to that in the Western countries^7^. We also reported that CRSwNP was subdivided into four subtypes; non-ECRS, mild ECRS, moderate ECRS and severe ECRS by using the scoring system based on one/both sides of disease, presence of NPs, ethmoid dominant CT shadow, and eosinophil ratio in peripheral blood (Japanese Epidemiological Survey of Refractory Eosinophilic Chronic Rhinosinusitis Study: JESREC Study)^7^.

CRSwNP is considered to be related to Th2 dominant inflammation mainly with eosinophil infiltration^8^. Many genes related to eosinophilic recruitment and activation were reported to be upregulated in CRSwNP (e.g., *C-C motif chemokine ligand 18* (*CCL18*), *CCL23* and *interleukin 32* (*IL32*)^9–11^. Epithelial cell cytokines may have an important role for eosinophil and Th2 type cell recruitment at the local site, for example *thymic stromal lymphopoietin (TSLP)* and *IL33* were essential for dendritic cell polarization and T cell differentiation^8,12–14^. Although some genes were found to be associated with mucosal inflammation and epithelial repair in CRS, the pathogenic mechanism of CRS has not been well elucidated.

Gene profiling technology such as microarray and serial analysis of gene expression (SAGE) can identify novel genes related to disease condition by comparing the global gene expression profile in normal tissues with that in diseased^15^. Liu *et al*. reported 19 upregulated genes and 8 downregulated genes in NPs compared with the tissue from the uncinate process from patients undergoing septoplasty using microarray^16^. The most upregulated gene by microarray was *osteopontin*, which is involved in the pathogenesis of inflammatory and immune responses, with osteopontin being found to play roles in promoting eosinophil migration and activation *in vitro*^16^. Li *et al*. examined expression levels of NPs and inferior turbinate (IT) obtained from CRS patients mucosa using microarray, and focused on the genes associated with regulatory T cell (T-reg) and helper T cell (Th cell)^17^. Forkhead box P3 (FOXP3) which is essential for T-reg development was upregulated in NPs compared with that in IT, and genes associated with Th2 (*GATA binding protein 3*: *GATA3*) or Th17 response (*RAR related orphan receptor C*: *RORC*) were downregulated in NPs^17^. Lee *et al*. reported the highest expression tags in NPs with SAGE were *interferon induced protein with tetratricopeptide repeats 3 (IFIT3)*, *CD74*, *Lipocalin 2 (LCN2)* and *TYRO protein tyrosine kinase binding protein (TYROBP)*^15^.

RNA sequencing (RNA-seq) is an innovative technology that uses a next-generation sequencer to sequence all transcripts including mRNAs, non-coding RNAs and small RNAs^18^. The primal purpose of RNA-seq is to measure the expression levels of each transcript, particularly to identify new genes/transcripts, under different conditions^18–20^. In contrast to microarray and SAGE, RNA-seq can detect the transcripts for a large dynamic range of expression levels with very low background signal^18,19^. Actually, RNA-seq has identified many genes that could be used as a biomarker for diseases such as cancer^19,21^ and asthma^22^.

In this study, we used RNA-seq to compare the expression profile in NPs obtained from patients with CRSwNP with that in IT mucosa from the same patients. Quantitative real time PCR (qPCR) was used to confirm the initial results obtained by RNA-seq. Then, we focused on *periostin* (*POSTN*) whose expression level was the highest in NPs, and immunohistochemical analysis was performed to evaluate the protein expression level of POSTN in NPs. The association between serum POSTN level and postoperative recurrence was also investigated to see whether serum POSTN is a possible biomarker for postoperative recurrence.

## Results

### Transcriptome analysis

**Figure 1:**
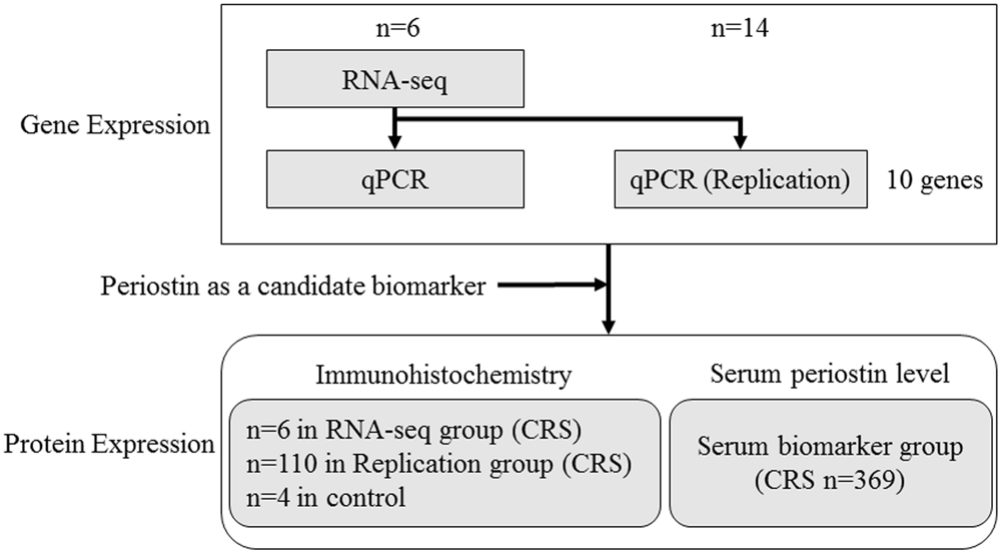
Flowchart of the present study. RNA-seq, RNA sequencing; qPCR, quantitative real-time PCR; CRS, chronic rhinosinusitis.

**Figure 2:**
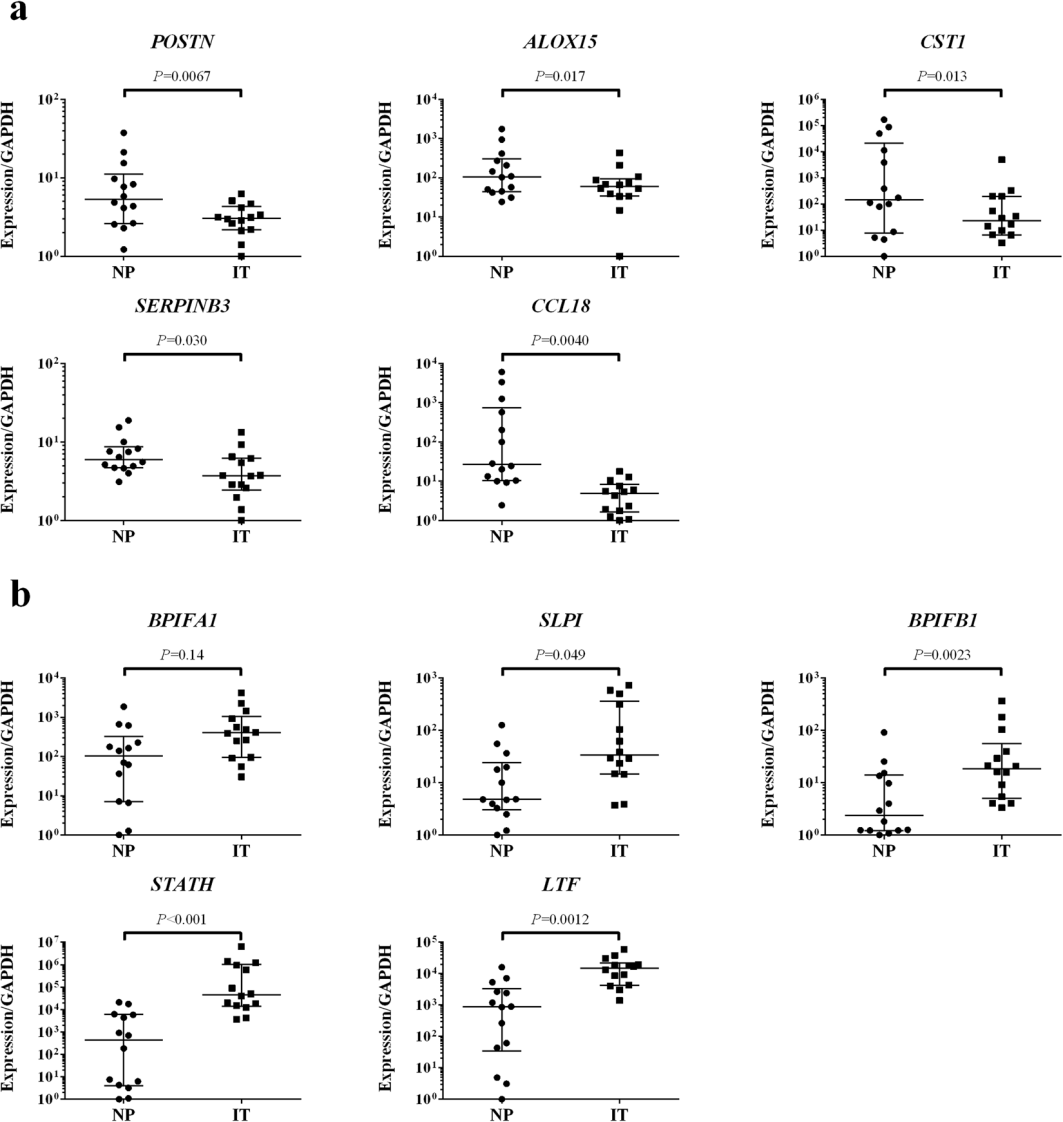
Relative expression levels of the 10 genes by qPCR analysis in the replication group (n = 14, Wilcoxon rank sum test). Bars show the median values and interquartile range. **(a)** The upregulated genes detected by RNA-seq (*POSTN, ALOX15, CST1, SERPINB3* and *CCL18*). **(b)** The downregulated genes detected by RNA-seq (*BPIFA1, SLPI, BPIFB1, STATH* and *LTF*). NP, nasal polyp; IT, inferior turbinate; *POSTN*, periostin; *ALOX15*, arachidonate 15-lipoxygenase; *CST1*, cystatin SN; *SERPINB3*, serpin peptidase inhibitor, clade B (ovalbumin), member3; *CCL18*, C-C motif chemokine ligand 18; *BPIFA1*, BPI fold containing family A member 1; *SLPI*, secretory leukocyte peptidase inhibitor; *BPIFB1*, BPI fold containing family B member 1; *STATH*, statherin; *LTF*, lactotransferrin.

Figure 1 shows the flowchart of the present study, and characteristics of study subjects are described in Table 1. Patients were subdivided according to the JESREC scoring system, as described previously, into non-ECRS, mild ECRS, moderate ECRS and severe ECRS groups^7^. We analyzed the gene expressions in 6 samples of NP with CRSwNP using RNA-seq, and compared the expression levels in NP with those in IT obtained from the same patients. When comparing the expression levels between NP and IT, 3,574 transcripts were statistically significantly different (*q* < 0.05). Of these, 1,264 transcripts were upregulated in NP with log fold changes (logFC) > 1 (Supplementary Table S1), and 899 transcripts were downregulated in NP with logFC < −1 (Supplementary Table S2). We focused on the 5 most upregulated and 5 most downregulated genes that were highly expressed in NP. These 10 genes expression levels in the RNA-seq group obtained from RNA-seq are shown in Table 2. To confirm the RNA-seq results, qPCR analysis was performed using the same samples (6 patients used in RNA-seq study), and the results of qPCR were concordant with those of RNA-seq (Supplementary Fig. S2). Furthermore, we analyzed these 10 genes expression levels in NP/IT in replication group using qPCR. We performed qPCR using independent 14 patients whose RNA were available (Replication group, Fig. 2). Although there was a gender difference (12 male vs 2 female) in the qPCR group, no statistically significant differences were observed between the expression levels of the 10 genes in males and females (*P* > 0.05). The expression levels in the replication group were similar to those in the RNA-seq group, and the expression levels of *POSTN, arachidonate 15-lipoxygenase (ALOX15), cystatin SN (CST1), serpin peptidase inhibitor, clade B (ovalbumin), member3 (SERPINB3)*, *CCL18*, *secretory leukocyte peptidase inhibitor (SLPI), BPI fold containing family B member 1 (BPIFB1), statherin (STATH)* and *lactotransferrin (LTF)* were significantly different in NP and IT samples (*P* < 0.05, Fig. 2). There was no significant difference between expression levels of the gene coding for *BPI fold containing family A member 1 (BPIFA1)* in NP and IT samples from the replication group (*P* > 0.05).

**Table 1:** Characteristics of subjects.

	RNA-seq group	Replication group (qPCR)	Replication group (immunohistochemistry)	Serum biomarker group
Characteristics				
Subject no.	6	14	110	369
Gender (M/F)	4/2	12/2	68/42	239/130
Age (mean ± SD)	56.7 ± 15.0	57.4 ± 18.3	56.1 ± 14.7	53.7 ± 14.6
Comorbid of asthma	2/6 (33.3%)	3/14 (21.4%)	31/110 (28.2%)	124/369 (33.6%)
Diagnosis				
Non-ECRS	2	6	34	122
Mild ECRS	0	2	20	64
Moderate ECRS	3	4	35	116
Severe ECRS	1	2	21	67
Experiments				
RNA-seq	6	(−)	(−)	(−)
qPCR	6	14	(−)	(−)
Immunohistochemistry	6	(−)	110	(−)
ELISA (periostin)	(−)	(−)	(−)	369

RNA-seq, RNA sequencing; qPCR, quantitative real time PCR; ECRS, eosinophilic chronic rhinosinusitis.

**Table 2:** The 5 most upregulated and 5 most downregulated genes that were highly expressed in NP.

Gene	*P* value	Adjusted *P* value (FDR)	Log fold change	NP (FPKM)	IT (FPKM)
Upregulated					
*POSTN*	4.02 × 10^−4^	3.58 × 10^−3^	3.11	7.98 × 10^2^	4.96 × 10
*ALOX15*	3.92 × 10^−3^	2.21 × 10^−2^	3.07	6.34 × 10^2^	8.02 × 10
*CST1*	4.03 × 10^−3^	2.26 × 10^−2^	4.77	6.29 × 10^2^	8.53
*SERPINB3*	8.30 × 10^−4^	6.41 × 10^−3^	2.90	5.45 × 10^2^	1.31 × 10^2^
*CCL18*	1.23 × 10^−11^	1.07 × 10^−9^	6.83	3.94 × 10^2^	9.27 × 10^−1^
Downregulated					
*BPIFA1*	1.40 × 10^−3^	9.77 × 10^−3^	−3.01	4.13 × 10^3^	2.66 × 10^4^
*SLPI*	9.77 × 10^−7^	2.63 × 10^−5^	−2.16	3.00 × 10^3^	1.11 × 10^4^
*BPIFB1*	9.02 × 10^−5^	1.12 × 10^−3^	−2.74	2.02 × 10^3^	1.18 × 10^4^
*STATH*	7.00 × 10^−13^	7.96 × 10^−11^	−10.8	9.14 × 10^2^	6.71 × 10^4^
*LTF*	1.29 × 10^−9^	7.28 × 10^−8^	−4.47	8.27 × 10^2^	6.92 × 10^3^

Log fold change indicates the logarithm base 2 log fold change of count per million (CPM) mapped reads; NP, nasal polyp; IT, inferior turbinate; NP (FPKM) and IT (FPKM) represent averages of fragments per kilobase of transcript per million mapped fragments (FPKM) in NP and IT samples, respectively; *POSTN*, periostin; *ALOX15*, arachidonate 15-lipoxygenase; *CST1*, cystatin SN; *SERPINB3*, serpin peptidase inhibitor, clade B (ovalbumin), member3; *CCL18*, C-C motif chemokine ligand 18; *BPIFA1*, BPI fold containing family A member 1; *SLPI*, secretory leukocyte peptidase inhibitor; *BPIFB1*, BPI fold containing family B member 1; *STATH*, statherin; *LTF*, lactotransferrin.

### POSTN immunohistochemical analysis

**Figure 3:**
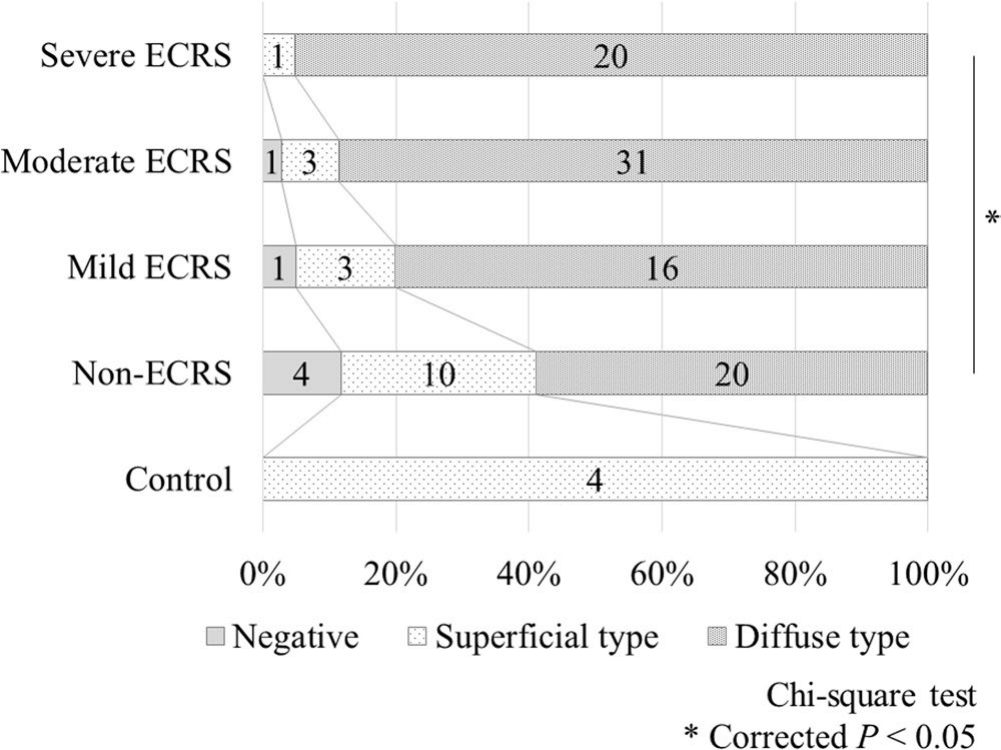
Periostin deposition patterns in each group based on the immunohistochemical analysis. Chi-square test: *corrected *P* < 0.05. ECRS, eosinophilic chronic rhinosinusitis.

We then focused on *POSTN*, whose expression was highest among the upregulated genes in the NP according to RNA-seq. To examine the association between POSTN protein expression level and CRSwNP subtype according to the JESREC scoring system, we performed immunohistochemical analysis of NP obtained from 6 patients (RNA-seq group). POSTN deposition pattern in NP was categorized based on the location of POSTN staining reported by Shiono *et al*.^23^ (Supplementary Fig. S1). POSTN staining pattern in NP tended to be more diffuse in those obtained from patients with more severe phenotypes (Supplementary Table S3). Furthermore, we performed immunohistochemical analysis using NP samples obtained independently from 110 patients with CRSwNP (Replication group) and IT samples obtained from 4 patients undergoing septoplasty as control (Fig. 3). In the control group, POSTN deposition was observed only in the subepithelial layer, and the superficial type was observed more in CRSwNP from patients with a less severe phenotype than those with a more severe phenotype, and a significant difference in POSTN staining patterns was observed between the non-ECRS and severe ECRS subtypes (corrected *P* = 0.037).

### Serum POSTN levels by ELISA

**Figure 4:**
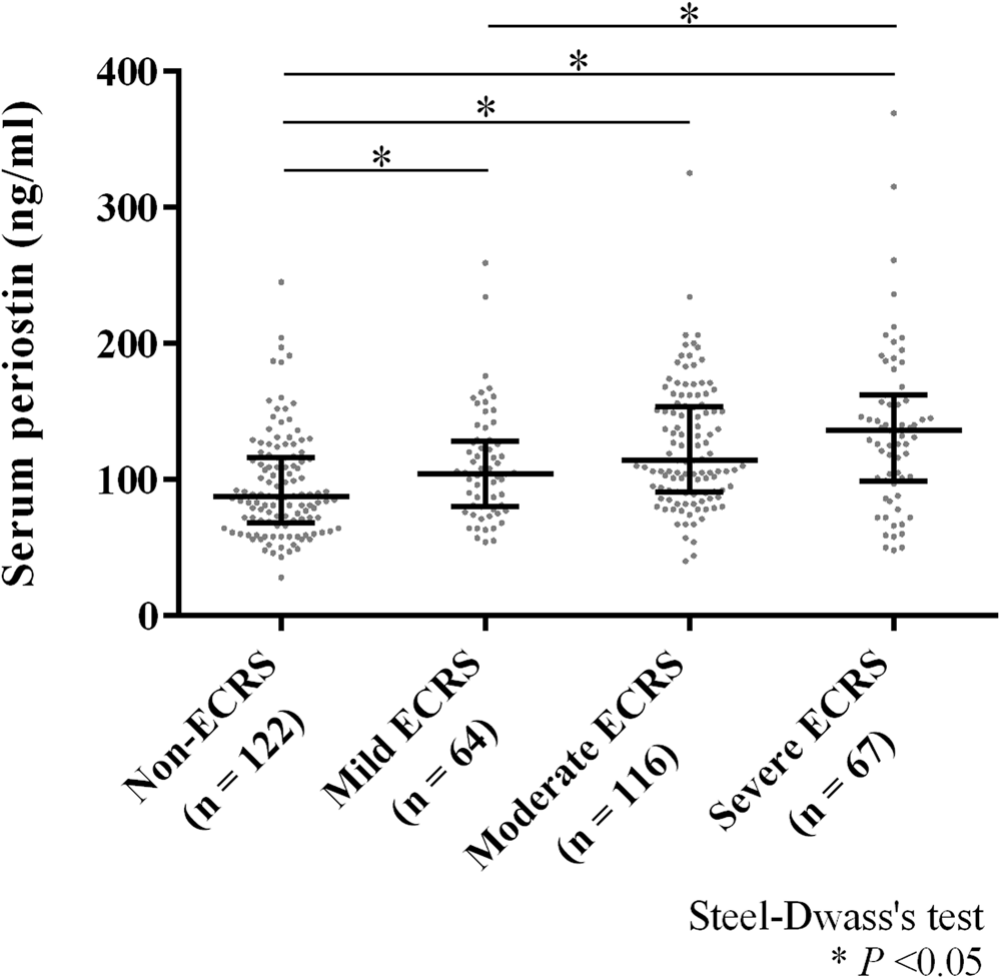
Serum periostin levels in the classified groups by the algorithm of JESREC study. Bars show the median values and interquartile range; Steel-Dwass’s test: *P < 0.05. ECRS, eosinophilic chronic rhinosinusitis.

**Figure 5:**
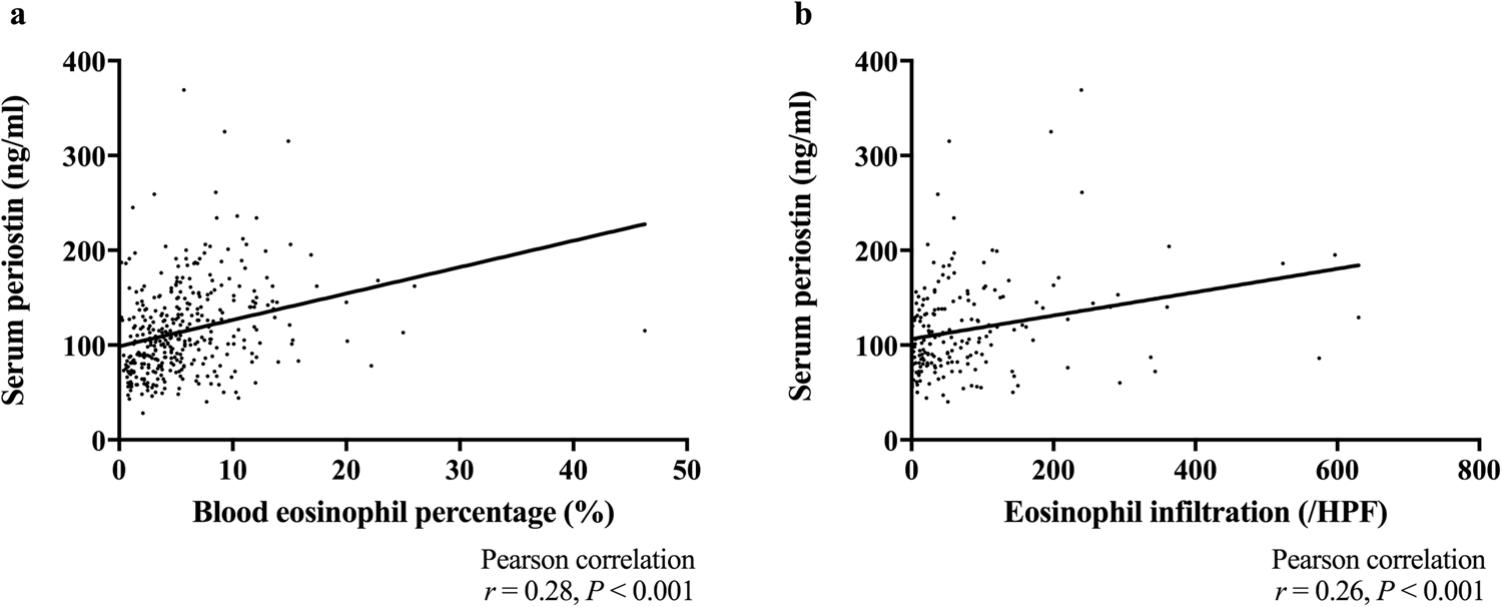
(**a**) Correlation between serum periostin level and blood eosinophil percentage. N = 369; Pearson correlation test: *r = 0.28*, P* < 0.001. (**b**) Correlation between serum periostin level and tissue eosinophil infiltration. N = 204; Pearson correlation test: *r = 0.26*, P* < 0.001. HPF, high-power field.

Next, we investigated the association between serum POSTN level and severity of CRSwNP to see whether serum POSTN is a possible biomarker for CRSwNP. First, we examined serum POSTN in each CRSwNP subtype (Fig. 4). The median (range) of serum POSTN in non-ECRS, mild ECRS, moderate ECRS and severe ECRS were 87.5 (28–245) ng/ml, 104.5 (54–259) ng/ml, 114 (40–325) ng/ml and 136 (48–369) ng/ml, respectively. Differences were statistically significant between all pairs of groups other than mild vs moderate and moderate vs severe ECRS (*P* < 0.05). Because patients with CRSwNP often have asthma, and because serum POSTN is reportedly elevated in patients with asthma, we divided the patients into CRSwNP with and without asthma groups. The medians (range) of serum POSTN in the CRSwNP with and without asthma groups were 125.5 (48–369) ng/ml and 101 (28–325) ng/ml, respectively, and serum POSTN was significantly increased in the asthma group (*P* < 0.001). Second, we analyzed the correlation between serum POSTN and blood eosinophil percentage. We found a positive correlation between serum POSTN and blood eosinophil percentage (Fig. 5a, n = 369, r = 0.28, *P* < 0.001) and a positive correlation between serum POSTN and tissue eosinophil infiltration (Fig. 5b, n = 204, r = 0.26, *P* < 0.001).

**Figure 6:**
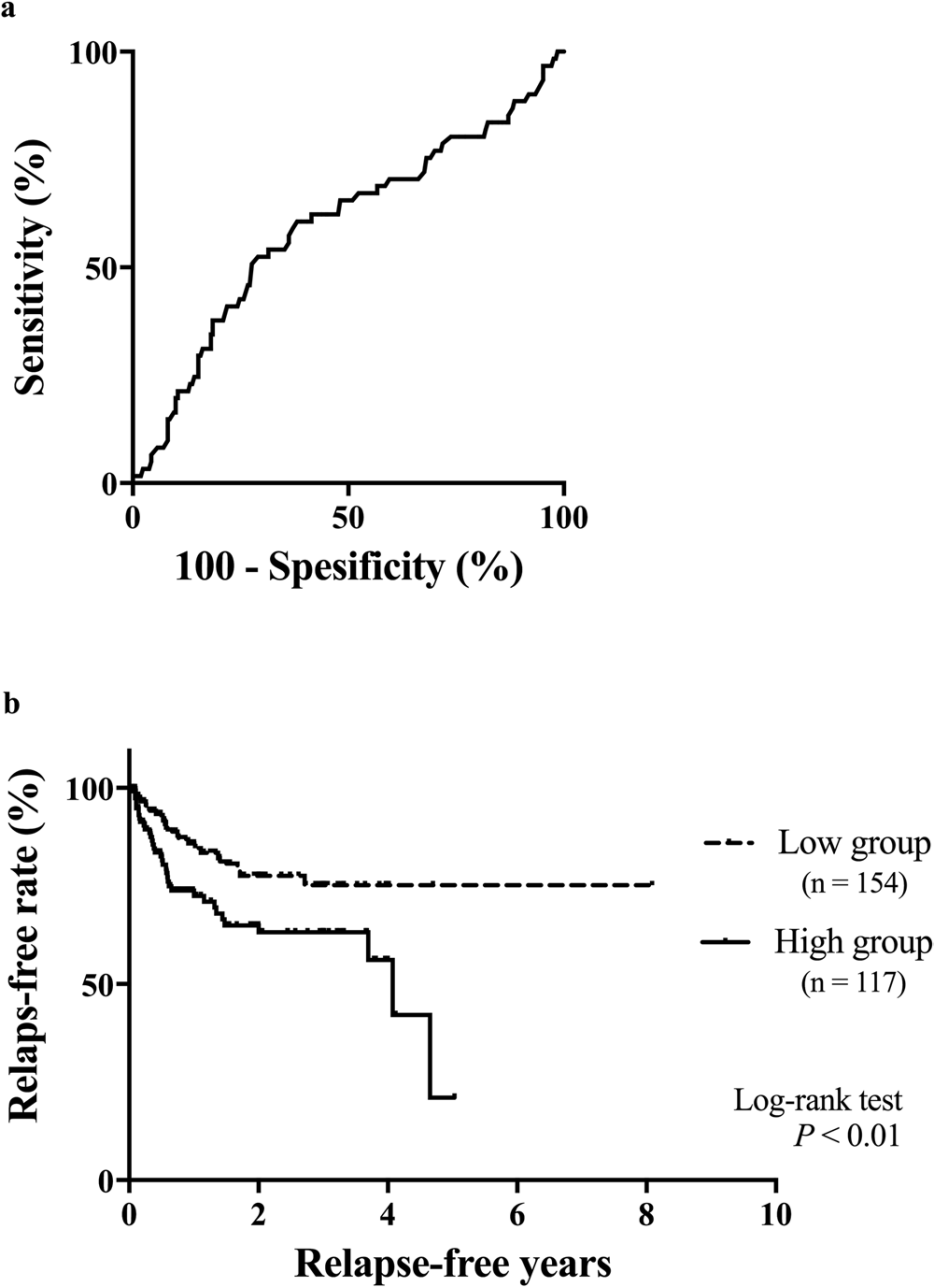
(**a**) ROC curve for postoperative recurrence to determine the cutoff point of serum periostin. (**b**) Kaplan–Meier curves of the relapse-free rate at the cutoff point of serum periostin 115.5 ng/ml; log-rank test: *P* = 0.0072. ROC, receiver operating characteristics.

Finally, we examined the association between serum POSTN and postoperative recurrence to see whether serum POSTN is a possible biomarker for postoperative recurrence. Receiver operating characteristics (ROC) curves were used to determine the cutoff point for postoperative recurrence (Fig. 6a). The area under the curve (AUC) was 0.595 (standard error: 0.044, 95% confidence interval: 0.509 to 0.681). Serum POSTN = 115.5 ng/ml was determined as the optimal cutoff point which were the closest to the top-left corner of ROC^24^ (sensitivity 60.7%, specificity 61.9%). Kaplan-Meier plot of postoperative recurrence for whole CRS showed that there was a significant difference between these two groups when the cutoff point was set at 115.5 ng/ml (Fig. 6b, *P* = 0.0072). In the analysis restricted to non-CRS, the survival curve for POSTN high group and that for POSTN low group is different, but their trends were not statistically significant (Supplementary Fig. S3, P = 0.115).

## Discussion

In the present study, we identified genes differentially expressed in NP compared with IT by using RNA-seq technologies. Of these, the expression level of *POSTN*, *ALOX15*, *CST1*, *SERPINB3*, *CCL18*, *SLPI*, *BPIFB1*, *STATH* and *LTF* were validated to be statistically significantly different by qPCR analysis using independent NP/IT samples. We focused on *POSTN* whose expression level was the highest among the upregulated genes in RNA-seq and the results were validated in the replication samples. Additionally, we showed that serum POSTN is a biomarker of postoperative CRSwNP recurrence, and its optimal cut-off point was 115.5 ng/ml.

POSTN is an extracellular matrix protein belonging to the fasciclin family^25,26^, and is produced in fibroblasts and its expression level is controlled by IL-4, IL-13, TGF-β, connective tissue growth factor (CCN2/CTGF) and bone morphogenetic protein 2 (BMP-2)^25–30^. POSTN is directly attached to collagen type I, V, fibronectin, tenascin-C, and POSTN itself and this complex assists in subepithelial fibrosis and tissue remodeling^26,30^. It has been reported that POSTN is associated with Th2/eosinophilic inflammation^31,32^. In the present study, we performed immunohistochemical analysis to determine the distribution of POSTN in NPs. We compared POSTN deposition pattern with phenotypes of CRSwNP diagnosed based on the criteria of the JESREC Study^7^. Shiono *et al*. reported that the POSTN deposition pattern was related with the number of infiltrated eosinophils in NPs^23^, which reflect the severity and poor outcome by treatment^6,7^. They also reported 2 types of POSTN deposition patterns based on the location of expression: superficial type, expressed only in the subepithelial layer; and diffuse type, expressed throughout the lamina propria starting just below the basement membrane^23^. In the present study, we found that the diffuse type of POSTN deposition was observed more in high-grade than in low-grade JESREC subtypes. High expression of POSTN has been reported in NPs of CRSwNP^23,33–35^, and POSTN expression level was positively correlated with IL-5 and IL-13, which may reflect Th2 type inflammation^35,36^. Our results emphasized the relationship between POSTN and Th2 inflammation in NPs.

Then, we investigated the association between serum POSTN level and severity of CRSwNP to see whether serum POSTN is a possible biomarker for CRSwNP. Serum POSTN was reported as a surrogate biomarker for the phenotype of Th2 high asthma^31,32,37^. Additionally, a recent study showed that serum POSTN was a biomarker of nasal polyps in patients with asthma^38^. We found that serum POSTN level was increased as patient’s severity increased, and that the significant difference was observed in serum POSTN level according to the severity of CRSwNP groups following the criteria of JESREC Study. Furthermore, serum POSTN level was positively correlated with blood eosinophil percentage and tissue eosinophil infiltration. We previously reported that the percentage of postoperative recurrence in CRSwNP was 23.1% while that of severe ECRS by JESREC criteria was 51.8%^7^. In the present study, we found that serum POSTN level can be another candidate biomarker to predict postoperative recurrence. The optimal cutoff point was determined to be 115.5 ng/ml according to the closest point to top-left in ROC curve^24^. Izuhara *et al*. reported 95 ng/ml was optimal cutoff point for asthma when comparing with healthy control^39^. Serum POSTN was reported to be increased in inflammatory diseases such as idiopathic pulmonary fibrosis^40^ and atopic keratoconjunctitivitis^41^. Higher cutoff point of serum POSTN in CRSwNP than asthma might reflect local inflammation in nasal polyps. The level of serum POSTN in healthy controls averages 66.1 ng/mL, as reported by Kimura *et al*.^42^. The JESREC score used in the present study is calculated based on unilateral or bilateral disease, the presence of NPs, the dominant shadow of ethmoid sinuses in CT scans, and the eosinophil ratio in peripheral blood^7^. In the presence of nasal polyps, serum POSTN levels were higher even in the non-ECRS group than in healthy controls. As reported by Tokunaga *et al*., the sensitivity and specificity of JESREC in distinguishing ECRS from non-ECRS were 83% and 66%, respectively^7^. The heterogeneity of CRS is now widely recognized, and as reviewed by Dennis *et al*., there are 4 distinct but overlapping classification schemes for defining endotypes of CRSwNP: the type 2 cytokine-based, eosinophil-based, IgE-based, and cysteinyl based approach^43^. Measurement of serum POSTN is simple and can be easily performed in hospitals and clinics, and our results suggested that serum POSTN is a biomarker of postoperative CRSwNP recurrence.

In the present study, we observed a statistically significant difference between serum POSTN levels in patients with CRSwNP and asthma and those without asthma. Serum POSTN levels are reportedly high in patients with CRSwNP and asthma^44^, but few reports have actually examined levels according to asthma status. Maxfield *et al*. examined serum POSTN levels in CRSwNP, CRSsNP, healthy subjects, and subjects who underwent endoscopic nasal surgery for conditions other than CRS^45^, and average serum POSTN levels were higher in CRSwNP with asthma than without, but it did not reach the statistical significance^45^. Our data support that asthma status influences serum POSTN levels in patients with CRSwNP.

In clinical trials, asthma exacerbations were effectively controlled by lebrikizumab treatment in patients with high serum POSTN levels, and lebrikizumab significantly improved FEV1 in these patients^46^. Additionally, Seshadri *et al*. reported a positive correlation between POSTN and IL-13 expression in NPs^36^. Therefore, lebrikizumab may be effective against refractory CRSwNP with high serum POSTN levels.

Recently, Wang *et al*. compared the expression levels of ECRSwNP with those of non-ECRSwNP by RNA-seq analysis, and found that gene expressions in ECRSwNP and non-ECRSwNP displayed distinct transcriptome profiles^47^. In Wang’s study, 41 genes with over 16-folds difference between ECRSwNP and non-ECRSwNP were reported; among them, *CST1* and *CCL18* were also differentially expressed between NP and IT in the present study. In Wang’s study, *POSTN* was not detected as a differentially expressed gene although they also examined gene expression levels in control tissues. This discrepancy may be due to the difference of control samples; we used IT while sphenoid mucosal tissues were collected as controls during endoscopic trans-sphenoid removal of nonfunctioning pituitary adenomas in the study by Wang *et al*.^47^.

In conclusion, we identified genes differentially expressed in NP compared with IT by using RNA-seq technologies. Protein expression levels of POSTN were associated with the severity of CRSwNP, and serum POSTN can be a novel biomarker for the postoperative recurrence of CRSwNP.

## Methods

### Ethics Statement

Informed consent was obtained from all of the patients. The ethical committees of Department of the Otorhinolaryngology Head & Neck Surgery, University of Fukui; Department of Otolaryngology Head & Neck Surgery, Okayama University Graduate School of Medicine; Department of Otolaryngology, Jichi Medical University, Saitama Medical Center; Department of Otorhinolaryngology Head & Neck Surgery, Dokkyo Medical University and Department of Otorhinolaryngology, Yokohama City Medical Center approved this research and study protocol. All methods were performed in accordance with the relevant guidelines and regulations.

### Patients and Sample collections

Patients with CRSwNP were diagnosed based on the clinical definition of rhinosinusitis in adults, EPOS 2012^2^. CRSwNP were divided into 4 subtypes: non-ECRS, mild ECRS, moderate ECRS and severe ECRS following the criteria of JESREC study^7^. The study design and characteristics of subjects are shown in Table 1 and Fig. 1. In the RNA-seq group, 6 patients with CRSwNP undergoing the functional endoscopic sinus surgery were recruited at the Department of the Otorhinolaryngology Head & Neck Surgery, University of Fukui from January to September 2014. Fresh tissue samples of both NPs and ITs were collected from these 6 patients at the time of the surgery, and parts of the samples were immediately submerged in RNA *later*® (Thermo Fisher Scientific, Inc., Waltham, MA, USA) and stored at −80 °C until use. These tissue samples were partly formalin-fixed and paraffin-embedded. In the replication group, 110 independent patients with CRSwNP undergoing functional endoscopic sinus surgery were also recruited at the Department of the Otorhinolaryngology Head & Neck Surgery, University of Fukui between January 1996 and April 2015. Fresh tissue samples of NPs and ITs were similarly collected and used for immunohistochemistry. Additionally, we collected 4 IT mucosa from independent patients undergoing septoplasty as controls for immunohistochemistry, none of whom suffered from CRSwNP nor allergic diseases. Fourteen paired NP and IT samples were used for qPCR analysis to validate the RNA-seq results. Serum samples from another 369 patients with CRSwNP undergoing functional endoscopic sinus surgery were collected at the 5 university hospitals (Department of the Otorhinolaryngology Head & Neck Surgery, University of Fukui; Department of Otolaryngology Head & Neck Surgery, Okayama University Graduate School of Medicine; Department of Otolaryngology, Jichi Medical University, Saitama Medical Center; Department of Otorhinolaryngology Head & Neck Surgery, Dokkyo Medical University and Department of Otorhinolaryngology, Yokohama City Medical Center) and stored at −20 °C until use. Postoperative recurrence was defined as the occurrence of condition with NPs or purulent discharge in middle meatus continuing for more than 28 days after the surgery and otorhinolaryngologists confirmed the diagnosis of postoperative recurrence using nasal endoscope.

### Total RNA extraction

Frozen tissue specimens in RNA *later*® were transferred in 3 ml tubes (Yasui-kiki, Osaka, Japan) and immediately placed in liquid nitrogen. Then, tissue samples were powdered using Multi Beads Shocker® (Yasui-kiki) at 1700 rpm for 10 s. The powdered samples were dissolved in 1 ml TRIzol® reagent (Thermo Fisher Scientific Inc.) and RNA was isolated following the manufacturer’s protocol. Total RNA was extracted using Maxwell® 16 LEV simplyRNA Cells and Tissue Kit (Promega Corporation, Madison, WI, USA) following the manufacturer’s protocol or instruction, and eluted with 50 µl nuclease-free water. The extracted RNA was run on Agilent 2100 Bioanalyzer (Agilent Technologies, Inc. Santa Clara, CA, USA) to determine the quality of RNA. The RNA integrity numbers (RIN) were calculated using the Agilent 2100 Expert Software (Agilent Technologies). The RNA samples with RIN > 7 were used for RNA-seq and qPCR analysis.

### RNA-seq experiment and data analysis

Libraries for sequencing were prepared with the total RNA using TruSeq RNA Sample Prep Kit (Illumina, Inc., San Diego, CA, USA) following the manufacturer’s protocol. Paired-end sequencing was performed with Illumina HiSeq. 2000 platform (Illumina). The raw data were mapped to the human reference genome (hg19) using TopHat 2.0.14^48^. The total number of reads aligned to each gene was obtained using the featureCounts function implemented in the Subread package for paired-end reads^49^. The read counts were imported into RStudio version 3.4.0 and processed with edgeR functions^50^. Differential expression analysis was individually performed on each paired sample using a negative binomial generalized linear model^51^. Fragments per kilobase of transcript per million mapped fragments (FPKM) values were calculated using the rpkm function in the edgeR package^52^. The cut-off value of differential expression was set based on the false discovery rate (*q* values, FDR)^53^ to *P* < 0.05 and a logarithm base 2 log fold change of count per million (CPM) mapped reads >1 or <−1. The datasets of the read counts are available at National Bioscience Database Center (NBDC, https://biosciencedbc.jp/ Research ID: hum0128).

### Quantitative real time PCR

Reverse transcription and cDNA synthesis reactions were performed using 500 ng of total RNA with High-Capacity cDNA Reverse Transcription Kit (Thermo Fisher Scientific Inc.) following the manufacturer’s protocol. qPCR was performed using the TaqMan® Gene Expression Assays (Thermo Fisher Scientific Inc.): *POSTN* (Hs01566734_m1), *ALOX15* (Hs00993765_g1), *CST1* (Hs00606961_m1), *SERPINB3* (Hs00199468_m1), *CCL18* (Hs00268113_m1), *BPIFA1* (Hs00213177_m1), *SLPI* (Hs00268204_m1), *BPIFB1* (Hs00264197_m1), *STATH* (Hs00162389_m1) and *LTF* (Hs00914334_m1). The qPCR reaction mixture consisted of 5 µl qPCR QuickGoldStar Mastermix Plus (Eurogentec, Seraing, Belgium), 0.5 µl TaqMan® Gene Expression Assays, 0.5 µl nuclease free water and 4 µl diluted cDNA. The PCR reactions were 95 °C for 10 min, 40 cycles at 92 °C for 15 s and 60 °C for 1 min. *GAPDH* was used as an internal standard using Pre-Developed TaqMan® Assay Reagents Human GAPDH (Thermo Fisher Scientific Inc.). Relative gene expression was calculated using the comparative Ct method (ΔΔCT Method) and normalized to that of *GAPDH* as an endogenous control^54^.

### POSTN immunohistochemistry and ELISA

POSTN immunohistochemistry was performed as described previously^26^. Briefly, deparaffinized specimens were incubated with 0.2 µg/ml of mouse anti-POSTN monoclonal antibody (clone SS19C) at 4 °C overnight, followed by Horseradish peroxidase conjugated anti-rabbit/ mouse IgG secondary antibody (EnVision: Dako, Glostrup, Denmark) at RT for 1 h, and 3,3-diaminobenzidine tetrahydrochloride (DAB) development for approximately 20 s. The mouse anti-FLAG antibody was used as a control primary antibody, yielding no significant DAB staining. Some slides were stained at different days for intraexperimental reference purposes. The POSTN protein expressions in NPs were categorized based on the deposition patterns reported by Shiono *et al*.^23^ (see Supplementary Fig. S1); superficial type, expressed only in the subepithelial layer; diffuse type, expressed throughout the lamina propria starting just below the basement membrane. Serum POSTN level was measured using POSTN ELISA Kit (Human) (Shino-test, Tokyo, Japan) according to the manufacturer’s protocol. The values were calculated at the absorbance of 450 nm.

### Statistical analysis

Result of qPCR was analyzed using Wilcoxon rank sum test. We also performed simple linear regression analysis to determine whether gender differences influenced gene expression. Differences in immunohistochemical staining patterns between the non-ECRS and each ECRS group were assessed by chi-squared tests, and the Bonferroni correction was applied to comparisons between multiple groups. Comparisons between serum POSTN levels in CRSwNP with and without asthma were made using the Mann-Whitney test. The comparisons among the serum POSTN in each group of CRS was performed using Steel-Dwass multiple comparisons test. Correlation between serum POSTN and serum eosinophil was assessed using Spearman’s rank correlation. To analyze the relation between serum POSTN and postoperative recurrence, ROC curves were used, and the closest point to top-left of ROC was determined as the optimal cut-off point^24^. Relapse-free survival curves of postoperative recurrence were drawn using the Kaplan-Meier method. A *P* value or an adjusted *P* value of < 0.05 was considered statistically significant.

## Electronic supplementary material

Supplementary Information
