# Supplementary material for: Periostin as a novel biomarker for postoperative recurrence of chronic rhinosinitis with nasal polyps
Source: Sci Rep. 2018 Jul 30;8:11450. doi: 10.1038/s41598-018-29612-2 (PMC6065353; doi:10.1038/s41598-018-29612-2)
Supplement: Supplementary file 1 — Supplementary Information [file 41598_2018_29612_MOESM1_ESM.pdf]

Supplementary Information

Periostin as a novel biomarker for postoperative recurrence of chronic rhinosinitis with nasal polyps

Takahiro Ninomiya MD[a], Emiko Noguchi MD, PhD*[b], Takenori Haruna MD[c], Masayo Hasegawa MD[d], Takuto Yoshida MD, PhD[e], Yukiko Yamashita MD, PhD[f], Mitsuhiro Okano MD, PhD[g], Naohiro Yoshida MD, PhD[d], Shinichi Haruna MD, PhD[e], Yasunori Sakuma MD, PhD[f], Shoichiro Ohta MD, PhD[h], Junya Ono [i, j], Kenji Izuhara MD, PhD[h], Masafumi Okada MD, PhD[k], Masanori Kidoguchi MD[a], Takahiro Tokunaga MD, PhD[a], Masayuki Okamoto MD, PhD[a], Masafumi Kanno MD, PhD[a], Masafumi Sakashita MD, PhD[a], Tetsuji Takabayashi MD, PhD[a], Norihiko Narita MD, PhD[a], Shigeharu Fujieda MD, PhD[a]

[a] Departments of Otorhinolaryngology Head & Neck Surgery, Faculty of Medical Sciences, University of Fukui, Fukui, Japan.

[b] Department of Medical Genetics, Faculty of Medicine, University of Tsukuba, Tsukuba, Japan.

[c] Department of Otolaryngology-Head and Neck Surgery, Okayama University Graduate School of Medicine, Dentistry and Pharmaceutical Sciences, Okayama, Japan.

[d] Department of Otolaryngology, Jichi Medical University, Saitama Medical Center, Saitama, Japan.

[e] Department of Otorhinolaryngology Head & Neck Surgery, Dokkyo Medical University,

Tochigi, Japan

[f] Department of Otorhinolaryngology, Yokohama City University Medical Center, Kanagawa, Japan.

[g] Division of Medical Biochemistry, Department of Biomolecular Sciences, Saga Medical School, Saga, Japan.

[h] Department of Laboratory Medicine, Department of Biomolecular Sciences, Saga Medical School, Saga, Japan.

[i] Shino-test Co. Ltd., Sagamihara, Japan.

[j] University Hospital Medical Information Network Research Center, University of Tokyo, Tokyo, Japan.

Correspondence to:

Emiko Noguchi, MD, PhD

Department of Medical Genetics, Faculty of Medicine, University of Tsukuba, Ibaraki, Japan, 1-1-1, Tennoudai, Tsukuba-city, Ibaraki, 305-8575, Japan

Tel.: (+81)-29-853-3177, Fax: (+81)-29-853-3333

E-mail: enoguchi@md.tsukuba.ac.jp

Supplementary Figure and Tables

Supplementary Figure S1

The categories of periostin deposition pattern.

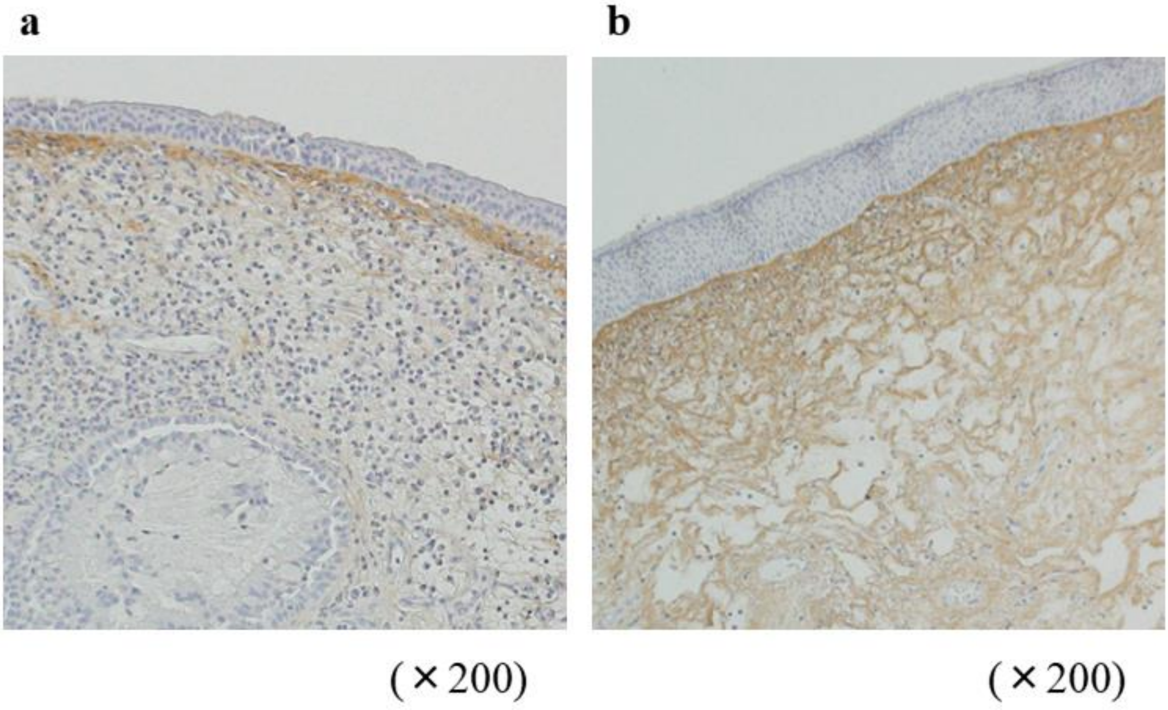

(a) Superficial type: expressed only in the subepithelial layer.

(b) Diffuse type: expressed throughout the lamina propria starting just below the basement membrane.

Supplementary Figure S2

Relative expression levels of the 10 genes by qPCR analysis in the RNA-seq group (n=6). Bars show the median values and interquartile range.

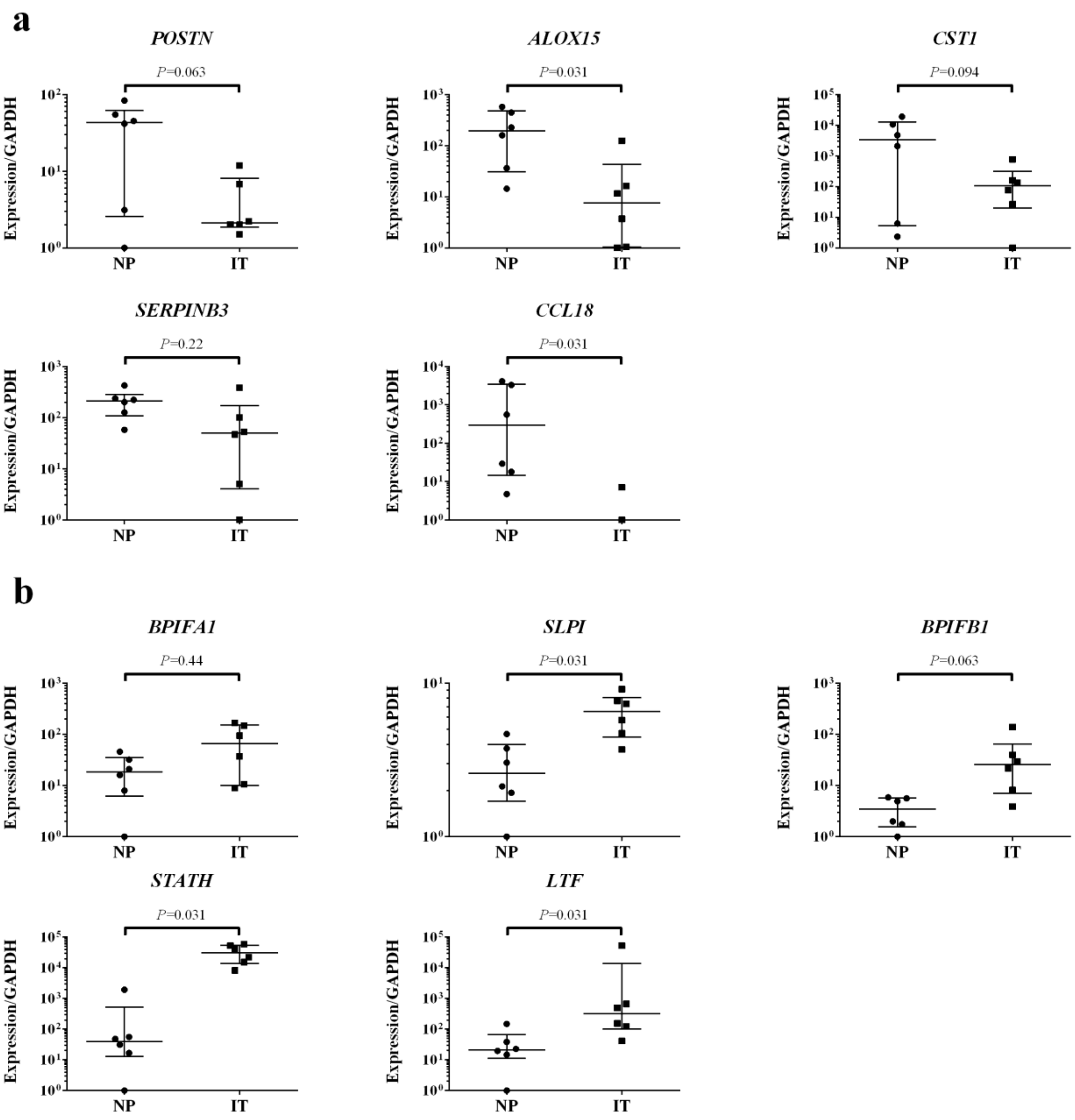

(a) The upregulated genes detected by RNA-seq (*POSTN*, *ALOX15*, *CST1*, *SERPINB3* and *CCL18*) were validated by qPCR analysis.

(b) The donwregulated genes detected by RNA-seq (*BPIFA1*, *SLPI*, *BPIFB1*, *STATH* and *LTF*) were validated by qPCR analysis.

NP, nasal polyp; IT, inferior turbinate; *POSTN*, periostin; *ALOX15*, arachidonate 15-lipoxygenase; *CST1*, cystatin SN; *SERPINB3*, serpin peptidase inhibitor, clade B (ovalbumin), member3; *CCL18*, C-C motif chemokine ligand 18; *BPIFA1*, BPI fold containing family A member 1; *SLPI*, secretory leukocyte peptidase inhibitor; *BPIFB1*, BPI fold containing family B member 1; *STATH*, statherin; *LTF*, lactotransferrin

Supplementary Figure S3

Kaplan–Meier curves of the relapse-free rate for non-ECRS at the cutoff point of serum periostin 115.5 ng/ml; log-rank test: *P* = 0.115.

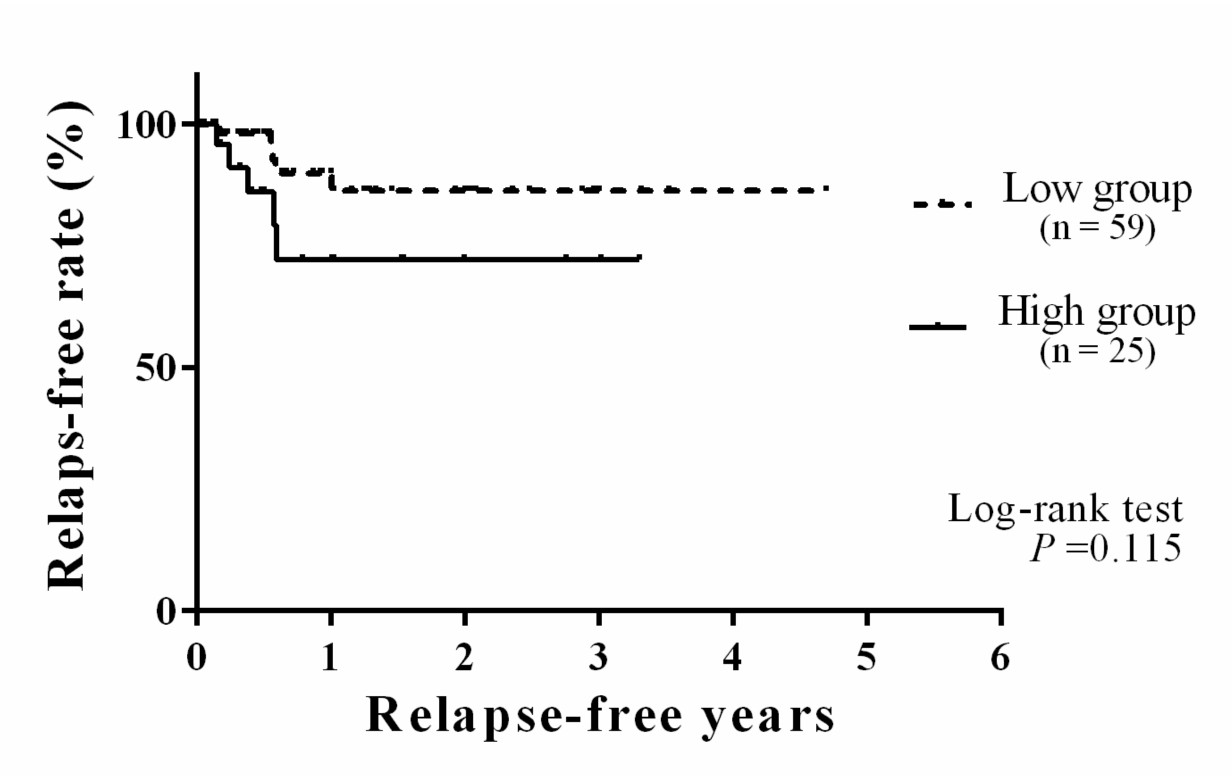

Supplementary Table S1

Upregulated genes in NP by comparison with those in IT (log fold change > 1).

Gene name	P value	Adjusted P value (FDR)	Log fold change	NP (FPKM)	IT (FPKM)
POSTN	4.02×10^{-4}	3.58×10^{-3}	3.11	7.98×10^{2}	4.96×10
ALOX15	3.92×10^{-3}	2.21×10^{-2}	3.07	6.34×10^{2}	8.02×10
CST1	4.03×10^{-3}	2.26×10^{-2}	4.77	6.29×10^{2}	8.53
SERPINB3	8.30×10^{-4}	6.41×10^{-3}	2.90	5.45×10^{2}	1.31×10^{2}
CCL18	1.23×10^{-11}	1.07×10^{-9}	6.83	3.94×10^{2}	9.27×10^{-1}
IGFBP3	5.20×10^{-20}	2.53×10^{-17}	2.86	2.60×10^{2}	4.07×10
TUBB4B	3.56×10^{-3}	2.06×10^{-2}	1.01	2.42×10^{2}	1.25×10^{2}
CSTB	1.49×10^{-5}	2.64×10^{-4}	1.21	2.10×10^{2}	8.21×10
SERPINF1	1.12×10^{-5}	2.11×10^{-4}	1.21	2.08×10^{2}	9.89×10
TPPP3	1.39×10^{-4}	1.58×10^{-3}	2.69	2.04×10^{2}	6.64×10
SRGN	9.43×10^{-4}	7.05×10^{-3}	1.68	1.96×10^{2}	4.56×10

CXCL1	5.12×10^{-3}	2.72×10^{-2}	1.92	1.92×10^{2}	3.46×10
CLC	3.20×10^{-10}	2.03×10^{-8}	5.63	1.85×10^{2}	1.82
LUM	2.33×10^{-9}	1.23×10^{-7}	1.78	1.82×10^{2}	5.49×10
S100A9	1.23×10^{-3}	8.78×10^{-3}	2.75	1.75×10^{2}	1.36×10
VMO1	1.37×10^{-7}	4.57×10^{-6}	2.72	1.66×10^{2}	1.92×10
IFI30	4.25×10^{-6}	9.44×10^{-5}	1.69	1.64×10^{2}	4.16×10
PIM2	4.36×10^{-3}	2.40×10^{-2}	1.25	1.52×10^{2}	5.23×10
CAPN13	4.37×10^{-8}	1.69×10^{-6}	3.03	1.46×10^{2}	2.10×10
MT1X	7.82×10^{-8}	2.82×10^{-6}	1.23	1.38×10^{2}	5.58×10
MMP10	4.61×10^{-4}	4.02×10^{-3}	2.69	1.37×10^{2}	3.20×10
C20orf85	1.84×10^{-4}	1.97×10^{-3}	4.17	1.34×10^{2}	3.27×10
CYBA	3.35×10^{-5}	5.19×10^{-4}	1.23	1.34×10^{2}	5.58×10
SLC6A6	2.09×10^{-6}	5.10×10^{-5}	1.34	1.34×10^{2}	5.38×10
LAPTM5	7.24×10^{-6}	1.48×10^{-4}	1.66	1.23×10^{2}	3.56×10
COTL1	8.82×10^{-9}	4.18×10^{-7}	1.99	1.12×10^{2}	2.44×10

CORO1A	3.94×10^{-6}	8.88×10^{-5}	1.91	1.07×10^{2}	2.79×10
S100A2	5.19×10^{-3}	2.76×10^{-2}	1.73	1.04×10^{2}	5.08×10
ARPC1B	1.01×10^{-6}	2.70×10^{-5}	1.12	1.02×10^{2}	4.51×10
UCP2	4.89×10^{-5}	6.93×10^{-4}	1.37	1.01×10^{2}	3.99×10
CCDC17	6.55×10^{-4}	5.32×10^{-3}	2.37	1.01×10^{2}	4.22×10
CES1	1.64×10^{-4}	1.81×10^{-3}	1.92	1.01×10^{2}	2.74×10
F13A1	2.47×10^{-4}	2.49×10^{-3}	2.07	9.96×10	1.66×10
RSPH1	2.15×10^{-5}	3.59×10^{-4}	3.22	9.86×10	3.19×10
CYP4B1	5.62×10^{-5}	7.72×10^{-4}	2.32	9.82×10	3.13×10
TGFBI	5.93×10^{-3}	3.08×10^{-2}	1.02	9.48×10	4.45×10
TNC	7.06×10^{-3}	3.51×10^{-2}	1.63	9.11×10	4.74×10
RAC2	1.73×10^{-5}	3.00×10^{-4}	1.61	8.71×10	2.86×10
NOS2	9.38×10^{-5}	1.15×10^{-3}	4.11	8.64×10	7.62
LCP1	8.84×10^{-6}	1.76×10^{-4}	1.49	8.41×10	2.69×10
HCLS1	7.47×10^{-5}	9.61×10^{-4}	1.23	7.98×10	3.44×10

SERPINB4	2.50×10^{-4}	2.51×10^{-3}	3.16	7.92×10	1.27×10
FCER1G	5.22×10^{-4}	4.44×10^{-3}	1.67	7.91×10	2.15×10
GABRP	2.64×10^{-4}	2.61×10^{-3}	1.84	7.84×10	2.61×10
TYROBP	5.62×10^{-6}	1.19×10^{-4}	1.95	7.83×10	1.86×10
CCDC78	2.29×10^{-4}	2.34×10^{-3}	2.42	7.68×10	2.68×10
CCL13	1.47×10^{-9}	8.13×10^{-8}	4.06	7.52×10	2.04
ITGB2	6.35×10^{-6}	1.32×10^{-4}	2.02	7.11×10	1.54×10
PTRH1	1.11×10^{-2}	4.97×10^{-2}	1.45	7.06×10	3.93×10
C1QC	2.17×10^{-3}	1.38×10^{-2}	1.47	7.04×10	2.27×10
CD68	5.42×10^{-4}	4.58×10^{-3}	1.47	6.98×10	2.22×10
CETN2	3.25×10^{-3}	1.91×10^{-2}	1.31	6.84×10	3.04×10
CD53	4.69×10^{-5}	6.70×10^{-4}	1.47	6.80×10	2.42×10
S100A8	2.37×10^{-3}	1.49×10^{-2}	2.95	6.76×10	3.26
CPXM1	3.75×10^{-3}	2.14×10^{-2}	1.28	6.75×10	3.02×10
C1QB	2.57×10^{-3}	1.59×10^{-2}	1.49	6.53×10	2.05×10

TMEM176B	7.60×10^{-4}	6.02×10^{-3}	1.02	6.51×10	2.95×10
CFAP157	2.20×10^{-4}	2.27×10^{-3}	2.64	6.44×10	3.46×10
PP7080	4.25×10^{-6}	9.44×10^{-5}	1.40	6.33×10	2.37×10
MIR205HG	2.19×10^{-9}	1.17×10^{-7}	1.70	6.26×10	2.18×10
CD37	3.06×10^{-12}	2.92×10^{-10}	1.56	6.09×10	2.10×10
FOXJ1	5.34×10^{-5}	7.43×10^{-4}	3.58	5.90×10	1.93×10
HLA-DQA1	5.26×10^{-8}	1.98×10^{-6}	1.40	5.75×10	2.19×10
TMEM176A	4.95×10^{-4}	4.25×10^{-3}	1.04	5.71×10	2.61×10
TMEM190	1.06×10^{-5}	2.03×10^{-4}	3.86	5.61×10	9.41
PTP4A3	3.73×10^{-4}	3.38×10^{-3}	1.29	5.49×10	1.87×10
CTSS	2.17×10^{-4}	2.25×10^{-3}	1.13	5.47×10	2.37×10
NCF2	3.46×10^{-6}	7.94×10^{-5}	2.71	5.45×10	6.28
CPA3	7.74×10^{-13}	8.62×10^{-11}	1.97	5.43×10	1.42×10
DHRS3	1.15×10^{-4}	1.36×10^{-3}	1.05	5.39×10	2.69×10
LAMP5	3.09×10^{-6}	7.19×10^{-5}	2.52	5.38×10	8.65

ARRB2	4.68×10^{-5}	6.70×10^{-4}	1.35	5.36×10	1.96×10
PTPN6	2.73×10^{-5}	4.40×10^{-4}	1.20	5.34×10	2.31×10
APOE	4.09×10^{-4}	3.64×10^{-3}	1.32	5.16×10	2.13×10
BASP1	3.81×10^{-5}	5.76×10^{-4}	1.60	5.14×10	1.86×10
THY1	4.79×10^{-3}	2.58×10^{-2}	1.42	5.10×10	2.22×10
SNTN	7.40×10^{-5}	9.53×10^{-4}	3.74	4.93×10	1.71×10
ZMYND10	2.65×10^{-5}	4.31×10^{-4}	2.85	4.92×10	1.67×10
ALDH3B1	1.84×10^{-3}	1.21×10^{-2}	1.64	4.90×10	2.41×10
JAML	9.56×10^{-5}	1.17×10^{-3}	2.15	4.87×10	9.05
C1QA	4.57×10^{-3}	2.49×10^{-2}	1.32	4.87×10	1.98×10
DUOX1	6.99×10^{-9}	3.37×10^{-7}	1.67	4.86×10	1.85×10
CSF2RB	3.09×10^{-3}	1.84×10^{-2}	1.39	4.85×10	1.73×10
EVI2B	9.68×10^{-4}	7.19×10^{-3}	1.43	4.75×10	2.17×10
GMFG	1.11×10^{-5}	2.10×10^{-4}	1.52	4.72×10	1.61×10
RDH10	1.39×10^{-6}	3.56×10^{-5}	1.26	4.66×10	2.04×10

CLDN1	7.72×10^{-10}	4.56×10^{-8}	1.08	4.58×10	2.36×10
SULF1	1.17×10^{-18}	4.81×10^{-16}	2.01	4.54×10	1.10×10
CECR1	7.90×10^{-4}	6.19×10^{-3}	1.25	4.53×10	2.14×10
CFI	6.65×10^{-15}	1.10×10^{-12}	2.18	4.52×10	9.25
DNAAF1	1.08×10^{-5}	2.05×10^{-4}	2.86	4.50×10	1.76×10
ADAM8	3.29×10^{-4}	3.08×10^{-3}	1.76	4.48×10	1.02×10
LRRC23	1.12×10^{-3}	8.10×10^{-3}	1.90	4.46×10	1.86×10
IL33	2.23×10^{-4}	2.29×10^{-3}	1.04	4.44×10	2.34×10
MAPK15	6.22×10^{-4}	5.12×10^{-3}	1.93	4.35×10	2.49×10
MS4A8	1.56×10^{-5}	2.74×10^{-4}	4.06	4.33×10	1.71×10
DHRS9	6.51×10^{-8}	2.39×10^{-6}	2.36	4.33×10	1.22×10
LRRC46	1.08×10^{-4}	1.29×10^{-3}	2.99	4.30×10	1.62×10
FANK1	1.61×10^{-4}	1.77×10^{-3}	2.46	4.30×10	1.55×10
PTPRCAP	3.74×10^{-5}	5.66×10^{-4}	1.03	4.27×10	2.17×10
LSP1	1.80×10^{-5}	3.08×10^{-4}	1.20	4.27×10	1.81×10

CFAP45	1.60×10^{-5}	2.80×10^{-4}	2.90	4.26×10	1.47×10
CEACAM5	9.81×10^{-3}	4.52×10^{-2}	1.69	4.22×10	1.00×10
C1orf194	8.04×10^{-5}	1.02×10^{-3}	3.60	4.22×10	1.27×10
CD52	2.33×10^{-10}	1.54×10^{-8}	2.05	4.21×10	1.05×10
TMPRSS4	1.02×10^{-3}	7.50×10^{-3}	1.63	4.18×10	1.87×10
SPI1	5.62×10^{-5}	7.72×10^{-4}	1.95	4.18×10	9.36
TPSB2	4.42×10^{-5}	6.39×10^{-4}	1.36	4.15×10	1.53×10
WDR38	1.07×10^{-4}	1.29×10^{-3}	4.06	4.14×10	1.29×10
DLEC1	3.98×10^{-5}	5.93×10^{-4}	2.51	4.12×10	1.91×10
FAM166B	3.04×10^{-5}	4.80×10^{-4}	3.23	4.08×10	1.44×10
CDHR3	1.16×10^{-4}	1.37×10^{-3}	3.42	4.06×10	1.83×10
FAM183A	3.33×10^{-5}	5.16×10^{-4}	2.96	4.05×10	1.29×10
DNALI1	6.23×10^{-4}	5.12×10^{-3}	1.65	4.03×10	1.61×10
NCF4	1.98×10^{-3}	1.29×10^{-2}	1.40	4.03×10	1.48×10
C11orf88	1.41×10^{-5}	2.54×10^{-4}	3.77	3.99×10	9.79

CFAP126	2.43×10^{-4}	2.46×10^{-3}	2.04	3.98×10	1.25×10
MORN2	4.42×10^{-3}	2.42×10^{-2}	1.46	3.95×10	1.58×10
HS3ST1	3.41×10^{-8}	1.38×10^{-6}	1.95	3.95×10	1.17×10
FGFR3	3.59×10^{-3}	2.07×10^{-2}	1.55	3.95×10	1.93×10
PIFO	1.62×10^{-4}	1.78×10^{-3}	2.71	3.94×10	1.44×10
C9orf116	3.14×10^{-4}	2.97×10^{-3}	2.48	3.91×10	1.14×10
MIR223	1.79×10^{-5}	3.07×10^{-4}	4.07	3.91×10	2.54
CCL24	3.09×10^{-5}	4.87×10^{-4}	3.60	3.90×10	2.85
CTSC	2.40×10^{-4}	2.42×10^{-3}	1.39	3.89×10	1.29×10
PECAM1	1.06×10^{-7}	3.66×10^{-6}	1.08	3.84×10	1.83×10
ROPN1L	3.15×10^{-5}	4.94×10^{-4}	4.17	3.84×10	8.80
CDHR4	3.15×10^{-4}	2.98×10^{-3}	3.51	3.84×10	1.67×10
PLCB2	9.50×10^{-5}	1.17×10^{-3}	1.97	3.83×10	8.75
ALOX5	1.76×10^{-4}	1.91×10^{-3}	1.47	3.82×10	1.52×10
HMOX1	1.82×10^{-4}	1.95×10^{-3}	1.67	3.78×10	1.13×10

SLC16A3	$8.95×10^{-6}$	$1.77×10^{-4}$	1.72	3.78×10	1.16×10
ADAM28	$5.28×10^{-8}$	$1.98×10^{-6}$	1.47	3.72×10	1.20×10
LDLRAD1	$2.97×10^{-3}$	$1.79×10^{-2}$	2.00	3.67×10	1.62×10
CYP2B7P	$5.11×10^{-3}$	$2.72×10^{-2}$	1.87	3.64×10	1.62×10
ITGAX	$1.52×10^{-4}$	$1.69×10^{-3}$	1.74	3.60×10	8.90
COL8A1	$1.42×10^{-5}$	$2.54×10^{-4}$	2.01	3.59×10	6.42
WFDC21P	$1.31×10^{-3}$	$9.26×10^{-3}$	1.35	3.58×10	1.24×10
TMEM231	$6.37×10^{-4}$	$5.21×10^{-3}$	1.70	3.57×10	1.54×10
CDH26	$2.82×10^{-12}$	$2.74×10^{-10}$	3.61	3.56×10	2.42
LAMB3	$2.13×10^{-11}$	$1.75×10^{-9}$	1.38	3.54×10	1.44×10
IL32	$4.10×10^{-5}$	$6.07×10^{-4}$	1.09	3.54×10	1.65×10
CSF3R	$9.76×10^{-3}$	$4.50×10^{-2}$	1.87	3.53×10	5.51
PLEK	$3.12×10^{-4}$	$2.95×10^{-3}$	1.61	3.52×10	9.38
CXCL6	$2.09×10^{-3}$	$1.34×10^{-2}$	2.85	3.51×10	1.37
HLA-DRB1	$8.44×10^{-7}$	$2.32×10^{-5}$	1.06	3.50×10	1.65×10

CCDC114	3.75×10^{-4}	3.39×10^{-3}	2.95	3.49×10	1.35×10
CSF1R	7.71×10^{-3}	3.75×10^{-2}	1.20	3.47×10	1.51×10
TPSAB1	9.32×10^{-10}	5.42×10^{-8}	1.42	3.42×10	1.38×10
SLC2A1	4.17×10^{-5}	6.14×10^{-4}	1.19	3.42×10	1.52×10
DENND6B	9.54×10^{-3}	4.41×10^{-2}	1.11	3.39×10	1.77×10
MIR142	1.09×10^{-4}	1.30×10^{-3}	1.67	3.39×10	1.15×10
FAM92B	8.39×10^{-5}	1.05×10^{-3}	3.61	3.39×10	1.18×10
LIPA	9.87×10^{-6}	1.92×10^{-4}	1.06	3.39×10	1.57×10
TEKT1	3.22×10^{-4}	3.03×10^{-3}	4.19	3.37×10	9.34
CYP2S1	8.87×10^{-6}	1.76×10^{-4}	2.42	3.36×10	9.05
MUC4	8.89×10^{-3}	4.18×10^{-2}	1.69	3.33×10	1.72×10
PTPRC	9.82×10^{-5}	1.19×10^{-3}	1.63	3.30×10	9.73
CD4	2.30×10^{-4}	2.35×10^{-3}	1.50	3.27×10	1.17×10
C5AR1	2.15×10^{-3}	1.37×10^{-2}	1.58	3.27×10	1.09×10
FMO2	4.50×10^{-9}	2.27×10^{-7}	1.57	3.26×10	1.07×10

PTHLH	3.63×10^{-9}	1.87×10^{-7}	2.36	3.25×10	7.34
IQCG	6.96×10^{-4}	5.61×10^{-3}	1.77	3.22×10	1.30×10
DRC1	3.62×10^{-5}	5.52×10^{-4}	4.04	3.21×10	9.65
CD163	6.16×10^{-3}	3.17×10^{-2}	1.71	3.19×10	8.01
SCG2	1.74×10^{-3}	1.16×10^{-2}	2.38	3.17×10	5.63
CYBB	1.81×10^{-4}	1.94×10^{-3}	2.04	3.16×10	7.59
NWD1	9.99×10^{-7}	2.68×10^{-5}	2.35	3.15×10	9.01
CFAP100	7.76×10^{-4}	6.12×10^{-3}	3.52	3.13×10	1.35×10
ARHGAP30	1.16×10^{-3}	8.32×10^{-3}	1.14	3.13×10	1.42×10
HCK	6.97×10^{-5}	9.11×10^{-4}	2.23	3.08×10	5.48
DNAI1	2.18×10^{-4}	2.26×10^{-3}	3.91	3.07×10	1.23×10
CCDC146	8.93×10^{-3}	4.20×10^{-2}	1.15	3.06×10	1.63×10
TEKT2	2.63×10^{-5}	4.28×10^{-4}	2.86	3.06×10	1.35×10
SASH3	6.07×10^{-5}	8.16×10^{-4}	1.70	3.06×10	1.10×10
MYO1F	2.71×10^{-4}	2.66×10^{-3}	1.83	3.04×10	8.28

LOC100288152	9.67×10^{-6}	1.89×10^{-4}	1.13	3.03×10	1.43×10
SELL	3.43×10^{-9}	1.78×10^{-7}	2.18	3.03×10	5.65
HEG1	3.76×10^{-6}	8.52×10^{-5}	1.13	2.99×10	1.32×10
WDR86-AS1	4.87×10^{-4}	4.20×10^{-3}	2.38	2.98×10	1.29×10
PDGFRA	9.86×10^{-5}	1.20×10^{-3}	1.07	2.92×10	1.43×10
RHOV	1.09×10^{-7}	3.73×10^{-6}	1.14	2.89×10	1.24×10
RASSF2	6.09×10^{-5}	8.17×10^{-4}	1.40	2.89×10	1.04×10
LOC101927416	1.03×10^{-5}	1.98×10^{-4}	4.77	2.88×10	6.68
PCP4L1	1.38×10^{-4}	1.57×10^{-3}	1.64	2.87×10	1.08×10
SELPLG	6.93×10^{-4}	5.59×10^{-3}	1.66	2.87×10	8.37
CYP24A1	1.68×10^{-3}	1.13×10^{-2}	2.32	2.84×10	5.22
ARL4C	1.02×10^{-3}	7.53×10^{-3}	1.28	2.84×10	9.20
FPR1	5.23×10^{-3}	2.78×10^{-2}	2.21	2.83×10	3.63
FGR	4.70×10^{-4}	4.07×10^{-3}	1.71	2.82×10	7.56
VWA3A	1.69×10^{-4}	1.84×10^{-3}	3.24	2.81×10	1.24×10

WISP1	1.52×10^{-6}	3.86×10^{-5}	2.72	2.80×10	5.21
AGR3	2.23×10^{-4}	2.29×10^{-3}	2.20	2.79×10	6.94
ACAP1	1.06×10^{-3}	7.80×10^{-3}	1.01	2.78×10	1.43×10
WDR54	2.74×10^{-3}	1.67×10^{-2}	1.19	2.78×10	1.33×10
C10orf128	4.31×10^{-8}	1.68×10^{-6}	1.67	2.77×10	7.92
ALOX5AP	3.27×10^{-6}	7.57×10^{-5}	2.23	2.77×10	4.70
SLC27A2	1.36×10^{-10}	9.48×10^{-9}	2.59	2.76×10	6.50
NCF1	5.54×10^{-6}	1.18×10^{-4}	2.37	2.76×10	4.83
SPAG6	3.85×10^{-5}	5.80×10^{-4}	3.77	2.76×10	8.23
MPEG1	2.13×10^{-3}	1.36×10^{-2}	1.34	2.75×10	1.20×10
DRC3	3.94×10^{-3}	2.22×10^{-2}	1.58	2.73×10	1.48×10
SPON1	1.60×10^{-8}	7.15×10^{-7}	1.90	2.72×10	7.32
ICAM3	7.22×10^{-5}	9.38×10^{-4}	1.19	2.72×10	1.10×10
ACP5	5.22×10^{-6}	1.13×10^{-4}	1.45	2.71×10	9.44
LCP2	7.60×10^{-4}	6.02×10^{-3}	1.44	2.71×10	9.28

FAM216B	1.04×10^{-5}	1.99×10^{-4}	4.20	2.69×10	6.67
LRRC10B	5.16×10^{-5}	7.22×10^{-4}	2.89	2.69×10	7.98
EGLN3	3.04×10^{-5}	4.80×10^{-4}	1.80	2.68×10	1.00×10
CLDN11	1.26×10^{-3}	8.96×10^{-3}	1.81	2.68×10	7.95
CHST15	1.37×10^{-3}	9.61×10^{-3}	1.12	2.65×10	1.28×10
FERMT3	1.22×10^{-3}	8.71×10^{-3}	1.18	2.64×10	1.12×10
C1orf162	5.64×10^{-5}	7.73×10^{-4}	1.65	2.62×10	7.60
MS4A6A	4.51×10^{-5}	6.50×10^{-4}	1.77	2.61×10	7.78
SLCO2B1	4.42×10^{-4}	3.87×10^{-3}	1.27	2.61×10	1.10×10
SLC9A3	1.80×10^{-9}	9.77×10^{-8}	2.71	2.59×10	4.27
FOLR2	7.90×10^{-3}	3.81×10^{-2}	1.44	2.58×10	7.95
WAS	7.22×10^{-5}	9.38×10^{-4}	1.60	2.56×10	8.08
NTN1	2.26×10^{-8}	9.68×10^{-7}	1.29	2.54×10	1.10×10
CAPSL	3.60×10^{-4}	3.29×10^{-3}	4.13	2.54×10	7.97
SELP	2.20×10^{-5}	3.65×10^{-4}	1.07	2.53×10	1.26×10

BCL2A1	3.05×10^{-3}	1.82×10^{-2}	2.52	2.52×10	2.86
BIN2	3.35×10^{-4}	3.12×10^{-3}	1.59	2.51×10	8.14
MT1F	1.29×10^{-5}	2.37×10^{-4}	1.14	2.51×10	1.12×10
FCGR2A	6.67×10^{-3}	3.36×10^{-2}	1.44	2.49×10	7.45
HCST	1.08×10^{-4}	1.29×10^{-3}	1.40	2.49×10	9.39
PRR15	6.10×10^{-6}	1.28×10^{-4}	1.85	2.49×10	7.31
CDH3	2.39×10^{-4}	2.42×10^{-3}	1.08	2.49×10	1.29×10
ARHGAP9	1.09×10^{-3}	7.92×10^{-3}	1.41	2.48×10	8.30
SLA	4.82×10^{-8}	1.84×10^{-6}	1.94	2.48×10	6.57
ITGAM	1.03×10^{-4}	1.24×10^{-3}	2.54	2.48×10	3.08
GLIPR1	3.48×10^{-7}	1.05×10^{-5}	1.43	2.47×10	8.49
SERPINA1	6.55×10^{-4}	5.32×10^{-3}	1.63	2.46×10	6.19
FCRL5	4.77×10^{-3}	2.58×10^{-2}	1.34	2.46×10	9.31
SEC14L3	5.29×10^{-6}	1.14×10^{-4}	2.74	2.43×10	7.66
MT1G	3.78×10^{-14}	5.22×10^{-12}	2.61	2.41×10	3.80

CPVL	1.98×10^{-5}	3.33×10^{-4}	1.48	2.37×10	8.26
NQO1	2.76×10^{-4}	2.70×10^{-3}	1.37	2.36×10	1.18×10
THBS2	4.41×10^{-8}	1.70×10^{-6}	1.17	2.32×10	1.04×10
C12orf75	2.12×10^{-5}	3.54×10^{-4}	1.93	2.31×10	7.98
CLEC10A	1.21×10^{-6}	3.17×10^{-5}	2.40	2.28×10	3.81
LIMD2	6.41×10^{-6}	1.33×10^{-4}	1.26	2.27×10	9.39
EMB	4.60×10^{-3}	2.50×10^{-2}	1.19	2.26×10	1.13×10
RGS19	1.08×10^{-4}	1.29×10^{-3}	1.32	2.24×10	8.27
FCN1	4.97×10^{-3}	2.66×10^{-2}	2.20	2.24×10	3.53
C5orf49	1.15×10^{-5}	2.16×10^{-4}	3.20	2.23×10	6.31
C9orf24	5.11×10^{-5}	7.17×10^{-4}	3.09	2.22×10	7.34
TUBA4B	1.36×10^{-4}	1.55×10^{-3}	3.44	2.21×10	7.52
MNDA	6.63×10^{-3}	3.34×10^{-2}	2.04	2.18×10	3.82
ITGAL	2.27×10^{-3}	1.43×10^{-2}	1.10	2.18×10	1.10×10
HK3	6.96×10^{-6}	1.43×10^{-4}	3.84	2.17×10	1.01

COL10A1	1.38×10^{-11}	1.18×10^{-9}	2.61	2.16×10	3.48
MXD1	8.52×10^{-3}	4.04×10^{-2}	1.24	2.16×10	6.94
CCDC74A	9.34×10^{-4}	7.00×10^{-3}	1.58	2.16×10	9.51
CFAP73	6.47×10^{-3}	3.29×10^{-2}	1.91	2.15×10	1.01×10
GIPR	6.50×10^{-3}	3.30×10^{-2}	1.45	2.13×10	1.10×10
HAS3	1.95×10^{-6}	4.81×10^{-5}	1.63	2.12×10	7.23
PIK3CD	1.37×10^{-5}	2.48×10^{-4}	1.37	2.12×10	8.13
HIST1H1C	1.89×10^{-6}	4.69×10^{-5}	1.77	2.11×10	5.47
NKG7	2.38×10^{-5}	3.94×10^{-4}	1.40	2.11×10	8.24
PTAFR	2.71×10^{-6}	6.37×10^{-5}	2.43	2.10×10	4.03
BEST4	1.53×10^{-4}	1.70×10^{-3}	1.76	2.10×10	9.47
CD79B	2.57×10^{-7}	8.09×10^{-6}	1.46	2.09×10	9.12
MS4A1	2.55×10^{-8}	1.08×10^{-6}	1.73	2.05×10	8.00
ABCC5	3.43×10^{-5}	5.26×10^{-4}	1.04	2.05×10	1.05×10
TMC8	6.87×10^{-5}	9.00×10^{-4}	1.22	2.04×10	9.32

FCGR3A	7.78×10^{-4}	6.12×10^{-3}	1.53	2.03×10	5.94
C11orf16	1.95×10^{-5}	3.29×10^{-4}	3.62	2.03×10	6.79
PPP1R32	8.10×10^{-3}	3.88×10^{-2}	1.59	2.03×10	1.02×10
ADCY7	2.25×10^{-3}	1.42×10^{-2}	1.19	2.02×10	8.66
SAXO2	5.59×10^{-5}	7.69×10^{-4}	2.82	2.02×10	8.76
CD3E	4.34×10^{-4}	3.82×10^{-3}	1.02	2.02×10	1.00×10
PLAC8	2.18×10^{-5}	3.63×10^{-4}	2.10	2.02×10	6.08
DOK3	2.36×10^{-4}	2.40×10^{-3}	1.63	2.01×10	6.48
FAM81B	7.01×10^{-5}	9.14×10^{-4}	4.12	2.01×10	5.54
NDUFA4L2	9.93×10^{-6}	1.93×10^{-4}	1.25	2.01×10	8.72
ANKRD65	1.08×10^{-7}	3.71×10^{-6}	1.51	1.99×10	7.98
CCND2	1.09×10^{-2}	4.93×10^{-2}	1.00	1.99×10	7.57
CCDC190	3.79×10^{-4}	3.41×10^{-3}	2.89	1.98×10	6.93
RGS14	1.10×10^{-3}	7.98×10^{-3}	1.02	1.96×10	9.42
VAV1	1.41×10^{-4}	1.59×10^{-3}	1.24	1.96×10	8.41

RGL1	6.09×10^{-5}	8.17×10^{-4}	1.07	1.96×10	9.50
CFAP53	2.62×10^{-4}	2.59×10^{-3}	2.70	1.95×10	6.54
SYK	6.74×10^{-6}	1.40×10^{-4}	1.27	1.95×10	8.00
DNAI2	2.21×10^{-4}	2.28×10^{-3}	3.67	1.95×10	7.70
CFAP52	4.11×10^{-5}	6.07×10^{-4}	3.50	1.94×10	6.05
SORL1	9.32×10^{-8}	3.29×10^{-6}	1.38	1.94×10	7.30
FUZ	2.36×10^{-3}	1.48×10^{-2}	1.15	1.94×10	9.10
ARHGAP25	2.20×10^{-5}	3.65×10^{-4}	1.27	1.93×10	7.77
IMPA2	3.15×10^{-7}	9.64×10^{-6}	1.07	1.93×10	9.40
WDR86	6.55×10^{-4}	5.32×10^{-3}	1.22	1.93×10	9.37
RSPH4A	2.51×10^{-4}	2.52×10^{-3}	2.42	1.91×10	7.36
FGD2	2.90×10^{-4}	2.80×10^{-3}	1.32	1.91×10	7.94
TNFAIP8L1	2.66×10^{-4}	2.62×10^{-3}	1.98	1.89×10	6.91
MFNG	2.59×10^{-6}	6.15×10^{-5}	1.35	1.89×10	7.84
FAM179A	1.39×10^{-5}	2.50×10^{-4}	3.20	1.88×10	7.46

KIAA0125	1.06×10^{-2}	4.80×10^{-2}	1.20	1.87×10	7.58
SAMSN1	7.78×10^{-4}	6.12×10^{-3}	1.56	1.87×10	5.54
IL2RB	1.50×10^{-4}	1.67×10^{-3}	1.11	1.86×10	8.97
PRR29	2.65×10^{-4}	2.61×10^{-3}	2.33	1.85×10	8.05
LAIR1	1.80×10^{-4}	1.94×10^{-3}	1.54	1.85×10	5.87
TTC25	1.08×10^{-4}	1.29×10^{-3}	2.69	1.85×10	6.79
CIITA	7.81×10^{-7}	2.16×10^{-5}	1.05	1.85×10	8.75
NCF1B	3.25×10^{-5}	5.06×10^{-4}	2.26	1.84×10	3.82
CYP2A13	2.66×10^{-3}	1.63×10^{-2}	1.95	1.84×10	7.10
MORN5	1.46×10^{-5}	2.60×10^{-4}	3.75	1.84×10	5.58
DOCK2	3.32×10^{-4}	3.09×10^{-3}	1.36	1.83×10	7.13
DOK2	3.19×10^{-5}	4.98×10^{-4}	1.93	1.83×10	4.42
SPEF1	8.14×10^{-5}	1.03×10^{-3}	3.12	1.81×10	5.33
MS4A7	4.11×10^{-3}	2.30×10^{-2}	1.13	1.81×10	7.75
WDR66	5.58×10^{-5}	7.69×10^{-4}	2.62	1.80×10	6.63

EFCAB1	4.56×10^{-5}	6.55×10^{-4}	2.92	1.79×10	5.43
CFAP43	5.67×10^{-5}	7.75×10^{-4}	3.37	1.77×10	6.73
LAX1	5.09×10^{-3}	2.71×10^{-2}	1.27	1.76×10	7.50
NABP1	2.17×10^{-4}	2.25×10^{-3}	1.08	1.76×10	8.01
DNAAF3	1.34×10^{-4}	1.53×10^{-3}	3.87	1.76×10	6.03
ADGRE4P	3.36×10^{-5}	5.20×10^{-4}	2.93	1.75×10	1.29
PXDN	6.04×10^{-4}	5.01×10^{-3}	1.23	1.75×10	7.24
ZNF385A	4.28×10^{-4}	3.78×10^{-3}	1.10	1.75×10	9.13
CD177	1.23×10^{-22}	8.99×10^{-20}	3.44	1.74×10	1.48
GMIP	8.96×10^{-4}	6.78×10^{-3}	1.00	1.74×10	8.16
LILRB2	8.45×10^{-3}	4.01×10^{-2}	1.46	1.73×10	5.32
TMTC2	3.92×10^{-4}	3.51×10^{-3}	1.04	1.72×10	7.58
RSPH9	2.33×10^{-5}	3.85×10^{-4}	2.49	1.72×10	5.66
DUOXA1	5.36×10^{-5}	7.45×10^{-4}	2.09	1.72×10	5.49
RUVBL1	1.65×10^{-3}	1.12×10^{-2}	1.01	1.72×10	8.68

RCSD1	$2.12×10^{-4}$	$2.21×10^{-3}$	1.22	1.71×10	7.53
TP63	$1.51×10^{-5}$	$2.67×10^{-4}$	1.41	1.71×10	6.84
BTK	$1.49×10^{-5}$	$2.64×10^{-4}$	1.74	1.70×10	5.43
TEX26	$9.40×10^{-6}$	$1.85×10^{-4}$	4.39	1.69×10	2.49
NCF1C	$7.29×10^{-5}$	$9.45×10^{-4}$	2.10	1.68×10	3.67
MS4A4A	$1.31×10^{-4}$	$1.51×10^{-3}$	2.01	1.67×10	3.43
CCDC153	$8.83×10^{-4}$	$6.70×10^{-3}$	2.21	1.66×10	6.74
FAM101B	$8.55×10^{-4}$	$6.54×10^{-3}$	1.14	1.66×10	6.84
NME5	$2.91×10^{-4}$	$2.80×10^{-3}$	1.84	1.63×10	4.96
SIGLEC10	$3.87×10^{-7}$	$1.15×10^{-5}$	2.72	1.63×10	2.23
DOCK8	$4.77×10^{-5}$	$6.79×10^{-4}$	1.33	1.63×10	6.43
FABP6	$2.21×10^{-5}$	$3.68×10^{-4}$	3.82	1.62×10	5.16
ADGRG3	$8.84×10^{-6}$	$1.76×10^{-4}$	3.48	1.61×10	1.15
UBXN10	$3.64×10^{-5}$	$5.53×10^{-4}$	2.68	1.61×10	5.20
WFDC6	$3.76×10^{-4}$	$3.39×10^{-3}$	2.64	1.61×10	8.40

JAK3	$2.32{\times}10^{-4}$	$2.37{\times}10^{-3}$	1.31	1.60×10	5.98
P4HA1	$3.02{\times}10^{-8}$	$1.24{\times}10^{-6}$	1.07	1.60×10	7.57
APOBR	$1.57{\times}10^{-4}$	$1.75{\times}10^{-3}$	2.23	1.60×10	3.00
DSE	$3.28{\times}10^{-4}$	$3.07{\times}10^{-3}$	1.17	1.60×10	7.02
IL16	$2.21{\times}10^{-3}$	$1.40{\times}10^{-2}$	1.13	1.58×10	8.05
LRMP	$1.24{\times}10^{-11}$	$1.07{\times}10^{-9}$	1.94	1.58×10	4.32
PTPRE	$1.42{\times}10^{-3}$	$9.84{\times}10^{-3}$	1.00	1.57×10	7.05
CCDC65	$1.09{\times}10^{-3}$	$7.92{\times}10^{-3}$	1.74	1.56×10	6.78
PARVG	$3.34{\times}10^{-4}$	$3.11{\times}10^{-3}$	1.53	1.56×10	5.13
SPAG8	$2.05{\times}10^{-3}$	$1.32{\times}10^{-2}$	1.92	1.55×10	7.90
LRRC71	$5.87{\times}10^{-5}$	$7.94{\times}10^{-4}$	2.94	1.54×10	6.92
RASAL3	$2.48{\times}10^{-4}$	$2.50{\times}10^{-3}$	1.20	1.54×10	7.07
DAPP1	$1.58{\times}10^{-3}$	$1.08{\times}10^{-2}$	1.06	1.53×10	7.11
COLEC12	$5.78{\times}10^{-3}$	$3.01{\times}10^{-2}$	1.05	1.53×10	8.97
MARCO	$2.04{\times}10^{-4}$	$2.15{\times}10^{-3}$	4.10	1.53×10	$3.54{\times}10^{-1}$

NAPSB	4.23×10^{-9}	2.14×10^{-7}	1.41	1.53×10	6.01
BIRC3	4.94×10^{-3}	2.65×10^{-2}	1.02	1.52×10	6.99
DCDC2B	4.16×10^{-3}	2.32×10^{-2}	1.63	1.51×10	8.65
GAS2L2	3.46×10^{-3}	2.01×10^{-2}	3.37	1.50×10	6.55
RAB36	5.54×10^{-4}	4.67×10^{-3}	2.00	1.50×10	5.70
CD48	2.58×10^{-5}	4.21×10^{-4}	1.28	1.50×10	5.91
FFAR2	2.86×10^{-4}	2.77×10^{-3}	3.57	1.50×10	5.73×10^{-1}
STOML3	5.62×10^{-6}	1.19×10^{-4}	4.00	1.49×10	4.19
SPP1	2.98×10^{-3}	1.79×10^{-2}	2.27	1.48×10	2.24
C22orf15	1.39×10^{-4}	1.58×10^{-3}	3.43	1.46×10	5.83
HCAR2	1.24×10^{-3}	8.86×10^{-3}	1.31	1.44×10	6.15
MFSD2A	1.04×10^{-4}	1.25×10^{-3}	1.06	1.44×10	7.34
MYO1G	1.52×10^{-3}	1.04×10^{-2}	1.32	1.44×10	5.75
CCDC40	5.34×10^{-3}	2.82×10^{-2}	1.35	1.44×10	7.20
PSTPIP1	2.02×10^{-3}	1.30×10^{-2}	1.24	1.43×10	5.60

TBXAS1	2.59×10^{-4}	2.57×10^{-3}	1.70	1.43×10	3.93
APOC1	1.40×10^{-9}	7.79×10^{-8}	2.49	1.42×10	2.07
GNA15	1.29×10^{-3}	9.17×10^{-3}	1.15	1.42×10	6.63
SLC22A4	6.02×10^{-3}	3.11×10^{-2}	1.16	1.42×10	7.78
CD209	9.16×10^{-3}	4.27×10^{-2}	1.72	1.42×10	3.05
ABCA13	5.45×10^{-4}	4.60×10^{-3}	1.24	1.41×10	5.67
FYB	6.07×10^{-5}	8.16×10^{-4}	1.67	1.41×10	4.05
CD2	6.61×10^{-4}	5.35×10^{-3}	1.01	1.41×10	7.45
STMND1	2.30×10^{-4}	2.35×10^{-3}	4.29	1.40×10	3.75
PILRA	1.16×10^{-3}	8.32×10^{-3}	1.48	1.40×10	5.71
NAT14	3.10×10^{-3}	1.84×10^{-2}	1.26	1.39×10	6.43
SPA17	2.35×10^{-4}	2.39×10^{-3}	2.36	1.39×10	4.39
NUP50-AS1	3.90×10^{-3}	2.20×10^{-2}	1.11	1.39×10	7.55
DPEP2	6.12×10^{-5}	8.20×10^{-4}	1.68	1.39×10	4.16
AGBL2	5.00×10^{-4}	4.29×10^{-3}	2.80	1.38×10	7.30

HMGB3	3.38×10^{-9}	1.75×10^{-7}	1.67	1.38×10	4.17
APBB1IP	1.84×10^{-5}	3.13×10^{-4}	1.71	1.38×10	4.06
MNS1	3.88×10^{-3}	2.20×10^{-2}	1.52	1.38×10	6.14
VSTM1	1.70×10^{-7}	5.56×10^{-6}	6.34	1.37×10	1.39×10^{-1}
C1orf228	8.76×10^{-5}	1.09×10^{-3}	1.97	1.37×10	5.77
ERICH3	1.18×10^{-4}	1.39×10^{-3}	3.56	1.36×10	5.36
C11orf21	3.01×10^{-3}	1.80×10^{-2}	1.53	1.36×10	4.72
C3AR1	1.34×10^{-4}	1.53×10^{-3}	1.73	1.35×10	3.96
CCR3	8.69×10^{-9}	4.14×10^{-7}	4.70	1.35×10	3.38×10^{-1}
ARRDC4	1.25×10^{-6}	3.26×10^{-5}	1.00	1.35×10	6.84
SCNN1B	1.43×10^{-5}	2.56×10^{-4}	1.01	1.35×10	6.49
CD38	1.31×10^{-3}	9.24×10^{-3}	1.40	1.35×10	5.49
OSBPL3	1.60×10^{-4}	1.77×10^{-3}	1.17	1.34×10	6.78
BBOF1	2.66×10^{-3}	1.63×10^{-2}	1.38	1.34×10	5.66
PLA1A	1.72×10^{-3}	1.15×10^{-2}	1.59	1.34×10	4.32

STRA6	1.17×10^{-7}	3.97×10^{-6}	1.63	1.33×10	4.78
LAT2	8.23×10^{-5}	1.04×10^{-3}	1.58	1.33×10	4.29
PRR5L	1.09×10^{-14}	1.69×10^{-12}	2.00	1.33×10	3.31
MISP	3.11×10^{-3}	1.85×10^{-2}	1.54	1.32×10	6.41
TMEM154	3.86×10^{-5}	5.80×10^{-4}	1.77	1.32×10	3.49
CCDC170	2.78×10^{-6}	6.51×10^{-5}	3.02	1.32×10	3.73
C9orf135	2.06×10^{-4}	2.16×10^{-3}	4.03	1.32×10	2.82
CD300A	2.51×10^{-4}	2.52×10^{-3}	1.75	1.32×10	3.22
LIF	2.55×10^{-3}	1.58×10^{-2}	1.18	1.32×10	7.94
IGSF6	2.07×10^{-4}	2.17×10^{-3}	1.89	1.31×10	3.13
CCR2	2.91×10^{-3}	1.76×10^{-2}	1.79	1.31×10	4.21
OXTR	5.03×10^{-6}	1.10×10^{-4}	4.06	1.30×10	1.49
KIF19	3.37×10^{-4}	3.13×10^{-3}	1.79	1.30×10	5.87
RAB37	4.30×10^{-5}	6.27×10^{-4}	1.78	1.30×10	4.37
MIAT	3.93×10^{-3}	2.22×10^{-2}	1.32	1.30×10	6.09

FCGR2B	2.40×10^{-3}	1.50×10^{-2}	1.14	1.30×10	6.13
FAM49B	6.58×10^{-6}	1.37×10^{-4}	1.05	1.29×10	5.92
DZIP3	1.48×10^{-3}	1.03×10^{-2}	1.32	1.28×10	5.82
HN1	1.24×10^{-4}	1.45×10^{-3}	1.14	1.28×10	6.02
SLC7A7	1.17×10^{-3}	8.41×10^{-3}	1.06	1.28×10	6.41
ADGRE1	2.41×10^{-8}	1.02×10^{-6}	4.25	1.27×10	4.63×10^{-1}
KCNAB2	1.82×10^{-4}	1.95×10^{-3}	1.20	1.26×10	5.52
PLEKHG7	1.46×10^{-4}	1.64×10^{-3}	2.20	1.26×10	4.11
EDIL3	5.50×10^{-6}	1.17×10^{-4}	1.63	1.26×10	4.11
TTLL10	1.23×10^{-3}	8.81×10^{-3}	2.16	1.26×10	5.03
DPYS	5.86×10^{-32}	1.04×10^{-28}	6.46	1.25×10	1.50×10^{-1}
NRROS	1.90×10^{-3}	1.24×10^{-2}	1.17	1.25×10	5.01
PLBD1	3.20×10^{-4}	3.02×10^{-3}	1.02	1.25×10	6.14
SGPP2	7.14×10^{-10}	4.26×10^{-8}	1.40	1.24×10	4.64
FHAD1	5.14×10^{-5}	7.20×10^{-4}	3.00	1.24×10	4.69

GREM1	$5.46×10^{-6}$	$1.17×10^{-4}$	1.48	1.24×10	6.81
GAPT	$1.96×10^{-8}$	$8.49×10^{-7}$	3.33	1.24×10	1.18
CD3D	$1.82×10^{-4}$	$1.95×10^{-3}$	1.09	1.24×10	5.68
PTPN7	$5.99×10^{-5}$	$8.07×10^{-4}$	1.55	1.23×10	4.35
LILRB1	$1.97×10^{-3}$	$1.28×10^{-2}$	1.66	1.23×10	3.76
ARMC3	$2.06×10^{-4}$	$2.16×10^{-3}$	3.61	1.23×10	3.62
IGSF3	$1.89×10^{-6}$	$4.69×10^{-5}$	1.19	1.22×10	5.51
CCDC113	$9.26×10^{-4}$	$6.95×10^{-3}$	2.18	1.22×10	4.40
DAW1	$3.46×10^{-4}$	$3.20×10^{-3}$	4.04	1.21×10	4.07
CTXN1	$5.36×10^{-3}$	$2.83×10^{-2}$	1.94	1.21×10	4.71
PSTPIP2	$5.02×10^{-4}$	$4.30×10^{-3}$	1.18	1.21×10	4.85
C1orf158	$3.55×10^{-4}$	$3.26×10^{-3}$	4.38	1.20×10	4.08
DZIP1L	$6.16×10^{-3}$	$3.17×10^{-2}$	1.17	1.20×10	7.82
C2orf81	$3.55×10^{-3}$	$2.05×10^{-2}$	1.62	1.20×10	5.09
LYPD6B	$6.40×10^{-4}$	$5.23×10^{-3}$	2.07	1.20×10	4.11

IL18	9.76×10^{-3}	4.50×10^{-2}	1.10	1.20×10	7.45
C21orf58	2.22×10^{-3}	1.41×10^{-2}	1.83	1.18×10	5.00
IL20RB	2.64×10^{-3}	1.62×10^{-2}	1.53	1.17×10	4.60
PKP1	5.02×10^{-3}	2.68×10^{-2}	1.14	1.17×10	6.36
KLRB1	4.02×10^{-5}	5.98×10^{-4}	1.15	1.16×10	5.07
AK7	1.51×10^{-5}	2.67×10^{-4}	2.47	1.16×10	3.68
GPR162	1.56×10^{-3}	1.07×10^{-2}	1.99	1.15×10	4.78
NPL	1.36×10^{-5}	2.47×10^{-4}	2.00	1.15×10	2.92
PIK3R6	3.25×10^{-5}	5.06×10^{-4}	2.39	1.15×10	1.65
IQCD	1.87×10^{-3}	1.23×10^{-2}	1.91	1.15×10	5.30
ANKMY1	6.06×10^{-3}	3.13×10^{-2}	1.14	1.15×10	6.22
CFAP221	8.16×10^{-5}	1.03×10^{-3}	3.06	1.15×10	4.03
NUAK2	1.57×10^{-5}	2.75×10^{-4}	1.16	1.14×10	5.05
LRRC56	5.59×10^{-3}	2.92×10^{-2}	1.10	1.14×10	6.26
CKS2	8.34×10^{-4}	6.43×10^{-3}	1.31	1.14×10	4.14

FGD3	5.92×10^{-5}	8.00×10^{-4}	1.48	1.14×10	3.81
ERICH5	1.49×10^{-4}	1.67×10^{-3}	1.05	1.13×10	6.04
SLAMF6	1.23×10^{-4}	1.43×10^{-3}	1.67	1.13×10	4.13
PIK3R5	7.60×10^{-5}	9.76×10^{-4}	1.40	1.13×10	4.12
ADORA2B	3.78×10^{-5}	5.72×10^{-4}	1.43	1.13×10	4.93
GZMK	9.85×10^{-5}	1.19×10^{-3}	1.28	1.13×10	4.99
CCDC74B	8.48×10^{-4}	6.50×10^{-3}	1.78	1.12×10	4.72
CD19	8.99×10^{-11}	6.59×10^{-9}	2.02	1.12×10	3.08
PLOD2	1.11×10^{-6}	2.93×10^{-5}	1.04	1.12×10	5.43
NPHP1	8.20×10^{-3}	3.92×10^{-2}	1.21	1.11×10	5.82
MET	2.62×10^{-10}	1.69×10^{-8}	1.13	1.11×10	5.05
PRSS33	4.58×10^{-3}	2.49×10^{-2}	1.72	1.11×10	1.87
CCR1	1.11×10^{-4}	1.32×10^{-3}	2.32	1.10×10	1.97
DNAH3	4.16×10^{-4}	3.69×10^{-3}	3.65	1.10×10	4.07
LCK	1.67×10^{-4}	1.82×10^{-3}	1.14	1.09×10	5.33

EMILIN2	8.02×10^{-4}	6.26×10^{-3}	1.11	1.09×10	4.53
C4orf47	6.53×10^{-5}	8.64×10^{-4}	2.32	1.09×10	3.84
RAB44	7.33×10^{-6}	1.49×10^{-4}	2.98	1.09×10	8.96×10^{-1}
IKZF1	1.33×10^{-4}	1.53×10^{-3}	1.40	1.09×10	4.30
TSNAXIP1	1.53×10^{-3}	1.05×10^{-2}	2.01	1.09×10	4.67
TACC3	1.14×10^{-3}	8.24×10^{-3}	1.06	1.08×10	4.93
C11orf97	3.74×10^{-4}	3.38×10^{-3}	3.18	1.08×10	3.46
CCDC96	1.74×10^{-3}	1.16×10^{-2}	1.78	1.08×10	4.21
ANKRD66	3.55×10^{-4}	3.26×10^{-3}	4.51	1.08×10	3.44
TRAF3IP3	1.37×10^{-5}	2.48×10^{-4}	1.60	1.08×10	3.99
MMP25	7.01×10^{-4}	5.63×10^{-3}	2.08	1.08×10	1.95
DOK1	1.39×10^{-6}	3.57×10^{-5}	1.35	1.07×10	4.38
DNAJB13	2.90×10^{-5}	4.63×10^{-4}	2.77	1.07×10	3.73
AKAP14	1.21×10^{-4}	1.41×10^{-3}	3.38	1.07×10	3.87
STK33	7.55×10^{-3}	3.70×10^{-2}	1.41	1.07×10	5.23

IRF5	1.60×10^{-3}	1.09×10^{-2}	1.01	1.06×10	5.38
TRPV2	6.94×10^{-4}	5.60×10^{-3}	1.00	1.06×10	5.06
GRAMD2	9.08×10^{-3}	4.25×10^{-2}	1.34	1.06×10	4.54
CHST11	3.64×10^{-4}	3.32×10^{-3}	1.37	1.06×10	3.79
CD22	5.59×10^{-8}	2.08×10^{-6}	1.61	1.06×10	3.88
SEMA7A	8.51×10^{-4}	6.52×10^{-3}	1.37	1.06×10	3.41
LRRN1	6.02×10^{-7}	1.71×10^{-5}	1.54	1.05×10	3.93
CD300LF	1.41×10^{-7}	4.65×10^{-6}	3.68	1.05×10	6.92×10^{-1}
C1orf87	2.08×10^{-5}	3.50×10^{-4}	4.01	1.05×10	2.69
WDR78	1.56×10^{-4}	1.73×10^{-3}	2.49	1.05×10	3.53
ADGRG5	2.17×10^{-4}	2.25×10^{-3}	1.72	1.04×10	3.10
ZNF474	2.85×10^{-5}	4.56×10^{-4}	3.29	1.03×10	2.96
AOAH	1.81×10^{-4}	1.94×10^{-3}	1.61	1.03×10	3.42
VWA3B	1.27×10^{-4}	1.47×10^{-3}	2.42	1.03×10	5.93
FCER1A	6.81×10^{-3}	3.42×10^{-2}	1.36	1.03×10	3.59

AJUBA	3.31×10^{-4}	3.09×10^{-3}	1.17	1.03×10	4.70
ADGRE3	1.18×10^{-9}	6.73×10^{-8}	3.83	1.02×10	4.72×10^{-1}
FAM65B	6.92×10^{-7}	1.95×10^{-5}	1.42	1.02×10	3.55
ENKUR	1.75×10^{-5}	3.03×10^{-4}	3.88	1.02×10	2.63
FPR3	1.59×10^{-4}	1.76×10^{-3}	1.59	1.01×10	3.03
CXCR2	6.35×10^{-3}	3.25×10^{-2}	2.49	1.01×10	9.01×10^{-1}
C1orf189	1.04×10^{-3}	7.65×10^{-3}	3.52	1.00×10	3.79
TSPAN19	1.51×10^{-3}	1.04×10^{-2}	2.73	1.00×10	4.35
PNCK	9.09×10^{-7}	2.47×10^{-5}	1.99	1.00×10	2.32
CFAP77	2.56×10^{-4}	2.55×10^{-3}	3.93	9.95	1.84
ZBBX	4.70×10^{-4}	4.07×10^{-3}	4.05	9.86	2.76
HVCN1	2.33×10^{-4}	2.38×10^{-3}	1.08	9.85	4.75
CD180	4.11×10^{-5}	6.07×10^{-4}	1.94	9.85	2.68
NHLRC4	8.25×10^{-4}	6.39×10^{-3}	1.87	9.81	3.77
HIST1H2AC	4.83×10^{-4}	4.16×10^{-3}	1.56	9.80	3.24

DRC7	8.96×10^{-4}	6.78×10^{-3}	4.16	9.79	3.62
GZMA	2.11×10^{-3}	1.35×10^{-2}	1.05	9.78	5.04
FAM78A	6.18×10^{-4}	5.10×10^{-3}	1.39	9.77	4.27
ITGB2-AS1	4.37×10^{-5}	6.36×10^{-4}	1.89	9.76	2.79
LGALS12	5.26×10^{-6}	1.14×10^{-4}	3.60	9.74	3.47×10^{-1}
SLC23A1	1.69×10^{-3}	1.14×10^{-2}	1.53	9.72	4.86
TSPAN32	9.40×10^{-3}	4.36×10^{-2}	1.21	9.68	4.16
LRRC25	3.27×10^{-5}	5.08×10^{-4}	2.36	9.67	1.74
RAMP1	4.23×10^{-13}	5.02×10^{-11}	2.47	9.66	1.88
VPREB3	8.84×10^{-9}	4.18×10^{-7}	1.91	9.55	2.83
LMNB1	3.66×10^{-3}	2.10×10^{-2}	1.33	9.53	3.75
MGAT3	5.65×10^{-4}	4.73×10^{-3}	1.27	9.49	4.63
PLD4	6.43×10^{-5}	8.55×10^{-4}	1.57	9.47	2.36
LRRC6	1.89×10^{-4}	2.02×10^{-3}	1.87	9.46	3.49
RASSF6	9.23×10^{-6}	1.82×10^{-4}	1.21	9.44	4.46

C10orf107	6.63×10^{-3}	3.34×10^{-2}	1.94	9.44	4.18
EFCAB12	2.14×10^{-4}	2.23×10^{-3}	2.48	9.43	3.85
CD247	4.87×10^{-5}	6.90×10^{-4}	1.11	9.41	4.35
C6orf118	2.10×10^{-4}	2.20×10^{-3}	4.16	9.41	3.16
CLMP	7.42×10^{-4}	5.90×10^{-3}	1.10	9.37	4.32
TCTEX1D4	5.46×10^{-3}	2.87×10^{-2}	1.87	9.35	4.06
TTLL9	7.33×10^{-4}	5.85×10^{-3}	2.29	9.33	4.70
CCDC173	8.87×10^{-5}	1.10×10^{-3}	2.71	9.33	3.56
OSCAR	7.03×10^{-5}	9.16×10^{-4}	2.72	9.32	1.21
CHL1	4.33×10^{-7}	1.27×10^{-5}	1.32	9.31	3.59
NEK11	6.04×10^{-3}	3.12×10^{-2}	1.46	9.26	4.71
SLC51A	8.58×10^{-4}	6.54×10^{-3}	1.71	9.18	3.74
PTGS1	7.73×10^{-5}	9.88×10^{-4}	1.27	9.06	3.61
TCTEX1D2	1.72×10^{-3}	1.15×10^{-2}	1.70	9.05	3.79
PADI4	1.02×10^{-5}	1.97×10^{-4}	4.24	9.04	2.78×10^{-1}

FGF11	1.99×10^{-6}	4.88×10^{-5}	1.60	9.03	3.45
PLS1	9.54×10^{-6}	1.87×10^{-4}	1.13	9.00	3.89
CLEC4G	1.14×10^{-4}	1.35×10^{-3}	3.30	8.97	4.54×10^{-1}
FDXR	1.89×10^{-3}	1.24×10^{-2}	1.06	8.97	4.86
COX6B2	2.17×10^{-12}	2.16×10^{-10}	2.67	8.94	1.61
LINC01503	7.88×10^{-5}	1.00×10^{-3}	1.78	8.93	3.10
LOC100506844	7.18×10^{-3}	3.56×10^{-2}	1.12	8.92	4.56
MUC16	5.41×10^{-5}	7.50×10^{-4}	1.38	8.91	3.58
ROBO1	1.48×10^{-5}	2.64×10^{-4}	1.00	8.88	4.56
H2AFY2	2.90×10^{-7}	8.94×10^{-6}	1.19	8.83	4.02
CC2D2A	1.07×10^{-2}	4.82×10^{-2}	1.07	8.82	4.62
NUDT10	1.40×10^{-3}	9.77×10^{-3}	2.14	8.81	1.99
COLCA1	1.84×10^{-3}	1.21×10^{-2}	1.99	8.81	2.66
HACD4	1.51×10^{-3}	1.04×10^{-2}	1.40	8.75	4.10
C17orf97	1.55×10^{-3}	1.06×10^{-2}	1.55	8.75	3.31

CFAP206	6.37×10^{-5}	8.48×10^{-4}	3.08	8.71	2.97
CCDC151	5.80×10^{-4}	4.83×10^{-3}	2.97	8.61	3.53
CCL8	1.03×10^{-2}	4.69×10^{-2}	2.08	8.60	2.98
CLEC12A	1.06×10^{-7}	3.66×10^{-6}	3.24	8.59	7.83×10^{-1}
SIGLEC8	7.41×10^{-6}	1.51×10^{-4}	4.04	8.58	4.99×10^{-1}
MS4A2	5.64×10^{-6}	1.19×10^{-4}	1.84	8.57	3.06
KCNJ8	3.71×10^{-4}	3.36×10^{-3}	1.41	8.57	3.38
FCER2	3.62×10^{-6}	8.23×10^{-5}	2.34	8.53	1.53
DTHD1	2.69×10^{-4}	2.65×10^{-3}	2.55	8.49	3.54
CYP2B6	4.91×10^{-4}	4.22×10^{-3}	2.72	8.44	2.01
KCTD17	7.20×10^{-4}	5.77×10^{-3}	1.02	8.42	4.19
RIBC2	6.30×10^{-5}	8.40×10^{-4}	3.03	8.42	2.25
IDO1	3.66×10^{-3}	2.10×10^{-2}	1.15	8.40	3.87
KCNE1	5.67×10^{-5}	7.75×10^{-4}	3.00	8.40	3.07
S1PR4	1.05×10^{-6}	2.79×10^{-5}	2.21	8.39	2.06

SH3BP1	1.67×10^{-4}	1.83×10^{-3}	1.19	8.37	3.79
LYL1	1.46×10^{-6}	3.71×10^{-5}	1.71	8.35	2.77
TREM2	3.60×10^{-4}	3.29×10^{-3}	1.92	8.32	1.80
RIIAD1	5.78×10^{-5}	7.85×10^{-4}	4.34	8.31	2.29
CACNG6	2.55×10^{-4}	2.54×10^{-3}	3.90	8.30	3.48
RASGRP4	1.85×10^{-4}	1.97×10^{-3}	2.34	8.29	1.35
HIST1H2BD	5.59×10^{-5}	7.69×10^{-4}	1.61	8.26	2.50
CCDC33	1.36×10^{-5}	2.48×10^{-4}	3.63	8.20	3.35
PLPPR3	1.10×10^{-6}	2.91×10^{-5}	3.51	8.16	1.89
MGARP	1.25×10^{-4}	1.45×10^{-3}	1.59	8.14	2.76
TOR4A	9.61×10^{-5}	1.17×10^{-3}	1.24	8.08	3.73
C7orf57	7.67×10^{-5}	9.82×10^{-4}	3.24	8.05	3.22
CLEC7A	5.25×10^{-3}	2.78×10^{-2}	1.26	8.04	3.05
CYB5R4	2.07×10^{-4}	2.17×10^{-3}	1.08	8.03	3.53
CFP	5.46×10^{-4}	4.61×10^{-3}	1.95	8.02	2.02

NTF4	9.97×10^{-4}	7.39×10^{-3}	1.05	8.01	4.15
LRRC74B	7.82×10^{-4}	6.15×10^{-3}	3.01	8.00	4.83
ENPEP	9.53×10^{-9}	4.44×10^{-7}	2.10	7.95	1.67
HLA-DQB1	1.19×10^{-5}	2.22×10^{-4}	1.18	7.93	4.09
BST1	2.42×10^{-7}	7.67×10^{-6}	1.38	7.92	3.10
OTX2	1.47×10^{-6}	3.73×10^{-5}	4.22	7.90	4.23×10^{-1}
TTC16	2.18×10^{-4}	2.26×10^{-3}	2.71	7.88	3.97
TCTE1	6.98×10^{-4}	5.62×10^{-3}	2.35	7.85	3.93
GLIS3	2.27×10^{-5}	3.76×10^{-4}	1.05	7.85	3.79
MDH1B	5.56×10^{-4}	4.68×10^{-3}	2.18	7.84	3.35
WNT7B	1.81×10^{-3}	1.20×10^{-2}	1.77	7.82	3.39
LRRC43	1.65×10^{-3}	1.12×10^{-2}	1.79	7.77	3.69
CHN1	1.44×10^{-4}	1.62×10^{-3}	1.15	7.75	3.42
TRERF1	6.09×10^{-4}	5.04×10^{-3}	1.17	7.74	3.48
ARMC4	5.81×10^{-6}	1.23×10^{-4}	3.62	7.73	2.68

RERG	2.52×10^{-3}	1.57×10^{-2}	1.07	7.71	3.73
CFAP99	2.86×10^{-4}	2.77×10^{-3}	3.01	7.68	3.70
RIBC1	8.57×10^{-4}	6.54×10^{-3}	1.99	7.66	2.83
CD84	3.60×10^{-7}	1.08×10^{-5}	2.11	7.66	1.68
GSC	6.80×10^{-5}	8.93×10^{-4}	2.29	7.65	1.47
LAMP3	6.41×10^{-4}	5.23×10^{-3}	1.20	7.64	3.23
KIAA1841	4.75×10^{-5}	6.78×10^{-4}	1.77	7.62	2.34
MORN3	3.99×10^{-3}	2.24×10^{-2}	1.48	7.62	3.33
BMP7	1.10×10^{-2}	4.94×10^{-2}	1.36	7.58	3.92
LOC102724323	5.68×10^{-3}	2.97×10^{-2}	1.57	7.55	2.96
FCRL2	7.33×10^{-5}	9.48×10^{-4}	1.37	7.53	3.74
KIAA2012	2.50×10^{-4}	2.51×10^{-3}	3.95	7.52	3.21
STOX1	3.55×10^{-3}	2.05×10^{-2}	1.14	7.52	3.34
LOC441081	1.23×10^{-3}	8.81×10^{-3}	1.46	7.51	2.84
CD86	6.06×10^{-4}	5.02×10^{-3}	1.44	7.50	2.50

DYNLRB2	1.19×10^{-4}	1.40×10^{-3}	2.90	7.47	2.58
DNAH12	5.31×10^{-5}	7.41×10^{-4}	3.12	7.42	2.40
DYDC2	1.51×10^{-4}	1.68×10^{-3}	4.21	7.42	2.14
HLA-DRA	7.72×10^{-4}	6.09×10^{-3}	1.19	7.42	2.68
TCTEX1D1	5.13×10^{-5}	7.18×10^{-4}	2.16	7.41	2.40
PPP1R14C	5.50×10^{-5}	7.60×10^{-4}	3.02	7.41	2.55
GCLM	1.15×10^{-8}	5.27×10^{-7}	1.05	7.40	3.61
ANKRD44	1.18×10^{-3}	8.46×10^{-3}	1.03	7.39	3.62
CD1C	2.59×10^{-7}	8.13×10^{-6}	2.36	7.38	1.47
EFHB	1.24×10^{-3}	8.86×10^{-3}	3.93	7.38	3.47
C4orf48	1.15×10^{-5}	2.16×10^{-4}	1.31	7.37	2.88
FCGR2C	7.70×10^{-3}	3.75×10^{-2}	1.29	7.35	3.03
WDR63	9.08×10^{-5}	1.12×10^{-3}	3.50	7.35	2.34
KCNJ15	2.58×10^{-4}	2.57×10^{-3}	1.42	7.33	2.46
TP73	4.04×10^{-3}	2.26×10^{-2}	1.18	7.33	4.09

RGS18	1.77×10^{-8}	7.79×10^{-7}	3.20	7.27	6.28×10^{-1}
CD300LB	2.53×10^{-4}	2.53×10^{-3}	2.43	7.25	1.01
CFAP57	1.36×10^{-3}	9.53×10^{-3}	2.61	7.22	2.91
C1orf186	8.10×10^{-5}	1.02×10^{-3}	1.48	7.18	2.82
CCDC103	3.55×10^{-4}	3.26×10^{-3}	2.27	7.16	2.48
PACRG	1.88×10^{-4}	2.01×10^{-3}	2.05	7.16	2.42
SPAG17	2.52×10^{-5}	4.14×10^{-4}	3.36	7.14	2.19
POU2F2	8.17×10^{-4}	6.34×10^{-3}	1.42	7.14	2.88
SDK1	1.41×10^{-6}	3.62×10^{-5}	1.97	7.14	2.46
SOX21	1.05×10^{-7}	3.64×10^{-6}	1.80	7.13	2.21
PADI1	4.00×10^{-4}	3.57×10^{-3}	3.36	7.13	1.02
ADAP2	1.98×10^{-3}	1.29×10^{-2}	1.11	7.10	3.33
IL5RA	1.67×10^{-5}	2.90×10^{-4}	3.32	7.07	1.27
DMRT3	2.60×10^{-4}	2.58×10^{-3}	1.21	7.07	3.15
ABCC3	3.23×10^{-4}	3.04×10^{-3}	1.15	7.07	3.45

PDPN	2.42×10^{-8}	1.03×10^{-6}	1.43	7.06	2.76
CFAP74	1.18×10^{-4}	1.39×10^{-3}	3.75	7.04	3.13
KLHDC7B	1.83×10^{-3}	1.21×10^{-2}	1.59	7.04	2.18
APOBEC4	3.68×10^{-5}	5.58×10^{-4}	3.70	7.00	2.39
ADAM12	1.07×10^{-3}	7.85×10^{-3}	1.82	6.96	1.97
HAVCR2	6.50×10^{-5}	8.61×10^{-4}	1.42	6.95	2.33
CEMIP	5.02×10^{-5}	7.07×10^{-4}	1.87	6.94	2.37
C16orf71	6.70×10^{-4}	5.42×10^{-3}	2.10	6.94	2.74
CASC1	2.82×10^{-4}	2.74×10^{-3}	2.40	6.87	2.62
SLC2A6	8.99×10^{-5}	1.12×10^{-3}	1.41	6.85	2.52
MAP3K19	9.67×10^{-5}	1.18×10^{-3}	3.11	6.84	2.32
ADGRF1	2.74×10^{-5}	4.41×10^{-4}	2.16	6.80	2.15
GJB3	1.04×10^{-2}	4.72×10^{-2}	1.20	6.76	4.54
TMEM156	5.21×10^{-5}	7.28×10^{-4}	1.56	6.76	2.37
PIH1D2	3.43×10^{-4}	3.18×10^{-3}	2.05	6.75	2.50

COLCA2	1.95×10^{-3}	1.27×10^{-2}	1.39	6.73	3.26
LRTOMT	6.68×10^{-3}	3.36×10^{-2}	1.25	6.72	3.04
PPIL6	2.52×10^{-4}	2.53×10^{-3}	2.45	6.72	2.07
IQCA1	1.17×10^{-5}	2.19×10^{-4}	1.79	6.71	2.90
IKZF3	5.97×10^{-5}	8.06×10^{-4}	1.29	6.69	2.95
MMD	9.99×10^{-7}	2.68×10^{-5}	1.27	6.69	2.63
CFAP65	1.16×10^{-4}	1.37×10^{-3}	2.88	6.66	2.74
P2RY10	6.40×10^{-4}	5.23×10^{-3}	1.13	6.66	2.76
CBFA2T3	7.06×10^{-4}	5.67×10^{-3}	1.05	6.65	3.28
C19orf38	6.95×10^{-5}	9.09×10^{-4}	2.14	6.64	1.37
PADI3	3.59×10^{-5}	5.48×10^{-4}	3.56	6.64	9.76×10^{-1}
CDA	6.16×10^{-6}	1.29×10^{-4}	1.82	6.64	1.54
NFE2	2.56×10^{-7}	8.07×10^{-6}	3.86	6.64	3.88×10^{-1}
ADAP1	1.17×10^{-5}	2.18×10^{-4}	1.43	6.62	2.59
AHNAK2	6.28×10^{-5}	8.38×10^{-4}	1.77	6.62	2.62

FCHO1	5.61×10^{-4}	4.71×10^{-3}	1.46	6.61	2.43
LINC01272	4.96×10^{-4}	4.27×10^{-3}	1.73	6.61	1.49
CLEC4A	6.31×10^{-4}	5.18×10^{-3}	1.24	6.57	2.71
CATIP	7.67×10^{-4}	6.07×10^{-3}	2.99	6.56	3.23
SLC5A3	5.63×10^{-8}	2.10×10^{-6}	1.29	6.55	2.77
TEKT4	1.07×10^{-4}	1.28×10^{-3}	3.40	6.54	3.02
PLXNC1	1.08×10^{-4}	1.29×10^{-3}	1.47	6.51	2.12
P2RY13	1.72×10^{-3}	1.15×10^{-2}	2.50	6.50	1.31
C11orf70	1.45×10^{-4}	1.63×10^{-3}	2.30	6.49	1.98
DUSP18	1.65×10^{-3}	1.12×10^{-2}	1.06	6.48	3.31
LILRB3	4.44×10^{-3}	2.43×10^{-2}	1.64	6.46	1.54
FAP	1.88×10^{-11}	1.57×10^{-9}	1.67	6.45	1.98
CFAP46	3.31×10^{-4}	3.09×10^{-3}	3.15	6.39	3.14
NFAM1	1.62×10^{-4}	1.78×10^{-3}	2.01	6.33	1.35
TMEM37	2.57×10^{-4}	2.56×10^{-3}	1.49	6.31	2.69

EDARADD	$6.03×10^{-7}$	$1.71×10^{-5}$	1.71	6.23	2.19
DNAH5	$7.28×10^{-5}$	$9.44×10^{-4}$	3.01	6.16	2.78
SP140	$1.85×10^{-3}$	$1.22×10^{-2}$	1.14	6.14	2.92
PSPH	$3.93×10^{-6}$	$8.85×10^{-5}$	1.11	6.10	2.95
TTC29	$5.34×10^{-6}$	$1.15×10^{-4}$	3.25	6.10	1.56
TSGA10	$8.96×10^{-4}$	$6.78×10^{-3}$	1.44	6.04	2.59
DNAH9	$7.90×10^{-5}$	$1.00×10^{-3}$	3.94	6.04	2.79
IL18R1	$2.87×10^{-4}$	$2.78×10^{-3}$	1.04	5.96	2.85
SPNS3	$4.97×10^{-3}$	$2.66×10^{-2}$	1.44	5.96	1.60
CCDC39	$1.41×10^{-3}$	$9.82×10^{-3}$	1.64	5.95	2.83
PCSK6	$2.27×10^{-3}$	$1.43×10^{-2}$	1.34	5.95	1.95
GAB3	$5.00×10^{-4}$	$4.29×10^{-3}$	1.12	5.94	2.90
TNFAIP8L2	$9.19×10^{-6}$	$1.81×10^{-4}$	2.39	5.94	1.40
GPR132	$7.78×10^{-3}$	$3.77×10^{-2}$	1.19	5.94	2.31
PTPN22	$3.45×10^{-3}$	$2.01×10^{-2}$	1.21	5.93	2.37

EPPIN	1.07×10^{-5}	2.03×10^{-4}	2.76	5.89	3.09
NEK10	2.43×10^{-4}	2.46×10^{-3}	2.67	5.89	2.22
ZNF671	1.96×10^{-5}	3.30×10^{-4}	1.18	5.88	2.89
MYB	9.67×10^{-5}	1.18×10^{-3}	1.29	5.85	2.94
GJA5	1.62×10^{-12}	1.66×10^{-10}	1.84	5.85	1.49
AK8	2.86×10^{-3}	1.74×10^{-2}	1.66	5.82	2.22
NLRC3	1.02×10^{-3}	7.52×10^{-3}	1.11	5.82	3.09
SKAP2	5.31×10^{-3}	2.81×10^{-2}	1.31	5.81	2.22
RNASE2	1.53×10^{-8}	6.88×10^{-7}	3.54	5.80	4.79×10^{-1}
PRKCB	1.57×10^{-9}	8.61×10^{-8}	1.93	5.77	1.58
CMKLR1	2.60×10^{-3}	1.60×10^{-2}	1.17	5.75	2.40
ADRA2C	7.20×10^{-6}	1.47×10^{-4}	1.51	5.69	1.74
CNGA4	2.95×10^{-4}	2.83×10^{-3}	3.16	5.68	2.08
HYDIN	6.54×10^{-4}	5.32×10^{-3}	2.26	5.68	2.47
ERICH6-AS1	1.45×10^{-3}	1.00×10^{-2}	1.77	5.66	2.89

KNDC1	2.82×10^{-4}	2.74×10^{-3}	2.73	5.65	2.47
PCSK5	7.93×10^{-12}	7.07×10^{-10}	1.56	5.65	1.96
DPEP1	5.89×10^{-3}	3.06×10^{-2}	1.36	5.64	1.85
ZMYND12	2.94×10^{-5}	4.67×10^{-4}	2.15	5.61	1.63
MIR4435-2HG	6.21×10^{-3}	3.19×10^{-2}	1.09	5.60	2.71
SLAMF8	2.80×10^{-4}	2.73×10^{-3}	1.40	5.60	2.28
RASSF9	3.89×10^{-3}	2.20×10^{-2}	1.17	5.60	2.65
SPN	1.80×10^{-4}	1.94×10^{-3}	1.49	5.59	2.04
DMRT2	7.68×10^{-4}	6.07×10^{-3}	1.17	5.58	2.82
CDC20B	7.41×10^{-3}	3.64×10^{-2}	2.44	5.57	1.50
LILRB4	3.59×10^{-4}	3.29×10^{-3}	1.72	5.57	1.37
MAP1LC3C	2.11×10^{-3}	1.35×10^{-2}	1.55	5.56	1.67
GSDMC	8.47×10^{-3}	4.02×10^{-2}	1.80	5.55	3.36
CD33	1.07×10^{-3}	7.84×10^{-3}	1.69	5.55	1.80
CARD9	1.85×10^{-4}	1.97×10^{-3}	1.87	5.52	1.41

CYSLTR1	1.17×10^{-5}	2.19×10^{-4}	1.61	5.50	1.72
KCNJ2	1.41×10^{-12}	1.48×10^{-10}	1.97	5.47	1.43
WNT4	8.27×10^{-5}	1.04×10^{-3}	1.90	5.47	1.78
CFAP161	4.97×10^{-5}	7.02×10^{-4}	2.60	5.47	1.77
EVI2A	4.73×10^{-4}	4.09×10^{-3}	1.18	5.45	2.53
NTM	1.91×10^{-3}	1.25×10^{-2}	1.17	5.44	2.10
SIRPB2	7.50×10^{-5}	9.64×10^{-4}	3.05	5.42	6.84×10^{-1}
PZP	1.11×10^{-3}	8.02×10^{-3}	2.74	5.38	2.07
TTC26	1.83×10^{-3}	1.21×10^{-2}	1.05	5.38	2.79
NAT1	5.56×10^{-3}	2.91×10^{-2}	1.27	5.37	2.60
EFHC2	3.86×10^{-4}	3.46×10^{-3}	2.28	5.34	1.66
NME9	1.06×10^{-2}	4.81×10^{-2}	1.53	5.32	2.71
FAM229B	6.80×10^{-3}	3.41×10^{-2}	1.12	5.31	2.62
GPR87	3.90×10^{-3}	2.20×10^{-2}	1.07	5.30	3.05
PTTG1	4.18×10^{-4}	3.70×10^{-3}	1.36	5.30	1.84

LINC01267	$2.45×10^{-5}$	$4.03×10^{-4}$	2.52	5.29	2.26
DNAH2	$4.63×10^{-5}$	$6.64×10^{-4}$	3.41	5.29	2.51
WDFY4	$7.17×10^{-6}$	$1.47×10^{-4}$	1.45	5.28	1.95
NUSAP1	$3.99×10^{-3}$	$2.24×10^{-2}$	1.51	5.28	1.60
DNAH10	$2.86×10^{-4}$	$2.77×10^{-3}$	2.48	5.28	2.29
HRH1	$4.24×10^{-3}$	$2.35×10^{-2}$	1.04	5.26	2.32
FNDC1	$6.31×10^{-3}$	$3.23×10^{-2}$	2.13	5.24	1.01
LOC730202	$8.69×10^{-3}$	$4.10×10^{-2}$	1.77	5.21	2.55
KLK10	$8.57×10^{-4}$	$6.54×10^{-3}$	1.52	5.19	1.50
GRIN3B	$3.87×10^{-3}$	$2.19×10^{-2}$	1.54	5.18	2.48
SPATA17	$5.02×10^{-4}$	$4.30×10^{-3}$	2.54	5.18	1.75
CEBPE	$1.28×10^{-13}$	$1.63×10^{-11}$	4.46	5.14	$1.53×10^{-1}$
HLA-DPB1	$1.67×10^{-6}$	$4.17×10^{-5}$	1.01	5.13	2.57
RSPH14	$1.88×10^{-4}$	$2.01×10^{-3}$	2.64	5.12	1.59
EFNB3	$5.37×10^{-5}$	$7.46×10^{-4}$	1.62	5.10	2.01

PIK3CG	3.96×10^{-5}	5.91×10^{-4}	1.32	5.10	1.96
EFCAB10	5.01×10^{-4}	4.29×10^{-3}	2.56	5.09	1.58
FBXO36	7.14×10^{-3}	3.54×10^{-2}	1.17	5.07	2.47
FZD2	4.55×10^{-9}	2.28×10^{-7}	2.09	5.07	1.41
ANKUB1	6.58×10^{-5}	8.69×10^{-4}	4.93	5.05	1.36
GCNT1	3.54×10^{-7}	1.06×10^{-5}	1.06	5.04	2.42
TRIP13	7.79×10^{-5}	9.95×10^{-4}	2.24	5.02	1.68
SIT1	1.37×10^{-4}	1.57×10^{-3}	1.44	5.01	2.17
CEP19	8.95×10^{-5}	1.11×10^{-3}	1.35	5.00	2.13
PTGDR2	9.74×10^{-6}	1.90×10^{-4}	3.13	5.00	3.96×10^{-1}
MMP9	4.32×10^{-5}	6.30×10^{-4}	2.23	4.97	9.94×10^{-1}
ECEL1	1.68×10^{-3}	1.13×10^{-2}	1.57	4.96	1.25
C4orf22	5.43×10^{-4}	4.59×10^{-3}	3.57	4.95	1.32
CCDC81	2.70×10^{-4}	2.65×10^{-3}	1.36	4.95	2.32
LY86	2.83×10^{-4}	2.75×10^{-3}	1.37	4.94	1.67

ECT2L	1.58×10^{-3}	1.08×10^{-2}	2.01	4.90	1.99
TIGIT	3.02×10^{-4}	2.88×10^{-3}	1.25	4.86	2.16
LRRC18	5.72×10^{-5}	7.79×10^{-4}	3.16	4.86	1.81
FBXO15	6.23×10^{-4}	5.12×10^{-3}	2.45	4.85	1.83
GATA1	1.48×10^{-5}	2.64×10^{-4}	3.22	4.85	3.34×10^{-1}
ADRBK2	6.90×10^{-6}	1.43×10^{-4}	1.16	4.81	2.08
TMEM232	6.75×10^{-5}	8.88×10^{-4}	2.46	4.81	1.29
DUOX2	1.98×10^{-3}	1.29×10^{-2}	1.77	4.77	2.39
SNX20	1.09×10^{-4}	1.30×10^{-3}	1.94	4.77	1.29
TNNI3	3.54×10^{-4}	3.25×10^{-3}	3.30	4.76	2.58
CTLA4	5.25×10^{-5}	7.32×10^{-4}	1.28	4.74	1.95
CR1	1.07×10^{-6}	2.84×10^{-5}	3.23	4.73	4.03×10^{-1}
DGAT2	2.16×10^{-3}	1.37×10^{-2}	1.43	4.72	1.43
SIRPB1	5.81×10^{-4}	4.84×10^{-3}	2.26	4.72	6.49×10^{-1}
FMO6P	2.72×10^{-9}	1.42×10^{-7}	1.98	4.70	1.28

CEP72	1.26×10^{-4}	1.46×10^{-3}	1.01	4.66	2.31
NTRK1	1.56×10^{-6}	3.95×10^{-5}	2.98	4.65	6.20×10^{-1}
FER1L6	1.43×10^{-4}	1.62×10^{-3}	2.99	4.63	8.84×10^{-1}
FCRLA	7.83×10^{-4}	6.15×10^{-3}	1.15	4.62	2.80
SAMD15	3.68×10^{-5}	5.58×10^{-4}	3.12	4.61	1.12
CFAP58	5.40×10^{-4}	4.58×10^{-3}	2.43	4.61	1.91
ZC3HAV1L	1.02×10^{-2}	4.66×10^{-2}	1.07	4.57	2.22
CD1D	4.83×10^{-7}	1.41×10^{-5}	2.09	4.57	1.12
PAPPA	2.63×10^{-9}	1.38×10^{-7}	2.75	4.56	5.20×10^{-1}
CD244	1.15×10^{-3}	8.28×10^{-3}	1.70	4.56	1.30
SPATS1	9.73×10^{-5}	1.18×10^{-3}	4.08	4.54	1.12
KCNH3	1.55×10^{-3}	1.06×10^{-2}	2.18	4.54	2.07
FAM26F	1.52×10^{-6}	3.86×10^{-5}	1.54	4.53	1.49
ANKK1	2.40×10^{-3}	1.50×10^{-2}	1.16	4.53	2.28
IL2RA	5.76×10^{-4}	4.81×10^{-3}	1.85	4.52	1.32

BARX1	2.36×10^{-28}	2.53×10^{-25}	5.96	4.51	8.05×10^{-2}
TOX2	8.00×10^{-5}	1.01×10^{-3}	1.37	4.49	1.74
TTC30A	6.50×10^{-3}	3.30×10^{-2}	1.17	4.48	1.60
LRRIQ1	5.57×10^{-4}	4.68×10^{-3}	2.61	4.48	1.53
P2RY2	4.45×10^{-8}	1.71×10^{-6}	1.45	4.47	1.61
WDR49	7.67×10^{-5}	9.82×10^{-4}	2.80	4.46	1.43
KCNRG	2.87×10^{-4}	2.77×10^{-3}	2.16	4.43	1.94
PRAM1	4.31×10^{-4}	3.80×10^{-3}	1.36	4.42	1.76
SRD5A2	1.04×10^{-4}	1.25×10^{-3}	5.27	4.39	1.07
ITGB6	2.03×10^{-6}	4.97×10^{-5}	1.40	4.38	1.59
UBE2T	4.92×10^{-3}	2.65×10^{-2}	1.67	4.34	1.59
GAL3ST2	4.23×10^{-5}	6.22×10^{-4}	1.59	4.32	2.00
HOTAIRM1	3.64×10^{-3}	2.09×10^{-2}	1.78	4.32	1.87
LRRC34	2.58×10^{-3}	1.59×10^{-2}	1.64	4.29	1.72
ANKDD1B	2.17×10^{-4}	2.25×10^{-3}	2.05	4.28	2.07

HPGDS	1.54×10^{-5}	2.71×10^{-4}	1.74	4.27	1.25
HS3ST6	5.34×10^{-3}	2.82×10^{-2}	2.44	4.27	2.14
TUBB3	2.98×10^{-3}	1.79×10^{-2}	1.85	4.26	1.42
ENOX1	3.10×10^{-4}	2.94×10^{-3}	1.79	4.25	1.21
CYTL1	8.22×10^{-8}	2.94×10^{-6}	2.61	4.24	8.17×10^{-1}
SCNN1G	9.40×10^{-7}	2.54×10^{-5}	2.27	4.24	9.76×10^{-1}
CCR4	1.82×10^{-5}	3.10×10^{-4}	1.69	4.24	1.40
GPR34	6.87×10^{-3}	3.44×10^{-2}	1.42	4.20	1.92
SNAI3	8.16×10^{-4}	6.34×10^{-3}	1.44	4.18	1.81
RFX3	1.25×10^{-3}	8.90×10^{-3}	1.23	4.18	2.08
GALNT14	1.17×10^{-7}	3.97×10^{-6}	1.67	4.17	1.27
SIGLEC9	9.13×10^{-5}	1.13×10^{-3}	2.13	4.14	7.87×10^{-1}
GPR65	7.36×10^{-4}	5.87×10^{-3}	1.60	4.11	1.16
SERPINB10	6.26×10^{-4}	5.15×10^{-3}	3.35	4.10	4.26×10^{-1}
FAM227A	1.78×10^{-4}	1.92×10^{-3}	2.44	4.08	1.36

HIST3H2A	7.58×10^{-3}	3.70×10^{-2}	1.31	4.08	1.69
ADORA3	4.38×10^{-5}	6.36×10^{-4}	2.98	4.06	3.83×10^{-1}
ASCL3	1.46×10^{-10}	1.01×10^{-8}	3.71	4.04	5.62×10^{-1}
SAMD3	8.38×10^{-6}	1.68×10^{-4}	1.30	4.01	1.50
SAMD9	3.79×10^{-4}	3.42×10^{-3}	1.04	4.01	1.89
SIGLEC17P	3.31×10^{-6}	7.62×10^{-5}	1.85	3.98	1.38
P2RY6	8.82×10^{-6}	1.76×10^{-4}	1.76	3.96	1.14
PFKFB4	9.20×10^{-4}	6.92×10^{-3}	1.20	3.96	1.64
CCDC89	1.11×10^{-3}	8.04×10^{-3}	2.68	3.96	1.15
HLA-DQB1-AS1	1.79×10^{-3}	1.19×10^{-2}	1.10	3.92	1.89
CNFN	5.65×10^{-5}	7.74×10^{-4}	1.33	3.91	1.48
TPSD1	1.29×10^{-3}	9.13×10^{-3}	1.33	3.89	1.64
TREML2	1.22×10^{-7}	4.10×10^{-6}	3.00	3.86	4.12×10^{-1}
PCED1B-AS1	3.18×10^{-3}	1.88×10^{-2}	1.37	3.85	1.70
TMCC2	8.93×10^{-4}	6.77×10^{-3}	1.05	3.83	1.87

ZC2HC1C	8.68×10^{-3}	4.10×10^{-2}	1.12	3.82	1.76
PLA2G10	6.46×10^{-3}	3.29×10^{-2}	1.32	3.81	2.06
CCNB1	5.94×10^{-3}	3.08×10^{-2}	1.06	3.80	1.73
HAP1	1.77×10^{-7}	5.75×10^{-6}	2.21	3.79	8.78×10^{-1}
IRX6	4.80×10^{-5}	6.82×10^{-4}	2.75	3.78	9.12×10^{-1}
CD36	5.09×10^{-3}	2.71×10^{-2}	1.39	3.77	1.43
SOX21-AS1	9.02×10^{-7}	2.46×10^{-5}	1.22	3.76	1.64
SIGLEC6	9.33×10^{-7}	2.53×10^{-5}	2.22	3.75	9.93×10^{-1}
SYT17	1.11×10^{-3}	8.07×10^{-3}	1.10	3.75	2.03
HOXD9	4.67×10^{-7}	1.36×10^{-5}	1.91	3.75	1.10
ANXA8L1	1.37×10^{-6}	3.53×10^{-5}	1.83	3.72	1.30
ADGB	1.70×10^{-4}	1.85×10^{-3}	3.45	3.70	1.51
LINC01410	4.17×10^{-3}	2.32×10^{-2}	1.83	3.68	1.37
SLC6A20	1.09×10^{-4}	1.30×10^{-3}	1.38	3.67	1.35
ANKRD45	7.33×10^{-5}	9.48×10^{-4}	2.29	3.66	1.37

ADAMTSL5	1.28×10^{-3}	9.08×10^{-3}	1.20	3.66	1.83
PLA2G7	7.66×10^{-7}	2.13×10^{-5}	2.39	3.65	6.15×10^{-1}
AMN	3.37×10^{-3}	1.97×10^{-2}	1.42	3.65	1.74
FAM129C	1.17×10^{-6}	3.07×10^{-5}	1.69	3.64	1.32
RASGRP1	8.00×10^{-5}	1.01×10^{-3}	1.19	3.63	1.55
NR2E1	9.22×10^{-7}	2.50×10^{-5}	3.17	3.59	3.18×10^{-1}
EGFL6	2.35×10^{-4}	2.39×10^{-3}	1.96	3.58	8.63×10^{-1}
CFAP61	5.68×10^{-5}	7.75×10^{-4}	3.90	3.57	1.20
CD3G	1.93×10^{-3}	1.26×10^{-2}	1.04	3.56	1.88
CEACAM21	6.58×10^{-4}	5.34×10^{-3}	1.51	3.55	1.42
ST8SIA4	3.65×10^{-3}	2.10×10^{-2}	1.03	3.54	1.57
C20orf195	2.86×10^{-3}	1.73×10^{-2}	1.67	3.52	1.40
FCRL1	1.92×10^{-6}	4.74×10^{-5}	2.04	3.51	1.08
PROCA1	6.28×10^{-3}	3.22×10^{-2}	1.01	3.48	1.99
HIGD1B	2.27×10^{-4}	2.33×10^{-3}	1.33	3.48	1.29

LILRA1	1.74×10^{-3}	1.16×10^{-2}	2.10	3.47	6.46×10^{-1}
IL12RB1	5.98×10^{-4}	4.96×10^{-3}	1.15	3.47	1.59
CD1E	1.30×10^{-4}	1.50×10^{-3}	2.16	3.44	6.80×10^{-1}
RGS22	3.91×10^{-5}	5.85×10^{-4}	2.93	3.43	7.98×10^{-1}
MAPK10	1.81×10^{-3}	1.20×10^{-2}	1.43	3.43	1.89
PDE6B	4.54×10^{-5}	6.54×10^{-4}	2.06	3.42	1.26
PLPP4	5.29×10^{-8}	1.99×10^{-6}	3.38	3.42	4.29×10^{-1}
ERMN	2.81×10^{-3}	1.71×10^{-2}	1.44	3.41	1.21
LOC102723354	2.29×10^{-4}	2.34×10^{-3}	1.27	3.41	1.67
FAM161A	4.63×10^{-4}	4.02×10^{-3}	1.01	3.39	1.79
CD101	3.68×10^{-3}	2.11×10^{-2}	1.33	3.38	1.05
MAT1A	3.31×10^{-4}	3.09×10^{-3}	2.26	3.36	1.37
SCIMP	1.89×10^{-6}	4.69×10^{-5}	2.14	3.35	8.75×10^{-1}
C2orf54	5.53×10^{-3}	2.90×10^{-2}	2.03	3.32	1.86
ABHD12B	9.49×10^{-5}	1.16×10^{-3}	2.18	3.31	1.42

SLC6A1	7.90×10^{-3}	3.81×10^{-2}	1.37	3.31	1.12
SIRPG	2.46×10^{-4}	2.48×10^{-3}	1.51	3.29	1.32
CASS4	4.39×10^{-3}	2.41×10^{-2}	1.72	3.28	8.13×10^{-1}
CYP4F3	3.46×10^{-4}	3.20×10^{-3}	2.31	3.28	9.94×10^{-1}
MARCH10	1.64×10^{-4}	1.80×10^{-3}	3.28	3.26	1.10
CLIC3	2.20×10^{-3}	1.40×10^{-2}	1.23	3.25	1.24
EFCAB6	2.88×10^{-3}	1.74×10^{-2}	1.58	3.22	1.21
SLC52A1	4.25×10^{-3}	2.36×10^{-2}	1.63	3.21	1.88
DOC2A	1.33×10^{-4}	1.53×10^{-3}	2.94	3.19	6.13×10^{-1}
ICAM5	1.21×10^{-7}	4.08×10^{-6}	2.02	3.18	8.51×10^{-1}
LPAR5	3.08×10^{-5}	4.85×10^{-4}	1.09	3.18	1.55
ATP8B4	1.71×10^{-3}	1.15×10^{-2}	1.17	3.17	1.28
P2RX5	6.68×10^{-6}	1.39×10^{-4}	1.34	3.17	1.33
GJB4	1.09×10^{-3}	7.94×10^{-3}	1.62	3.16	1.50
SAMD5	8.16×10^{-4}	6.34×10^{-3}	1.47	3.14	9.93×10^{-1}

SERPINI2	1.48×10^{-4}	1.66×10^{-3}	3.00	3.14	1.10
TMEM130	5.28×10^{-7}	1.52×10^{-5}	1.39	3.13	1.19
ALS2CR12	2.33×10^{-3}	1.46×10^{-2}	2.49	3.12	1.20
TESPA1	4.20×10^{-7}	1.24×10^{-5}	1.87	3.10	9.77×10^{-1}
C16orf46	5.38×10^{-3}	2.83×10^{-2}	1.16	3.08	1.55
NPM2	4.29×10^{-5}	6.27×10^{-4}	1.30	3.08	1.41
TMEM40	3.21×10^{-3}	1.89×10^{-2}	2.98	3.06	1.03
DNAH11	4.61×10^{-5}	6.61×10^{-4}	3.47	3.05	8.75×10^{-1}
S1PR5	1.43×10^{-6}	3.66×10^{-5}	1.59	3.05	1.08
LCA5L	7.63×10^{-3}	3.72×10^{-2}	1.33	2.99	1.47
SNX10	2.95×10^{-5}	4.68×10^{-4}	1.68	2.97	9.12×10^{-1}
TMEM150B	2.23×10^{-4}	2.29×10^{-3}	1.99	2.96	8.32×10^{-1}
LOC729683	2.87×10^{-4}	2.77×10^{-3}	1.28	2.96	1.19
CD28	5.93×10^{-4}	4.93×10^{-3}	1.28	2.95	1.21
CMTM1	3.16×10^{-4}	2.98×10^{-3}	1.54	2.94	1.03

FCRL3	1.36×10^{-5}	2.48×10^{-4}	1.66	2.93	1.16
C2orf50	1.30×10^{-4}	1.50×10^{-3}	2.60	2.92	9.94×10^{-1}
AIM2	8.00×10^{-3}	3.84×10^{-2}	1.08	2.90	1.54
DNAH7	7.32×10^{-3}	3.61×10^{-2}	1.54	2.88	1.40
FOXP3	1.55×10^{-5}	2.72×10^{-4}	1.51	2.86	9.77×10^{-1}
UBASH3A	3.20×10^{-4}	3.01×10^{-3}	1.15	2.84	1.42
LRP2	3.96×10^{-3}	2.23×10^{-2}	1.65	2.84	7.89×10^{-1}
AURKA	2.28×10^{-3}	1.44×10^{-2}	1.18	2.83	1.07
SLC4A4	7.73×10^{-4}	6.10×10^{-3}	1.58	2.82	7.57×10^{-1}
PIH1D3	2.18×10^{-4}	2.26×10^{-3}	4.07	2.82	9.18×10^{-1}
CKLF	7.51×10^{-4}	5.97×10^{-3}	1.58	2.82	1.06
SPIB	1.19×10^{-12}	1.27×10^{-10}	1.72	2.81	8.50×10^{-1}
DNAH6	8.00×10^{-5}	1.01×10^{-3}	2.45	2.80	1.12
ALOX15P1	1.17×10^{-4}	1.37×10^{-3}	2.03	2.79	7.76×10^{-1}
CNR2	3.78×10^{-8}	1.51×10^{-6}	2.45	2.79	5.92×10^{-1}

SIGLEC7	5.90×10^{-5}	7.98×10^{-4}	2.67	2.78	4.06×10^{-1}
ADGRB2	1.28×10^{-3}	9.09×10^{-3}	1.61	2.75	1.07
CEACAM4	3.24×10^{-3}	1.91×10^{-2}	2.04	2.75	5.08×10^{-1}
KIF24	7.41×10^{-4}	5.89×10^{-3}	1.87	2.75	1.16
IP6K3	7.98×10^{-6}	1.61×10^{-4}	1.76	2.74	8.65×10^{-1}
FLJ26850	1.10×10^{-2}	4.93×10^{-2}	2.32	2.74	1.07
CNIH2	7.20×10^{-3}	3.57×10^{-2}	1.39	2.73	1.04
TLR10	6.54×10^{-5}	8.65×10^{-4}	1.87	2.72	9.81×10^{-1}
LOC339260	2.04×10^{-3}	1.31×10^{-2}	1.51	2.71	1.19
CLDN9	6.70×10^{-4}	5.42×10^{-3}	1.80	2.69	7.95×10^{-1}
ULK4	4.43×10^{-3}	2.43×10^{-2}	1.15	2.68	1.28
MMP12	4.20×10^{-5}	6.18×10^{-4}	2.76	2.68	2.87×10^{-1}
CCL22	2.82×10^{-4}	2.74×10^{-3}	1.58	2.67	9.12×10^{-1}
CYSLTR2	3.21×10^{-4}	3.02×10^{-3}	2.52	2.65	4.88×10^{-1}
PLB1	1.42×10^{-3}	9.83×10^{-3}	1.25	2.65	1.09

LGR5	2.96×10^{-3}	1.78×10^{-2}	1.86	2.65	2.96×10^{-1}
SIGLEC12	1.06×10^{-4}	1.28×10^{-3}	2.66	2.64	3.00×10^{-1}
TP53AIP1	9.23×10^{-8}	3.27×10^{-6}	1.46	2.63	1.01
OLIG1	1.33×10^{-5}	2.42×10^{-4}	2.43	2.62	3.38×10^{-1}
ALPK2	1.49×10^{-12}	1.54×10^{-10}	3.65	2.61	1.84×10^{-1}
TIAM1	8.35×10^{-6}	1.67×10^{-4}	1.44	2.59	9.28×10^{-1}
PRR18	5.04×10^{-4}	4.31×10^{-3}	2.45	2.58	8.33×10^{-1}
GPSM2	3.03×10^{-5}	4.79×10^{-4}	1.11	2.54	1.21
PPP1R42	5.24×10^{-4}	4.46×10^{-3}	3.24	2.54	8.82×10^{-1}
FRMPD2	8.66×10^{-4}	6.60×10^{-3}	2.74	2.54	1.52
UNC5B-AS1	3.87×10^{-4}	3.47×10^{-3}	1.83	2.53	9.08×10^{-1}
EOMES	7.83×10^{-4}	6.15×10^{-3}	1.17	2.52	1.32
ADAMTS14	4.57×10^{-4}	3.99×10^{-3}	2.53	2.52	8.39×10^{-1}
NLRP12	1.24×10^{-4}	1.45×10^{-3}	3.55	2.49	1.99×10^{-1}
CDK1	9.26×10^{-3}	4.31×10^{-2}	1.66	2.49	8.39×10^{-1}

LINC00861	8.64×10^{-4}	6.59×10^{-3}	1.80	2.49	1.04
ZNF154	1.66×10^{-4}	1.82×10^{-3}	1.24	2.48	1.06
RPGRIP1L	7.34×10^{-3}	3.62×10^{-2}	1.24	2.48	1.16
THRIL	1.65×10^{-3}	1.12×10^{-2}	1.41	2.46	9.69×10^{-1}
LOC101927482	4.06×10^{-5}	6.02×10^{-4}	2.32	2.46	4.69×10^{-1}
FERMT1	1.55×10^{-5}	2.72×10^{-4}	1.10	2.46	1.22
CXCR5	7.62×10^{-13}	8.60×10^{-11}	1.75	2.44	7.19×10^{-1}
ACSBG1	7.66×10^{-3}	3.73×10^{-2}	1.57	2.43	1.28
TENM4	2.63×10^{-4}	2.60×10^{-3}	1.83	2.43	1.00
BCL2L15	6.38×10^{-4}	5.21×10^{-3}	1.70	2.43	7.30×10^{-1}
SHISA8	3.68×10^{-8}	1.47×10^{-6}	2.32	2.42	4.59×10^{-1}
SEC14L4	3.12×10^{-4}	2.96×10^{-3}	3.10	2.41	9.26×10^{-1}
PHOSPHO1	4.74×10^{-3}	2.57×10^{-2}	1.48	2.41	6.37×10^{-1}
ANLN	3.60×10^{-3}	2.08×10^{-2}	2.02	2.40	5.05×10^{-1}
IQUB	4.55×10^{-5}	6.55×10^{-4}	2.76	2.39	6.82×10^{-1}

INSRR	2.37×10^{-3}	1.49×10^{-2}	2.08	2.38	1.11
IL10	3.00×10^{-5}	4.74×10^{-4}	1.93	2.37	5.07×10^{-1}
PIEZO2	1.09×10^{-2}	4.93×10^{-2}	1.52	2.37	1.03
LINC00528	1.03×10^{-3}	7.59×10^{-3}	1.30	2.36	1.08
NEK5	3.59×10^{-4}	3.29×10^{-3}	2.11	2.36	9.35×10^{-1}
FAM124B	2.99×10^{-4}	2.85×10^{-3}	1.17	2.34	9.51×10^{-1}
B3GNT8	3.85×10^{-6}	8.70×10^{-5}	1.56	2.34	7.88×10^{-1}
DCDC5	9.87×10^{-4}	7.32×10^{-3}	2.61	2.34	8.63×10^{-1}
PLA2G2D	2.82×10^{-7}	8.71×10^{-6}	1.54	2.32	7.29×10^{-1}
CD207	4.44×10^{-4}	3.89×10^{-3}	1.35	2.31	8.97×10^{-1}
LINC00426	3.64×10^{-3}	2.09×10^{-2}	1.03	2.30	1.37
CLCNKB	9.75×10^{-5}	1.19×10^{-3}	1.52	2.29	9.32×10^{-1}
BMPR1B	7.77×10^{-8}	2.81×10^{-6}	1.43	2.29	8.08×10^{-1}
EPHX3	1.31×10^{-4}	1.51×10^{-3}	1.64	2.27	6.64×10^{-1}
CCNA2	2.10×10^{-3}	1.35×10^{-2}	1.11	2.24	1.04

HES2	8.84×10^{-5}	1.10×10^{-3}	1.71	2.22	9.22×10^{-1}
CR2	6.16×10^{-3}	3.17×10^{-2}	1.27	2.22	6.61×10^{-1}
CENPM	1.30×10^{-3}	9.17×10^{-3}	1.71	2.21	7.62×10^{-1}
SPATA4	3.36×10^{-4}	3.13×10^{-3}	3.11	2.20	6.99×10^{-1}
MS4A14	7.64×10^{-3}	3.73×10^{-2}	1.24	2.20	9.83×10^{-1}
BLACAT1	2.82×10^{-5}	4.52×10^{-4}	1.15	2.20	9.69×10^{-1}
FLJ41200	4.41×10^{-8}	1.70×10^{-6}	2.14	2.20	5.44×10^{-1}
LINC01504	4.86×10^{-5}	6.89×10^{-4}	1.44	2.18	9.98×10^{-1}
TNFSF8	1.41×10^{-3}	9.83×10^{-3}	1.22	2.16	9.01×10^{-1}
TENM3	3.56×10^{-3}	2.05×10^{-2}	1.03	2.15	1.00
FER1L5	1.77×10^{-3}	1.18×10^{-2}	1.67	2.15	1.54
ZFHX2	3.46×10^{-3}	2.01×10^{-2}	1.68	2.14	1.09
CHP2	1.49×10^{-3}	1.03×10^{-2}	2.05	2.13	7.64×10^{-1}
UMODL1-AS1	1.88×10^{-5}	3.19×10^{-4}	3.84	2.13	4.96×10^{-1}
CD300C	4.80×10^{-3}	2.59×10^{-2}	1.37	2.13	6.59×10^{-1}

CLEC17A	4.16×10^{-6}	9.29×10^{-5}	1.80	2.13	6.72×10^{-1}
KLRG1	4.83×10^{-4}	4.16×10^{-3}	1.22	2.12	9.23×10^{-1}
NETO2	3.40×10^{-3}	1.99×10^{-2}	1.08	2.11	9.32×10^{-1}
TLR8	1.61×10^{-3}	1.09×10^{-2}	2.06	2.09	4.45×10^{-1}
HOST2	4.00×10^{-12}	3.80×10^{-10}	2.09	2.09	4.89×10^{-1}
LINC00539	2.03×10^{-3}	1.31×10^{-2}	1.01	2.09	9.92×10^{-1}
TTLL6	4.97×10^{-4}	4.27×10^{-3}	2.99	2.08	1.06
IL11	1.20×10^{-16}	2.87×10^{-14}	2.41	2.08	4.18×10^{-1}
ACRBP	4.54×10^{-4}	3.97×10^{-3}	1.12	2.08	9.76×10^{-1}
LMNTD1	4.71×10^{-4}	4.08×10^{-3}	3.87	2.07	7.23×10^{-1}
BTG4	4.17×10^{-4}	3.69×10^{-3}	2.65	2.06	8.27×10^{-1}
KIF11	1.91×10^{-3}	1.25×10^{-2}	1.44	2.06	7.57×10^{-1}
TRG-AS1	5.00×10^{-3}	2.67×10^{-2}	1.34	2.04	9.59×10^{-1}
ADAMDEC1	2.61×10^{-4}	2.59×10^{-3}	1.37	2.03	7.82×10^{-1}
HRH4	1.06×10^{-2}	4.80×10^{-2}	1.57	2.03	3.49×10^{-1}

FAM71E2	2.46×10^{-9}	1.29×10^{-7}	3.08	2.02	3.68×10^{-1}
NXPH4	2.39×10^{-25}	2.26×10^{-22}	2.78	2.01	2.94×10^{-1}
ADH6	1.08×10^{-3}	7.92×10^{-3}	1.90	2.01	7.34×10^{-1}
SIGLEC5	9.16×10^{-4}	6.89×10^{-3}	1.59	2.00	5.01×10^{-1}
GNG4	1.09×10^{-8}	5.02×10^{-7}	2.20	1.99	5.28×10^{-1}
E2F8	3.41×10^{-3}	1.99×10^{-2}	1.71	1.98	8.87×10^{-1}
TCL1A	6.47×10^{-9}	3.14×10^{-7}	3.19	1.98	2.54×10^{-1}
FAM167A	2.37×10^{-6}	5.67×10^{-5}	1.38	1.97	7.88×10^{-1}
BLM	1.93×10^{-3}	1.26×10^{-2}	1.11	1.97	8.26×10^{-1}
CERKL	3.86×10^{-4}	3.47×10^{-3}	3.48	1.96	5.09×10^{-1}
WNT3A	4.14×10^{-3}	2.31×10^{-2}	1.34	1.96	8.45×10^{-1}
ARHGAP22	2.51×10^{-4}	2.52×10^{-3}	1.15	1.93	9.28×10^{-1}
UHRF1	6.17×10^{-3}	3.17×10^{-2}	1.59	1.92	5.44×10^{-1}
KIF2C	9.52×10^{-3}	4.41×10^{-2}	1.05	1.91	9.07×10^{-1}
GDA	1.27×10^{-16}	3.00×10^{-14}	3.04	1.90	2.43×10^{-1}

KRT42P	7.83×10^{-5}	9.99×10^{-4}	1.81	1.88	6.01×10^{-1}
ADAMTS12	1.08×10^{-8}	4.96×10^{-7}	1.66	1.88	5.56×10^{-1}
ALOX12P2	8.04×10^{-4}	6.27×10^{-3}	1.11	1.88	1.05
RGS13	8.51×10^{-7}	2.33×10^{-5}	1.40	1.86	6.53×10^{-1}
RAB3B	6.01×10^{-17}	1.56×10^{-14}	3.35	1.85	2.33×10^{-1}
DQX1	4.61×10^{-3}	2.50×10^{-2}	1.51	1.85	7.89×10^{-1}
LY6H	7.32×10^{-3}	3.61×10^{-2}	1.90	1.83	1.31
APELA	2.93×10^{-3}	1.77×10^{-2}	2.80	1.82	3.23×10^{-1}
RNF157-AS1	1.70×10^{-4}	1.85×10^{-3}	1.98	1.81	7.70×10^{-1}
PRR16	1.10×10^{-3}	7.96×10^{-3}	1.47	1.81	5.76×10^{-1}
LINGO3	1.17×10^{-6}	3.09×10^{-5}	2.37	1.80	4.09×10^{-1}
MYO7A	2.21×10^{-3}	1.40×10^{-2}	1.36	1.79	6.97×10^{-1}
GBP6	1.66×10^{-3}	1.12×10^{-2}	1.95	1.79	8.86×10^{-1}
CHST13	2.12×10^{-4}	2.21×10^{-3}	2.45	1.78	3.00×10^{-1}
CEACAM7	7.60×10^{-4}	6.02×10^{-3}	2.33	1.77	2.15×10^{-1}

KIAA1211	2.62×10^{-4}	2.59×10^{-3}	2.47	1.77	7.29×10^{-1}
SIGLEC14	2.66×10^{-3}	1.63×10^{-2}	1.87	1.77	3.56×10^{-1}
LINC00240	8.99×10^{-4}	6.79×10^{-3}	1.14	1.76	8.37×10^{-1}
LOC100506585	4.91×10^{-6}	1.08×10^{-4}	2.89	1.75	2.45×10^{-1}
TTC23L	1.10×10^{-2}	4.94×10^{-2}	1.12	1.75	9.77×10^{-1}
CCDC184	7.02×10^{-3}	3.49×10^{-2}	1.10	1.75	8.27×10^{-1}
CLEC4F	7.88×10^{-4}	6.17×10^{-3}	1.78	1.74	9.05×10^{-1}
RDH10-AS1	1.30×10^{-3}	9.18×10^{-3}	1.23	1.73	8.47×10^{-1}
SLC22A20	8.01×10^{-4}	6.26×10^{-3}	1.25	1.73	8.00×10^{-1}
LOC101927267	9.48×10^{-4}	7.07×10^{-3}	2.27	1.68	8.40×10^{-1}
LOC105747689	2.93×10^{-5}	4.65×10^{-4}	1.49	1.68	5.97×10^{-1}
CASC10	4.49×10^{-7}	1.31×10^{-5}	1.72	1.67	5.80×10^{-1}
HS3ST4	1.84×10^{-3}	1.21×10^{-2}	2.82	1.67	2.15×10^{-1}
RAET1E	3.89×10^{-3}	2.20×10^{-2}	1.71	1.67	8.40×10^{-1}
LINC01187	9.69×10^{-6}	1.89×10^{-4}	3.27	1.66	3.55×10^{-1}

P4HA3	3.40×10^{-4}	3.16×10^{-3}	1.29	1.66	5.68×10^{-1}
NOXRED1	7.53×10^{-3}	3.69×10^{-2}	1.32	1.64	7.25×10^{-1}
FRAS1	8.45×10^{-5}	1.06×10^{-3}	1.31	1.64	5.91×10^{-1}
C12orf74	1.02×10^{-4}	1.24×10^{-3}	2.33	1.63	4.38×10^{-1}
SLX4IP	4.82×10^{-3}	2.60×10^{-2}	1.17	1.62	7.75×10^{-1}
ARL11	1.02×10^{-7}	3.57×10^{-6}	2.75	1.61	3.02×10^{-1}
F12	6.34×10^{-3}	3.24×10^{-2}	1.34	1.60	5.59×10^{-1}
PAX5	1.65×10^{-6}	4.14×10^{-5}	1.64	1.58	5.91×10^{-1}
PLK4	1.38×10^{-3}	9.64×10^{-3}	1.27	1.57	6.38×10^{-1}
CCNE1	1.98×10^{-3}	1.29×10^{-2}	1.01	1.57	7.30×10^{-1}
LINC00908	2.78×10^{-4}	2.71×10^{-3}	1.45	1.57	6.82×10^{-1}
VGLL1	1.13×10^{-3}	8.15×10^{-3}	1.20	1.56	6.46×10^{-1}
ARHGAP11A	1.10×10^{-3}	7.96×10^{-3}	1.28	1.54	6.04×10^{-1}
USH1C	8.49×10^{-3}	4.03×10^{-2}	1.22	1.54	9.62×10^{-1}
E2F2	8.79×10^{-3}	4.14×10^{-2}	1.50	1.53	5.27×10^{-1}

RAB42	5.26×10^{-3}	2.78×10^{-2}	1.01	1.51	7.01×10^{-1}
RUFY4	2.92×10^{-3}	1.76×10^{-2}	1.19	1.51	7.69×10^{-1}
NUF2	9.06×10^{-4}	6.83×10^{-3}	1.54	1.50	4.80×10^{-1}
BNC1	2.46×10^{-4}	2.48×10^{-3}	2.10	1.50	5.45×10^{-1}
KIF6	2.64×10^{-4}	2.61×10^{-3}	2.39	1.49	6.59×10^{-1}
TREML1	4.62×10^{-4}	4.02×10^{-3}	1.71	1.48	4.99×10^{-1}
GDNF-AS1	1.65×10^{-3}	1.12×10^{-2}	2.44	1.46	2.81×10^{-1}
ACOXL	5.91×10^{-3}	3.07×10^{-2}	1.16	1.45	4.68×10^{-1}
NAGS	1.31×10^{-6}	3.39×10^{-5}	1.24	1.45	6.19×10^{-1}
TRPM6	3.50×10^{-5}	5.35×10^{-4}	1.72	1.45	4.25×10^{-1}
CCNJL	2.15×10^{-4}	2.24×10^{-3}	1.06	1.44	6.34×10^{-1}
EPPIN-WFDC6	1.45×10^{-5}	2.59×10^{-4}	2.85	1.44	4.75×10^{-1}
AADACP1	9.64×10^{-3}	4.46×10^{-2}	1.26	1.44	6.66×10^{-1}
LINC01605	1.10×10^{-2}	4.96×10^{-2}	1.11	1.43	9.26×10^{-1}
NLRC4	2.15×10^{-3}	1.37×10^{-2}	1.64	1.43	4.31×10^{-1}

KIAA0319	1.04×10^{-2}	4.73×10^{-2}	1.47	1.42	6.65×10^{-1}
AOC1	1.80×10^{-4}	1.94×10^{-3}	2.75	1.42	2.38×10^{-1}
TFEC	4.34×10^{-3}	2.39×10^{-2}	1.30	1.40	5.72×10^{-1}
IQCH	7.37×10^{-4}	5.87×10^{-3}	2.01	1.39	5.33×10^{-1}
FBXW10	8.62×10^{-3}	4.08×10^{-2}	1.25	1.38	8.36×10^{-1}
LINC01018	2.52×10^{-8}	1.07×10^{-6}	2.15	1.38	3.04×10^{-1}
TRAT1	1.04×10^{-4}	1.25×10^{-3}	1.28	1.37	5.49×10^{-1}
CLDN16	3.44×10^{-4}	3.18×10^{-3}	1.78	1.37	5.81×10^{-1}
KIAA1257	1.05×10^{-3}	7.74×10^{-3}	2.02	1.36	5.69×10^{-1}
TMEM151B	6.54×10^{-3}	3.31×10^{-2}	1.65	1.36	6.93×10^{-1}
RIMBP2	1.20×10^{-14}	1.83×10^{-12}	2.44	1.36	2.28×10^{-1}
GJB6	8.39×10^{-4}	6.46×10^{-3}	2.35	1.33	3.93×10^{-1}
MASP1	2.85×10^{-3}	1.73×10^{-2}	1.57	1.33	4.04×10^{-1}
CLNK	8.73×10^{-8}	3.10×10^{-6}	2.55	1.33	2.85×10^{-1}
CD226	3.83×10^{-3}	2.18×10^{-2}	1.15	1.31	5.80×10^{-1}

FSD1L	8.02×10^{-3}	3.85×10^{-2}	1.30	1.30	5.79×10^{-1}
CFAP54	2.54×10^{-3}	1.57×10^{-2}	1.66	1.30	5.76×10^{-1}
CCDC67	7.86×10^{-3}	3.80×10^{-2}	1.73	1.29	5.40×10^{-1}
CFAP47	6.62×10^{-5}	8.73×10^{-4}	3.41	1.29	4.67×10^{-1}
ELFN1	2.47×10^{-4}	2.49×10^{-3}	1.21	1.29	5.55×10^{-1}
CASC2	2.21×10^{-3}	1.40×10^{-2}	1.96	1.28	4.69×10^{-1}
RGS7	1.11×10^{-4}	1.31×10^{-3}	2.46	1.27	2.49×10^{-1}
FBXL13	2.96×10^{-3}	1.78×10^{-2}	1.46	1.27	6.62×10^{-1}
C20orf197	9.81×10^{-7}	2.64×10^{-5}	2.89	1.27	1.68×10^{-1}
LINC00954	1.04×10^{-4}	1.25×10^{-3}	1.21	1.26	5.78×10^{-1}
UGT1A6	1.71×10^{-3}	1.15×10^{-2}	2.26	1.24	3.75×10^{-1}
CHST5	8.56×10^{-3}	4.06×10^{-2}	1.32	1.24	6.85×10^{-1}
C2orf73	1.35×10^{-3}	9.48×10^{-3}	3.26	1.24	3.57×10^{-1}
TMEM63C	3.44×10^{-3}	2.00×10^{-2}	1.10	1.24	6.50×10^{-1}
BBOX1-AS1	5.72×10^{-3}	2.98×10^{-2}	1.32	1.23	6.28×10^{-1}

KIF26B	1.47×10^{-4}	1.65×10^{-3}	1.69	1.23	3.29×10^{-1}
CES1P1	3.47×10^{-3}	2.01×10^{-2}	1.52	1.23	5.31×10^{-1}
TMEM132E	7.30×10^{-4}	5.83×10^{-3}	1.22	1.21	4.09×10^{-1}
WASIR2	3.01×10^{-4}	2.87×10^{-3}	1.78	1.20	4.58×10^{-1}
PLAG1	6.87×10^{-4}	5.55×10^{-3}	1.22	1.18	4.84×10^{-1}
ANKFN1	1.37×10^{-4}	1.56×10^{-3}	2.86	1.16	4.58×10^{-1}
ANXA8	1.15×10^{-4}	1.36×10^{-3}	1.93	1.16	4.21×10^{-1}
TENM2	5.63×10^{-4}	4.72×10^{-3}	1.55	1.16	3.91×10^{-1}
C10orf105	1.81×10^{-4}	1.94×10^{-3}	1.35	1.15	5.01×10^{-1}
LOC145694	3.98×10^{-4}	3.56×10^{-3}	2.11	1.14	3.73×10^{-1}
CATSPERD	1.19×10^{-4}	1.39×10^{-3}	3.23	1.14	4.33×10^{-1}
NEK2	1.58×10^{-3}	1.08×10^{-2}	1.99	1.11	3.40×10^{-1}
HOXB4	3.53×10^{-3}	2.04×10^{-2}	1.82	1.11	4.44×10^{-1}
LOC100996291	8.22×10^{-4}	6.37×10^{-3}	1.68	1.10	4.27×10^{-1}
TXK	5.97×10^{-3}	3.09×10^{-2}	1.13	1.10	5.24×10^{-1}

C12orf56	4.65×10^{-13}	5.44×10^{-11}	2.58	1.09	1.87×10^{-1}
GVINP1	2.98×10^{-4}	2.85×10^{-3}	1.23	1.09	5.05×10^{-1}
NTNG2	1.03×10^{-4}	1.25×10^{-3}	1.57	1.08	3.31×10^{-1}
CDC25A	2.09×10^{-4}	2.19×10^{-3}	1.06	1.06	5.05×10^{-1}
IGF2-AS	3.97×10^{-5}	5.92×10^{-4}	1.44	1.06	4.06×10^{-1}
SLIT1	4.93×10^{-3}	2.65×10^{-2}	1.71	1.06	5.69×10^{-1}
LOC101928075	2.94×10^{-3}	1.77×10^{-2}	1.63	1.05	5.24×10^{-1}
MOV10L1	3.27×10^{-6}	7.57×10^{-5}	1.94	1.05	2.37×10^{-1}
CDH22	2.16×10^{-12}	2.16×10^{-10}	4.17	1.05	5.57×10^{-2}
E2F7	1.43×10^{-4}	1.62×10^{-3}	2.08	1.04	3.04×10^{-1}
FBN2	5.86×10^{-5}	7.94×10^{-4}	1.89	1.04	3.02×10^{-1}
LINC00939	9.27×10^{-3}	4.31×10^{-2}	1.09	1.04	4.96×10^{-1}
SH2D1B	1.35×10^{-6}	3.49×10^{-5}	1.65	1.03	3.19×10^{-1}
C9orf139	7.19×10^{-4}	5.77×10^{-3}	2.55	1.01	2.65×10^{-1}
SPOCD1	2.69×10^{-3}	1.65×10^{-2}	1.34	1.01	3.23×10^{-1}

ST8SIA1	3.13×10^{-5}	4.91×10^{-4}	1.82	1.00	2.83×10^{-1}
ARHGEF26-AS1	4.23×10^{-4}	3.73×10^{-3}	1.34	9.98×10^{-1}	5.01×10^{-1}
FOXI2	2.16×10^{-3}	1.37×10^{-2}	1.49	9.89×10^{-1}	4.94×10^{-1}
TUBA3FP	3.78×10^{-3}	2.15×10^{-2}	1.58	9.85×10^{-1}	5.31×10^{-1}
PROC	8.30×10^{-3}	3.96×10^{-2}	1.09	9.78×10^{-1}	5.14×10^{-1}
FAM159A	2.72×10^{-4}	2.67×10^{-3}	1.22	9.78×10^{-1}	4.22×10^{-1}
NCAPH	7.76×10^{-3}	3.77×10^{-2}	1.20	9.76×10^{-1}	3.75×10^{-1}
DCANP1	4.87×10^{-3}	2.62×10^{-2}	1.56	9.71×10^{-1}	3.44×10^{-1}
LINC00900	1.59×10^{-3}	1.08×10^{-2}	1.20	9.70×10^{-1}	4.52×10^{-1}
HMMR	2.96×10^{-4}	2.83×10^{-3}	1.77	9.61×10^{-1}	2.48×10^{-1}
LINC01215	1.33×10^{-5}	2.43×10^{-4}	1.28	9.31×10^{-1}	3.98×10^{-1}
TRIM61	8.12×10^{-4}	6.31×10^{-3}	1.15	9.11×10^{-1}	4.34×10^{-1}
PRAMENP	3.67×10^{-3}	2.10×10^{-2}	1.17	8.99×10^{-1}	4.16×10^{-1}
ZNF382	1.74×10^{-4}	1.89×10^{-3}	1.32	8.95×10^{-1}	3.98×10^{-1}
GCSAML	9.00×10^{-4}	6.80×10^{-3}	1.46	8.92×10^{-1}	3.28×10^{-1}

POLQ	7.97×10^{-3}	3.83×10^{-2}	1.69	8.90×10^{-1}	4.74×10^{-1}
ATP8A2	1.52×10^{-5}	2.68×10^{-4}	2.53	8.88×10^{-1}	1.72×10^{-1}
KMO	1.26×10^{-4}	1.46×10^{-3}	1.59	8.84×10^{-1}	2.61×10^{-1}
CDK15	6.44×10^{-5}	8.56×10^{-4}	1.42	8.80×10^{-1}	3.17×10^{-1}
SERPINB7	2.12×10^{-3}	1.35×10^{-2}	1.57	8.79×10^{-1}	3.19×10^{-1}
LEKR1	7.27×10^{-3}	3.59×10^{-2}	1.19	8.65×10^{-1}	4.00×10^{-1}
TTK	3.95×10^{-3}	2.22×10^{-2}	1.49	8.63×10^{-1}	2.66×10^{-1}
LINC01296	2.68×10^{-4}	2.64×10^{-3}	1.44	8.44×10^{-1}	2.87×10^{-1}
FCRLB	9.54×10^{-3}	4.41×10^{-2}	1.08	8.41×10^{-1}	4.19×10^{-1}
LOC100507351	3.48×10^{-4}	3.21×10^{-3}	1.11	8.14×10^{-1}	3.86×10^{-1}
C6	4.40×10^{-3}	2.42×10^{-2}	2.06	8.08×10^{-1}	3.80×10^{-1}
RP1	1.36×10^{-4}	1.55×10^{-3}	3.71	8.04×10^{-1}	2.42×10^{-1}
SSC4D	6.36×10^{-6}	1.33×10^{-4}	1.37	7.95×10^{-1}	2.95×10^{-1}
TNFRSF8	1.69×10^{-4}	1.84×10^{-3}	1.25	7.63×10^{-1}	3.16×10^{-1}
TG	3.13×10^{-6}	7.27×10^{-5}	1.47	7.57×10^{-1}	2.98×10^{-1}

TFR2	1.13×10^{-4}	1.34×10^{-3}	1.37	7.46×10^{-1}	2.85×10^{-1}
DRD1	4.63×10^{-4}	4.02×10^{-3}	1.28	7.46×10^{-1}	2.98×10^{-1}
ZNF488	5.03×10^{-3}	2.68×10^{-2}	1.58	7.38×10^{-1}	2.63×10^{-1}
RNF144A-AS1	8.70×10^{-4}	6.62×10^{-3}	1.30	7.34×10^{-1}	3.27×10^{-1}
PTPRO	8.74×10^{-4}	6.65×10^{-3}	1.57	7.34×10^{-1}	2.17×10^{-1}
TPTE2	4.73×10^{-3}	2.56×10^{-2}	1.40	7.31×10^{-1}	2.70×10^{-1}
PTCHD4	3.84×10^{-3}	2.18×10^{-2}	1.16	7.14×10^{-1}	2.97×10^{-1}
PRND	2.47×10^{-5}	4.06×10^{-4}	4.36	7.13×10^{-1}	6.07×10^{-2}
DSCAML1	1.01×10^{-2}	4.63×10^{-2}	1.14	7.12×10^{-1}	4.25×10^{-1}
C10orf67	1.65×10^{-3}	1.12×10^{-2}	2.82	7.04×10^{-1}	3.84×10^{-1}
IGF2	1.95×10^{-7}	6.25×10^{-6}	1.48	6.98×10^{-1}	2.39×10^{-1}
PHEX	5.27×10^{-4}	4.47×10^{-3}	1.53	6.86×10^{-1}	2.21×10^{-1}
KCNJ1	1.06×10^{-3}	7.76×10^{-3}	1.76	6.86×10^{-1}	1.98×10^{-1}
PPP4R4	6.90×10^{-6}	1.43×10^{-4}	1.50	6.76×10^{-1}	3.03×10^{-1}
IGF2BP3	1.85×10^{-3}	1.22×10^{-2}	2.03	6.64×10^{-1}	1.60×10^{-1}

LOC101927780	3.02×10^{-3}	1.81×10^{-2}	2.25	6.64×10^{-1}	1.62×10^{-1}
BBOX1	1.42×10^{-3}	9.84×10^{-3}	1.74	6.58×10^{-1}	2.87×10^{-1}
MICALCL	7.72×10^{-4}	6.09×10^{-3}	1.22	6.55×10^{-1}	2.95×10^{-1}
RASGRF1	1.48×10^{-7}	4.89×10^{-6}	1.94	6.40×10^{-1}	1.90×10^{-1}
MYBPC3	3.27×10^{-4}	3.07×10^{-3}	1.51	6.39×10^{-1}	2.01×10^{-1}
NOX4	5.62×10^{-5}	7.72×10^{-4}	1.31	6.27×10^{-1}	2.38×10^{-1}
PMFBP1	8.43×10^{-4}	6.48×10^{-3}	1.04	6.12×10^{-1}	3.12×10^{-1}
CNGA3	2.73×10^{-3}	1.67×10^{-2}	2.68	6.07×10^{-1}	3.77×10^{-1}
CALML3-AS1	4.82×10^{-5}	6.84×10^{-4}	1.52	5.99×10^{-1}	2.29×10^{-1}
ABCA4	3.02×10^{-6}	7.06×10^{-5}	1.72	5.90×10^{-1}	2.18×10^{-1}
ITGAD	9.02×10^{-4}	6.81×10^{-3}	1.42	5.82×10^{-1}	1.89×10^{-1}
OGDHL	5.81×10^{-3}	3.02×10^{-2}	1.13	5.73×10^{-1}	2.70×10^{-1}
ABCB11	3.87×10^{-5}	5.80×10^{-4}	2.47	5.59×10^{-1}	1.86×10^{-1}
CCDC178	1.78×10^{-3}	1.18×10^{-2}	1.45	5.56×10^{-1}	2.07×10^{-1}
AKAP5	4.32×10^{-4}	3.80×10^{-3}	1.05	5.53×10^{-1}	2.76×10^{-1}

FSIP1	$3.89{\times}10^{-3}$	$2.20{\times}10^{-2}$	1.36	$5.17{\times}10^{-1}$	$2.48{\times}10^{-1}$
TPTE2P1	$3.67{\times}10^{-3}$	$2.10{\times}10^{-2}$	1.31	$5.08{\times}10^{-1}$	$2.82{\times}10^{-1}$
GPRIN1	$4.85{\times}10^{-3}$	$2.61{\times}10^{-2}$	1.00	$5.02{\times}10^{-1}$	$2.67{\times}10^{-1}$
PAQR5	$4.72{\times}10^{-4}$	$4.08{\times}10^{-3}$	1.76	$4.96{\times}10^{-1}$	$1.90{\times}10^{-1}$
KCNH1	$4.63{\times}10^{-4}$	$4.02{\times}10^{-3}$	1.73	$4.82{\times}10^{-1}$	$1.50{\times}10^{-1}$
ANKRD18A	$6.90{\times}10^{-3}$	$3.45{\times}10^{-2}$	1.35	$4.26{\times}10^{-1}$	$2.13{\times}10^{-1}$
COL22A1	$6.41{\times}10^{-4}$	$5.23{\times}10^{-3}$	1.34	$4.22{\times}10^{-1}$	$1.78{\times}10^{-1}$
UNC13A	$1.30{\times}10^{-3}$	$9.20{\times}10^{-3}$	1.59	$4.02{\times}10^{-1}$	$1.27{\times}10^{-1}$
ERVMER34-1	$3.29{\times}10^{-4}$	$3.08{\times}10^{-3}$	1.26	$3.98{\times}10^{-1}$	$1.74{\times}10^{-1}$
PTGER4P2-CDK2AP2P2	$2.13{\times}10^{-3}$	$1.36{\times}10^{-2}$	1.42	$3.98{\times}10^{-1}$	$1.96{\times}10^{-1}$
FREM2	$1.00{\times}10^{-2}$	$4.59{\times}10^{-2}$	1.09	$3.89{\times}10^{-1}$	$2.13{\times}10^{-1}$
CNTNAP2	$3.69{\times}10^{-5}$	$5.60{\times}10^{-4}$	2.12	$3.71{\times}10^{-1}$	$9.53{\times}10^{-2}$
MOBP	$3.99{\times}10^{-3}$	$2.24{\times}10^{-2}$	2.08	$3.69{\times}10^{-1}$	$2.45{\times}10^{-1}$
MYLK3	$8.08{\times}10^{-5}$	$1.02{\times}10^{-3}$	1.63	$3.29{\times}10^{-1}$	$1.10{\times}10^{-1}$
GUCY2D	$1.78{\times}10^{-4}$	$1.92{\times}10^{-3}$	2.05	$3.24{\times}10^{-1}$	$1.09{\times}10^{-1}$

PAK7	4.37×10^{-4}	3.84×10^{-3}	1.31	3.14×10^{-1}	1.20×10^{-1}
LRRC37A6P	1.66×10^{-3}	1.12×10^{-2}	1.54	3.05×10^{-1}	1.09×10^{-1}
CASC5	2.19×10^{-4}	2.26×10^{-3}	2.01	2.97×10^{-1}	7.07×10^{-2}
SCN11A	1.11×10^{-3}	8.08×10^{-3}	1.27	2.62×10^{-1}	1.24×10^{-1}
ZNF804A	2.29×10^{-3}	1.44×10^{-2}	1.31	2.58×10^{-1}	1.29×10^{-1}
FLG	3.02×10^{-3}	1.81×10^{-2}	1.22	1.71×10^{-1}	8.27×10^{-2}
GRM4	3.41×10^{-3}	1.99×10^{-2}	2.23	1.65×10^{-1}	6.82×10^{-2}
HLA-DOA	6.96×10^{-5}	9.10×10^{-4}	1.42	1.61×10^{-1}	7.62×10^{-2}

Supplementary Table S2

Downregulated genes in NP by comparison with those in IT (log fold change < -1).

Gene name	*P* value	Adjusted *P* value (FDR)	Log fold change	NP (FPKM)	IT (FPKM)
BPIFA1	1.40×10^{-3}	9.77×10^{-3}	-3.01	4.13×10^{3}	2.66×10^{4}
SLPI	9.77×10^{-7}	2.63×10^{-5}	-2.16	3.00×10^{3}	1.11×10^{4}
BPIFB1	9.02×10^{-5}	1.12×10^{-3}	-2.74	2.02×10^{3}	1.18×10^{4}
STATH	7.00×10^{-13}	7.96×10^{-11}	-10.8	9.14×10^{2}	6.71×10^{4}
LTF	1.29×10^{-9}	7.28×10^{-8}	-4.47	8.27×10^{2}	6.92×10^{3}
XBP1	1.04×10^{-3}	7.65×10^{-3}	-1.00	6.40×10^{2}	1.18×10^{3}
LYZ	4.03×10^{-16}	8.61×10^{-14}	-5.37	4.56×10^{2}	1.40×10^{4}
PIGR	4.81×10^{-9}	2.40×10^{-7}	-2.50	3.24×10^{2}	1.66×10^{3}
CXCL17	1.11×10^{-3}	8.08×10^{-3}	-1.36	2.60×10^{2}	6.52×10^{2}
P4HB	1.89×10^{-5}	3.21×10^{-4}	-1.07	2.48×10^{2}	5.23×10^{2}
MSMB	2.58×10^{-8}	1.08×10^{-6}	-4.25	2.40×10^{2}	2.33×10^{3}

MYL9	1.78×10^{-3}	1.18×10^{-2}	-1.06	2.07×10^{2}	3.87×10^{2}
TAGLN	4.36×10^{-4}	3.84×10^{-3}	-1.34	1.98×10^{2}	4.37×10^{2}
FDCSP	3.76×10^{-6}	8.52×10^{-5}	-3.15	1.84×10^{2}	8.46×10^{2}
CYR61	1.90×10^{-4}	2.02×10^{-3}	-1.17	1.44×10^{2}	3.24×10^{2}
ZG16B	8.28×10^{-15}	1.32×10^{-12}	-7.16	1.43×10^{2}	4.71×10^{3}
AZGP1	7.43×10^{-9}	3.57×10^{-7}	-5.05	1.29×10^{2}	1.92×10^{3}
DMBT1	1.38×10^{-5}	2.49×10^{-4}	-4.56	1.22×10^{2}	1.46×10^{3}
NEAT1	4.00×10^{-5}	5.95×10^{-4}	-1.18	1.20×10^{2}	2.94×10^{2}
CSRP1	1.12×10^{-10}	7.94×10^{-9}	-1.39	1.00×10^{2}	2.59×10^{2}
KRT7	4.39×10^{-7}	1.29×10^{-5}	-1.72	9.92×10	3.11×10^{2}
TCN1	4.26×10^{-6}	9.45×10^{-5}	-3.22	9.00×10	5.53×10^{2}
NUCB2	3.24×10^{-7}	9.89×10^{-6}	-1.19	8.04×10	1.86×10^{2}
LMNA	5.52×10^{-3}	2.90×10^{-2}	-1.05	7.87×10	2.04×10^{2}
ATP2A3	5.26×10^{-12}	4.83×10^{-10}	-2.37	7.79×10	3.76×10^{2}
IGFBP5	3.80×10^{-4}	3.42×10^{-3}	-1.47	7.62×10	2.63×10^{2}

MFAP4	$6.38×10^{-5}$	$8.49×10^{-4}$	-1.46	7.23×10	$1.94×10^{2}$
ID1	$1.35×10^{-3}$	$9.49×10^{-3}$	-1.18	6.97×10	$1.67×10^{2}$
ACTA2	$4.92×10^{-8}$	$1.88×10^{-6}$	-1.98	6.85×10	$2.40×10^{2}$
AQP5	$9.31×10^{-8}$	$3.29×10^{-6}$	-2.17	6.80×10	$2.84×10^{2}$
TIMP3	$2.35×10^{-4}$	$2.39×10^{-3}$	-1.11	6.48×10	$1.29×10^{2}$
MUC5B	$5.08×10^{-5}$	$7.14×10^{-4}$	-4.04	6.13×10	$5.26×10^{2}$
GOLM1	$1.36×10^{-3}$	$9.50×10^{-3}$	-1.30	5.58×10	$1.38×10^{2}$
FXYD3	$4.97×10^{-3}$	$2.66×10^{-2}$	-1.08	5.49×10	$1.07×10^{2}$
ADIRF	$1.43×10^{-4}$	$1.61×10^{-3}$	-1.31	5.41×10	$1.25×10^{2}$
EHF	$3.13×10^{-6}$	$7.27×10^{-5}$	-1.50	5.38×10	$1.58×10^{2}$
TM4SF1	$8.27×10^{-4}$	$6.39×10^{-3}$	-1.50	5.37×10	$2.15×10^{2}$
ID2	$2.03×10^{-5}$	$3.41×10^{-4}$	-1.23	5.13×10	$1.27×10^{2}$
TSPYL2	$6.34×10^{-7}$	$1.80×10^{-5}$	-1.06	5.12×10	$1.07×10^{2}$
CCDC3	$1.77×10^{-4}$	$1.92×10^{-3}$	-1.19	5.07×10	$1.04×10^{2}$
NUPR1	$1.16×10^{-5}$	$2.18×10^{-4}$	-1.51	4.94×10	$1.50×10^{2}$

NDRG2	3.86×10^{-11}	3.02×10^{-9}	-2.64	4.82×10	2.68×10^{2}
MGLL	5.08×10^{-8}	1.93×10^{-6}	-1.33	4.70×10	1.16×10^{2}
NPDC1	1.17×10^{-7}	3.97×10^{-6}	-1.20	4.48×10	1.06×10^{2}
CRACR2B	1.15×10^{-11}	1.01×10^{-9}	-2.07	3.81×10	1.47×10^{2}
TMEM63A	2.32×10^{-10}	1.53×10^{-8}	-1.43	3.79×10	9.92×10
PODXL	2.18×10^{-6}	5.26×10^{-5}	-1.05	3.78×10	7.91×10
SCGB3A1	8.99×10^{-4}	6.79×10^{-3}	-3.64	3.76×10	2.27×10^{2}
PYGB	1.78×10^{-10}	1.21×10^{-8}	-1.10	3.55×10	7.40×10
ODAM	6.77×10^{-11}	5.10×10^{-9}	-3.92	3.50×10	3.43×10^{2}
PDCD4	7.33×10^{-8}	2.66×10^{-6}	-1.17	3.38×10	7.75×10
DCXR	1.54×10^{-7}	5.08×10^{-6}	-1.17	3.37×10	7.70×10
HP	6.83×10^{-5}	8.96×10^{-4}	-6.10	3.30×10	2.46×10^{2}
BPIFB6	1.87×10^{-3}	1.23×10^{-2}	-6.39	3.08×10	7.54
EPS8L2	3.79×10^{-5}	5.72×10^{-4}	-1.00	3.02×10	5.75×10
PIP	2.23×10^{-19}	1.05×10^{-16}	-9.90	2.92×10	5.30×10^{3}

PDXDC2P	3.93×10^{-7}	1.17×10^{-5}	-1.05	2.84×10	5.95×10
PHB	2.28×10^{-7}	7.25×10^{-6}	-1.02	2.78×10	5.72×10
CLDN3	3.54×10^{-3}	2.05×10^{-2}	-1.15	2.76×10	5.00×10
RGCC	1.64×10^{-15}	3.14×10^{-13}	-1.73	2.69×10	9.31×10
ETV6	1.04×10^{-5}	2.00×10^{-4}	-1.11	2.69×10	6.36×10
HID1	9.42×10^{-8}	3.31×10^{-6}	-1.44	2.66×10	7.05×10
FAM3D	3.79×10^{-8}	1.51×10^{-6}	-2.84	2.66×10	1.46×10^{2}
MYH11	1.10×10^{-37}	3.55×10^{-34}	-3.74	2.56×10	3.12×10^{2}
NRBP2	2.73×10^{-8}	1.13×10^{-6}	-1.05	2.46×10	4.99×10
ALDH1A3	2.78×10^{-14}	3.91×10^{-12}	-1.59	2.43×10	7.31×10
NPIPB15	6.43×10^{-11}	4.86×10^{-9}	-1.16	2.43×10	5.44×10
PRSS8	2.60×10^{-8}	1.09×10^{-6}	-2.21	2.42×10	1.00×10^{2}
MLPH	3.13×10^{-8}	1.28×10^{-6}	-1.66	2.39×10	6.82×10
MCFD2	1.28×10^{-8}	5.83×10^{-7}	-1.13	2.37×10	5.35×10
TMPRSS2	2.58×10^{-5}	4.21×10^{-4}	-1.03	2.36×10	4.80×10

ALDH18A1	8.98×10^{-9}	4.24×10^{-7}	-1.26	2.31×10	5.74×10
BPIFB2	2.21×10^{-16}	4.92×10^{-14}	-7.32	2.30×10	1.07×10^{3}
SOD3	4.53×10^{-10}	2.81×10^{-8}	-1.43	2.29×10	5.91×10
GPCPD1	1.06×10^{-5}	2.03×10^{-4}	-1.00	2.25×10	4.59×10
KANK2	4.28×10^{-5}	6.26×10^{-4}	-1.07	2.24×10	4.57×10
PHGDH	1.13×10^{-5}	2.13×10^{-4}	-1.30	2.17×10	5.12×10
ACSL1	3.45×10^{-5}	5.31×10^{-4}	-1.27	2.17×10	5.01×10
GEM	2.92×10^{-3}	1.76×10^{-2}	-1.09	2.17×10	4.73×10
ANO1	1.16×10^{-9}	6.65×10^{-8}	-2.34	2.16×10	1.10×10^{2}
CHKA	8.02×10^{-11}	5.96×10^{-9}	-1.46	2.15×10	5.96×10
ADCY6	9.80×10^{-9}	4.56×10^{-7}	-1.02	2.15×10	4.24×10
TCEA3	8.43×10^{-7}	2.32×10^{-5}	-1.75	2.11×10	6.99×10
HOMER2	7.83×10^{-4}	6.15×10^{-3}	-1.12	2.10×10	4.67×10
FHL1	1.30×10^{-7}	4.36×10^{-6}	-2.03	2.09×10	7.66×10
LTBP4	1.60×10^{-8}	7.15×10^{-7}	-1.11	2.08×10	4.46×10

CREB3L1	2.92×10^{-7}	8.99×10^{-6}	-1.63	2.06×10	7.14×10
TMED3	2.03×10^{-8}	8.75×10^{-7}	-1.85	2.04×10	7.41×10
C11orf96	8.21×10^{-14}	1.07×10^{-11}	-2.53	2.03×10	1.25×10^{2}
PDZK1IP1	1.53×10^{-4}	1.70×10^{-3}	-2.30	1.99×10	5.74×10
SH3BP4	8.47×10^{-7}	2.32×10^{-5}	-1.42	1.99×10	5.45×10
SPDEF	2.37×10^{-7}	7.54×10^{-6}	-1.69	1.96×10	5.26×10
LRP5	5.46×10^{-9}	2.68×10^{-7}	-1.50	1.96×10	5.54×10
GALE	1.82×10^{-5}	3.10×10^{-4}	-1.05	1.93×10	3.98×10
SERINC2	8.20×10^{-4}	6.35×10^{-3}	-1.29	1.91×10	3.95×10
SFRP4	4.74×10^{-3}	2.56×10^{-2}	-1.12	1.87×10	3.65×10
NFIX	4.69×10^{-6}	1.03×10^{-4}	-1.40	1.86×10	4.63×10
ERBB3	2.22×10^{-4}	2.28×10^{-3}	-1.20	1.83×10	3.96×10
KCNN4	5.58×10^{-18}	1.92×10^{-15}	-3.25	1.78×10	1.36×10^{2}
KIAA1324	1.53×10^{-11}	1.31×10^{-9}	-2.69	1.78×10	1.11×10^{2}
PLAT	3.14×10^{-3}	1.86×10^{-2}	-1.41	1.78×10	3.97×10

C6orf58	8.82×10^{-15}	1.39×10^{-12}	-11.1	1.78×10	1.18×10^{3}
GPT2	1.77×10^{-4}	1.91×10^{-3}	-1.47	1.77×10	4.64×10
CLEC3B	3.18×10^{-4}	3.00×10^{-3}	-1.34	1.75×10	4.26×10
PHLDA1	2.48×10^{-10}	1.62×10^{-8}	-2.35	1.73×10	8.65×10
TM7SF2	8.12×10^{-4}	6.31×10^{-3}	-1.01	1.71×10	3.35×10
STK39	1.34×10^{-6}	3.48×10^{-5}	-1.42	1.70×10	4.71×10
TNFRSF19	4.16×10^{-5}	6.13×10^{-4}	-1.20	1.70×10	3.92×10
SOX13	2.71×10^{-5}	4.37×10^{-4}	-1.01	1.70×10	3.29×10
ST3GAL1	2.21×10^{-12}	2.19×10^{-10}	-1.26	1.68×10	4.05×10
ELF5	3.08×10^{-4}	2.93×10^{-3}	-1.50	1.68×10	4.62×10
LZTS3	4.74×10^{-5}	6.76×10^{-4}	-1.05	1.67×10	3.35×10
KLK11	3.08×10^{-3}	1.84×10^{-2}	-1.57	1.67×10	5.18×10
CHPT1	3.02×10^{-21}	1.87×10^{-18}	-1.82	1.66×10	5.67×10
MYLK	1.67×10^{-11}	1.41×10^{-9}	-1.34	1.65×10	4.00×10
PPP1R1B	2.78×10^{-12}	2.72×10^{-10}	-4.07	1.64×10	1.67×10^{2}

ST3GAL4	3.03×10^{-8}	1.24×10^{-6}	-1.35	1.62×10	3.89×10
TFCP2L1	3.16×10^{-4}	2.98×10^{-3}	-1.20	1.62×10	3.52×10
MIR324	7.37×10^{-3}	3.63×10^{-2}	-1.03	1.58×10	3.78×10
TFF1	9.50×10^{-6}	1.86×10^{-4}	-4.56	1.57×10	1.05×10^{2}
S100A1	4.14×10^{-10}	2.59×10^{-8}	-3.75	1.55×10	1.60×10^{2}
HIP1R	9.50×10^{-9}	4.44×10^{-7}	-1.32	1.53×10	3.79×10
PLIN2	9.24×10^{-4}	6.94×10^{-3}	-1.32	1.53×10	4.84×10
TGFB1I1	6.94×10^{-6}	1.43×10^{-4}	-1.21	1.52×10	3.29×10
KRT14	1.21×10^{-4}	1.41×10^{-3}	-3.45	1.50×10	9.20×10
SLC9A1	4.07×10^{-9}	2.06×10^{-7}	-1.13	1.50×10	3.32×10
FERMT2	1.57×10^{-6}	3.95×10^{-5}	-1.09	1.50×10	3.07×10
FAM114A1	8.16×10^{-8}	2.93×10^{-6}	-1.02	1.50×10	3.11×10
BHLHA15	3.91×10^{-5}	5.85×10^{-4}	-1.89	1.49×10	4.17×10
FILIP1L	6.93×10^{-3}	3.47×10^{-2}	-1.00	1.47×10	2.99×10
MIR145	4.21×10^{-8}	1.64×10^{-6}	-1.87	1.47×10	5.39×10

LMOD1	1.87×10^{-10}	1.26×10^{-8}	-2.39	1.46×10	6.55×10
ACAT1	7.43×10^{-7}	2.07×10^{-5}	-1.05	1.42×10	2.97×10
FBP1	3.12×10^{-10}	2.00×10^{-8}	-1.61	1.40×10	4.04×10
RNASE4	1.04×10^{-7}	3.61×10^{-6}	-1.44	1.38×10	3.84×10
KLF9	1.16×10^{-3}	8.32×10^{-3}	-1.17	1.37×10	3.24×10
EIF4EBP1	1.19×10^{-5}	2.22×10^{-4}	-1.55	1.36×10	3.94×10
PYCR1	1.86×10^{-8}	8.08×10^{-7}	-2.02	1.36×10	4.87×10
CTNNBIP1	3.02×10^{-7}	9.30×10^{-6}	-1.41	1.35×10	3.66×10
ASAP3	5.30×10^{-7}	1.52×10^{-5}	-1.04	1.35×10	2.75×10
HSD17B2	6.65×10^{-4}	5.38×10^{-3}	-1.26	1.34×10	3.22×10
SLC39A8	4.24×10^{-3}	2.35×10^{-2}	-1.08	1.34×10	2.23×10
MANSC1	2.28×10^{-13}	2.81×10^{-11}	-2.23	1.31×10	5.91×10
ARHGEF25	1.04×10^{-4}	1.25×10^{-3}	-1.11	1.31×10	2.73×10
LRIG3	1.95×10^{-4}	2.06×10^{-3}	-1.06	1.31×10	2.64×10
HSF4	5.10×10^{-9}	2.52×10^{-7}	-1.19	1.30×10	3.01×10

TSPAN8	$3.35×10^{-4}$	$3.12×10^{-3}$	-1.87	1.29×10	4.46×10
ITLN1	$5.78×10^{-4}$	$4.82×10^{-3}$	-3.71	1.28×10	$1.55×10^{2}$
SLC31A2	$5.71×10^{-17}$	$1.50×10^{-14}$	-2.49	1.28×10	7.35×10
LTBP1	$1.16×10^{-6}$	$3.05×10^{-5}$	-1.25	1.26×10	3.31×10
AACS	$1.15×10^{-12}$	$1.24×10^{-10}$	-1.50	1.25×10	3.53×10
CREB3L4	$9.59×10^{-11}$	$6.96×10^{-9}$	-1.51	1.24×10	3.63×10
FAHD2A	$5.22×10^{-18}$	$1.86×10^{-15}$	-2.06	1.24×10	5.18×10
MEIS1	$1.91×10^{-5}$	$3.23×10^{-4}$	-1.41	1.23×10	3.39×10
ID4	$1.11×10^{-4}$	$1.32×10^{-3}$	-1.75	1.23×10	3.54×10
FGFR2	$1.88×10^{-5}$	$3.20×10^{-4}$	-1.02	1.22×10	2.45×10
SLC12A2	$6.10×10^{-10}$	$3.71×10^{-8}$	-2.19	1.22×10	5.58×10
LIMS2	$4.15×10^{-6}$	$9.28×10^{-5}$	-1.13	1.21×10	2.49×10
LRRC26	$1.98×10^{-16}$	$4.53×10^{-14}$	-3.97	1.21×10	$1.25×10^{2}$
CRYAB	$5.66×10^{-8}$	$2.10×10^{-6}$	-2.20	1.20×10	4.72×10
FGFRL1	$1.65×10^{-9}$	$9.00×10^{-8}$	-1.53	1.20×10	3.42×10

ARHGEF10L	1.06×10^{-10}	7.52×10^{-9}	-1.19	1.18×10	2.71×10
SMR3A	6.06×10^{-6}	1.27×10^{-4}	-4.68	1.16×10	8.79×10
ITGA7	2.67×10^{-13}	3.27×10^{-11}	-1.23	1.15×10	2.69×10
C1orf115	2.51×10^{-5}	4.12×10^{-4}	-1.09	1.14×10	2.48×10
SOX9	1.83×10^{-8}	7.98×10^{-7}	-1.67	1.09×10	3.65×10
GATM	6.15×10^{-19}	2.70×10^{-16}	-2.25	1.08×10	4.62×10
PRR15L	2.41×10^{-3}	1.51×10^{-2}	-1.08	1.08×10	2.16×10
KDELR3	4.04×10^{-6}	9.08×10^{-5}	-1.02	1.08×10	2.23×10
MEIS2	9.47×10^{-6}	1.86×10^{-4}	-1.32	1.07×10	2.71×10
TESC	8.59×10^{-5}	1.07×10^{-3}	-1.67	1.05×10	3.85×10
CNN1	6.52×10^{-14}	8.65×10^{-12}	-3.30	1.05×10	8.92×10
FUT2	1.79×10^{-5}	3.07×10^{-4}	-1.43	1.04×10	2.64×10
OXR1	2.75×10^{-7}	8.53×10^{-6}	-1.36	1.04×10	2.79×10
GGT6	3.94×10^{-5}	5.88×10^{-4}	-1.28	1.04×10	2.28×10
SLC37A2	2.76×10^{-6}	6.50×10^{-5}	-3.05	1.04×10	9.44×10

CEACAM1	4.35×10^{-14}	5.91×10^{-12}	-1.77	1.03×10	3.85×10
C1QTNF1	1.28×10^{-14}	1.93×10^{-12}	-1.30	1.02×10	2.48×10
BSPRY	8.33×10^{-6}	1.67×10^{-4}	-1.59	1.02×10	3.08×10
EPHB3	5.17×10^{-9}	2.54×10^{-7}	-1.75	1.01×10	3.28×10
DAAM2	3.72×10^{-4}	3.37×10^{-3}	-1.32	1.00×10	2.25×10
SVIL	2.39×10^{-8}	1.02×10^{-6}	-1.12	9.98	2.20×10
CYP4X1	2.55×10^{-5}	4.17×10^{-4}	-1.33	9.98	2.32×10
RGMB	1.31×10^{-5}	2.41×10^{-4}	-1.08	9.97	2.12×10
ICA1	4.14×10^{-5}	6.12×10^{-4}	-1.14	9.93	2.19×10
RORC	1.33×10^{-4}	1.53×10^{-3}	-1.59	9.93	2.89×10
GDPD5	1.87×10^{-9}	1.01×10^{-7}	-1.78	9.82	3.48×10
PADI2	2.11×10^{-6}	5.15×10^{-5}	-1.64	9.77	2.65×10
PLIN5	2.68×10^{-7}	8.37×10^{-6}	-2.37	9.75	4.73×10
COL17A1	4.03×10^{-3}	2.26×10^{-2}	-1.16	9.70	1.66×10
FAM3B	1.42×10^{-5}	2.54×10^{-4}	-1.94	9.66	3.65×10

ARHGEF37	2.04×10^{-9}	1.09×10^{-7}	-1.92	9.60	3.59×10
GALNT6	5.62×10^{-18}	1.92×10^{-15}	-2.08	9.55	4.35×10
SNORD74	1.36×10^{-4}	1.55×10^{-3}	-1.29	9.33	2.36×10
RAB26	1.34×10^{-7}	4.50×10^{-6}	-1.24	9.26	2.21×10
CYB5R2	1.89×10^{-5}	3.21×10^{-4}	-1.40	9.18	2.61×10
ST3GAL5	7.01×10^{-10}	4.20×10^{-8}	-1.18	9.17	2.08×10
GYLTL1B	1.58×10^{-4}	1.75×10^{-3}	-1.37	9.15	2.20×10
FAM84B	1.57×10^{-3}	1.07×10^{-2}	-1.08	9.13	1.89×10
TPD52L1	2.52×10^{-7}	7.95×10^{-6}	-2.18	9.13	4.02×10
SMTN	1.62×10^{-11}	1.37×10^{-9}	-1.43	8.99	2.36×10
BARX2	4.57×10^{-11}	3.52×10^{-9}	-2.86	8.86	5.32×10
SUSD2	3.50×10^{-4}	3.23×10^{-3}	-1.07	8.79	1.85×10
GMPR	2.33×10^{-3}	1.46×10^{-2}	-1.15	8.63	1.94×10
AKAP12	3.02×10^{-3}	1.81×10^{-2}	-1.07	8.60	2.41×10
PKP2	1.59×10^{-8}	7.14×10^{-7}	-1.52	8.56	2.51×10

ZNF750	3.64×10^{-4}	3.32×10^{-3}	-1.25	8.32	2.16×10
PIK3AP1	1.48×10^{-6}	3.77×10^{-5}	-1.05	8.28	1.65×10
RAPGEF3	3.46×10^{-5}	5.31×10^{-4}	-1.36	8.21	1.94×10
CCL28	4.95×10^{-12}	4.60×10^{-10}	-3.39	8.10	5.81×10
AGAP11	2.67×10^{-6}	6.30×10^{-5}	-1.64	8.09	2.13×10
PLCB4	9.79×10^{-6}	1.91×10^{-4}	-1.24	8.04	1.81×10
TOM1L1	2.20×10^{-6}	5.32×10^{-5}	-1.48	8.04	2.24×10
ENTPD3	3.67×10^{-4}	3.33×10^{-3}	-1.15	8.01	1.74×10
CHI3L2	3.85×10^{-6}	8.70×10^{-5}	-2.63	7.93	2.52×10
AOC3	7.20×10^{-15}	1.18×10^{-12}	-1.74	7.92	2.56×10
AGFG2	1.69×10^{-10}	1.15×10^{-8}	-1.91	7.84	3.01×10
MIR6717	3.98×10^{-8}	1.57×10^{-6}	-2.90	7.73	4.63×10
DTNB	1.32×10^{-5}	2.41×10^{-4}	-1.25	7.65	1.87×10
FN3K	8.51×10^{-7}	2.33×10^{-5}	-1.07	7.63	1.62×10
FOLR1	3.73×10^{-7}	1.11×10^{-5}	-3.13	7.62	4.98×10

SH3BGRL2	2.60×10^{-7}	8.15×10^{-6}	-1.79	7.60	2.55×10
SLC13A2	7.56×10^{-14}	9.95×10^{-12}	-3.36	7.57	5.89×10
ACTG2	4.59×10^{-16}	9.69×10^{-14}	-4.29	7.41	1.09×10^{2}
FCGBP	1.93×10^{-5}	3.27×10^{-4}	-2.57	7.40	5.60×10
PLAC9	2.01×10^{-3}	1.30×10^{-2}	-1.12	7.33	1.57×10
DNASE1L3	1.42×10^{-4}	1.61×10^{-3}	-1.37	7.31	2.02×10
MAOA	7.66×10^{-5}	9.82×10^{-4}	-1.06	7.31	1.50×10
CASZ1	3.94×10^{-7}	1.17×10^{-5}	-1.13	7.29	1.54×10
FAM174B	4.77×10^{-6}	1.05×10^{-4}	-1.69	7.27	2.27×10
GCNT3	2.58×10^{-10}	1.68×10^{-8}	-2.41	7.21	3.26×10
OGFRL1	5.81×10^{-11}	4.42×10^{-9}	-1.70	7.18	2.44×10
METTL21B	2.91×10^{-10}	1.87×10^{-8}	-1.14	7.17	1.60×10
ATP6V0E2	3.30×10^{-10}	2.09×10^{-8}	-1.79	7.15	2.47×10
WWC1	4.05×10^{-10}	2.55×10^{-8}	-1.57	7.10	2.09×10
PDLIM3	5.77×10^{-13}	6.71×10^{-11}	-1.74	7.07	2.40×10

MUC7	$6.22×10^{-19}$	$2.70×10^{-16}$	-9.76	6.95	$8.78×10^{2}$
TMEM47	$1.72×10^{-6}$	$4.28×10^{-5}$	-1.08	6.93	1.42×10
RBP7	$1.08×10^{-3}$	$7.92×10^{-3}$	-1.08	6.92	1.41×10
SNTA1	$7.05×10^{-7}$	$1.98×10^{-5}$	-1.38	6.89	1.69×10
AIF1L	$1.96×10^{-6}$	$4.83×10^{-5}$	-1.48	6.85	1.88×10
PDK4	$2.21×10^{-4}$	$2.28×10^{-3}$	-1.00	6.85	1.33×10
LIPH	$7.39×10^{-4}$	$5.88×10^{-3}$	-1.10	6.83	1.54×10
SLC44A3	$1.90×10^{-4}$	$2.02×10^{-3}$	-1.00	6.76	1.34×10
FLJ23867	$4.29×10^{-5}$	$6.27×10^{-4}$	-1.25	6.76	1.57×10
RAB17	$5.91×10^{-3}$	$3.07×10^{-2}$	-1.10	6.74	1.29×10
CADPS2	$3.71×10^{-7}$	$1.11×10^{-5}$	-1.32	6.71	1.68×10
SEMA3B	$1.79×10^{-12}$	$1.81×10^{-10}$	-1.52	6.68	1.86×10
B3GNT3	$5.29×10^{-7}$	$1.52×10^{-5}$	-1.68	6.68	1.94×10
COL9A3	$1.80×10^{-4}$	$1.94×10^{-3}$	-1.19	6.58	1.39×10
LRP3	$6.82×10^{-6}$	$1.41×10^{-4}$	-1.05	6.56	1.32×10

LOC101927318	9.98×10^{-4}	7.39×10^{-3}	-1.24	6.56	1.47×10
LINC00342	4.64×10^{-3}	2.52×10^{-2}	-1.11	6.55	1.17×10
TACC2	1.50×10^{-3}	1.04×10^{-2}	-1.05	6.53	1.30×10
SEZ6L2	5.42×10^{-4}	4.58×10^{-3}	-1.04	6.51	1.41×10
FAM46A	1.57×10^{-8}	7.04×10^{-7}	-1.64	6.51	2.17×10
CHMP4C	8.97×10^{-3}	4.21×10^{-2}	-1.05	6.45	1.31×10
LMF1	1.96×10^{-13}	2.46×10^{-11}	-1.65	6.45	1.96×10
SLC5A1	5.51×10^{-12}	5.02×10^{-10}	-3.17	6.44	4.39×10
PDE2A	7.83×10^{-4}	6.15×10^{-3}	-1.18	6.32	1.25×10
PRB1	3.28×10^{-18}	1.20×10^{-15}	-12.1	6.32	9.87×10^{3}
LIMCH1	1.33×10^{-9}	7.46×10^{-8}	-1.61	6.32	1.84×10
TBC1D16	5.24×10^{-8}	1.98×10^{-6}	-1.07	6.28	1.30×10
RAVER2	2.26×10^{-9}	1.20×10^{-7}	-1.40	6.28	1.66×10
CCDC149	2.80×10^{-8}	1.16×10^{-6}	-1.42	6.26	1.71×10
PRKAR2B	2.28×10^{-5}	3.78×10^{-4}	-1.02	6.21	1.24×10

PDGFC	3.37×10^{-5}	5.20×10^{-4}	-1.19	6.21	1.44×10
C1orf116	1.77×10^{-7}	5.75×10^{-6}	-1.10	6.20	1.28×10
ICK	1.80×10^{-8}	7.93×10^{-7}	-1.16	6.17	1.39×10
NPNT	9.38×10^{-4}	7.01×10^{-3}	-1.49	6.06	1.13×10
RAP1GAP2	5.51×10^{-9}	2.70×10^{-7}	-1.10	6.01	1.31×10
CAB39L	1.22×10^{-5}	2.26×10^{-4}	-1.25	5.98	1.45×10
PLA2R1	2.75×10^{-7}	8.53×10^{-6}	-1.17	5.98	1.45×10
TTYH1	6.32×10^{-3}	3.23×10^{-2}	-1.56	5.95	1.32×10
C9orf152	1.30×10^{-9}	7.28×10^{-8}	-1.75	5.92	1.80×10
SOX10	5.81×10^{-13}	6.71×10^{-11}	-3.94	5.89	6.15×10
TCEAL2	1.66×10^{-6}	4.14×10^{-5}	-1.54	5.88	1.69×10
PKDCC	2.27×10^{-9}	1.20×10^{-7}	-3.84	5.77	9.33×10
TGFBR3	3.04×10^{-6}	7.10×10^{-5}	-1.35	5.69	1.29×10
LURAP1L	6.85×10^{-3}	3.43×10^{-2}	-1.06	5.67	1.15×10
GPT	1.13×10^{-9}	6.52×10^{-8}	-2.20	5.52	2.42×10

SORBS1	3.16×10^{-34}	7.24×10^{-31}	-2.41	5.50	2.81×10
ALDH1L1	2.05×10^{-13}	2.55×10^{-11}	-3.05	5.49	3.85×10
ABCA8	2.93×10^{-4}	2.81×10^{-3}	-1.47	5.46	1.32×10
CAMSAP3	8.34×10^{-7}	2.30×10^{-5}	-1.53	5.44	1.54×10
NOSTRIN	4.20×10^{-4}	3.72×10^{-3}	-1.15	5.44	1.18×10
SEC16B	7.54×10^{-6}	1.53×10^{-4}	-1.00	5.42	1.04×10
NPY1R	1.01×10^{-12}	1.11×10^{-10}	-2.13	5.41	2.26×10
FXYD1	3.38×10^{-5}	5.21×10^{-4}	-1.48	5.34	1.37×10
DEPTOR	8.53×10^{-16}	1.73×10^{-13}	-2.41	5.31	2.61×10
SOCS6	5.00×10^{-5}	7.06×10^{-4}	-1.12	5.29	1.22×10
PODXL2	1.05×10^{-12}	1.14×10^{-10}	-1.92	5.23	2.02×10
SLITRK6	9.43×10^{-5}	1.16×10^{-3}	-1.26	5.23	1.20×10
PC	2.07×10^{-7}	6.61×10^{-6}	-1.52	5.21	1.49×10
CA2	5.05×10^{-17}	1.35×10^{-14}	-4.71	5.20	1.22×10^{2}
PNPLA7	1.91×10^{-15}	3.52×10^{-13}	-2.05	5.18	2.05×10

TF	3.90×10^{-4}	3.49×10^{-3}	-1.68	5.14	1.52×10
NCALD	3.47×10^{-8}	1.40×10^{-6}	-2.29	5.13	2.71×10
FUT6	1.39×10^{-7}	4.61×10^{-6}	-2.15	5.08	1.91×10
RASL12	1.01×10^{-10}	7.28×10^{-9}	-1.77	5.08	1.71×10
VIPR1	1.22×10^{-8}	5.56×10^{-7}	-1.63	5.07	1.49×10
CRISP3	1.10×10^{-15}	2.18×10^{-13}	-10.7	5.07	5.98×10^{2}
DOPEY2	1.29×10^{-10}	9.06×10^{-9}	-1.04	5.06	1.07×10
PTGIS	1.47×10^{-3}	1.02×10^{-2}	-1.11	4.98	1.03×10
ITGA10	7.90×10^{-5}	1.00×10^{-3}	-1.31	4.95	1.13×10
ITIH4	6.04×10^{-8}	2.23×10^{-6}	-1.02	4.91	1.00×10
FBXL8	1.07×10^{-5}	2.04×10^{-4}	-1.05	4.90	1.02×10
CBR3	2.66×10^{-10}	1.72×10^{-8}	-2.41	4.89	2.59×10
NEURL3	6.98×10^{-10}	4.20×10^{-8}	-2.66	4.87	2.53×10
SLC43A1	7.19×10^{-6}	1.47×10^{-4}	-1.34	4.86	1.20×10
PLXNB3	2.39×10^{-12}	2.35×10^{-10}	-2.25	4.85	2.10×10

LDHD	4.04×10^{-9}	2.05×10^{-7}	-1.48	4.82	1.32×10
PPP1R3C	3.13×10^{-4}	2.96×10^{-3}	-1.53	4.76	1.46×10
HSD11B2	4.86×10^{-6}	1.07×10^{-4}	-1.93	4.75	1.51×10
RAP1GAP	1.87×10^{-18}	7.33×10^{-16}	-3.19	4.72	3.94×10
SLC2A9	1.89×10^{-7}	6.08×10^{-6}	-1.38	4.56	1.20×10
CHDH	3.83×10^{-13}	4.59×10^{-11}	-1.78	4.54	1.62×10
CHRDL1	2.42×10^{-20}	1.25×10^{-17}	-2.05	4.49	1.68×10
LOC102723505	3.87×10^{-3}	2.19×10^{-2}	-1.25	4.46	1.29×10
FZD7	1.53×10^{-5}	2.70×10^{-4}	-1.03	4.40	8.77
TBX1	3.85×10^{-5}	5.79×10^{-4}	-1.04	4.35	8.79
ST3GAL6	3.97×10^{-9}	2.02×10^{-7}	-1.54	4.32	1.31×10
CAMKK1	9.91×10^{-9}	4.60×10^{-7}	-1.29	4.31	1.06×10
DUSP4	1.89×10^{-14}	2.78×10^{-12}	-2.13	4.29	2.05×10
DNAJB5	2.66×10^{-4}	2.62×10^{-3}	-1.06	4.29	8.90
SMAD9	2.13×10^{-3}	1.36×10^{-2}	-1.15	4.25	8.15

CES3	$3.65×10^{-7}$	$1.09×10^{-5}$	-2.22	4.22	1.58×10
CRACR2A	$3.58×10^{-13}$	$4.32×10^{-11}$	-1.76	4.21	1.47×10
RASEF	$1.00×10^{-3}$	$7.43×10^{-3}$	-1.41	4.19	1.15×10
CGNL1	$3.05×10^{-15}$	$5.39×10^{-13}$	-2.62	4.19	2.40×10
CST4	$1.74×10^{-7}$	$5.67×10^{-6}$	-4.83	4.18	9.03×10
SHF	$4.10×10^{-4}$	$3.64×10^{-3}$	-1.08	4.18	8.55
MIR3142HG	$6.29×10^{-5}$	$8.39×10^{-4}$	-1.65	4.17	1.16×10
PGM5	$4.39×10^{-4}$	$3.85×10^{-3}$	-1.53	4.12	9.94
FLNC	$2.04×10^{-4}$	$2.15×10^{-3}$	-1.33	4.10	8.87
PAIP2B	$7.35×10^{-7}$	$2.05×10^{-5}$	-1.49	4.09	1.14×10
TMPRSS5	$1.75×10^{-7}$	$5.69×10^{-6}$	-2.13	4.07	1.17×10
ALDH3B2	$5.02×10^{-3}$	$2.68×10^{-2}$	-1.13	4.06	7.65
LOC102723344	$2.61×10^{-6}$	$6.16×10^{-5}$	-2.87	4.06	2.11×10
DPT	$2.07×10^{-4}$	$2.17×10^{-3}$	-1.53	4.06	1.08×10
LRP6	$4.31×10^{-6}$	$9.54×10^{-5}$	-1.09	4.05	8.67

BCKDHB	1.32×10^{-7}	4.41×10^{-6}	-1.15	3.99	9.09
NEXN	5.47×10^{-5}	7.57×10^{-4}	-1.41	3.98	9.62
LOC102724344	3.93×10^{-8}	1.55×10^{-6}	-2.49	3.89	1.68×10
CAPN12	5.99×10^{-6}	1.26×10^{-4}	-1.08	3.83	7.86
ANKDD1A	7.93×10^{-7}	2.19×10^{-5}	-1.05	3.81	7.61
MARC1	1.44×10^{-3}	9.96×10^{-3}	-1.06	3.80	7.83
LOC102723517	4.13×10^{-6}	9.24×10^{-5}	-1.73	3.79	1.28×10
SLC28A3	7.46×10^{-3}	3.67×10^{-2}	-1.23	3.77	1.12×10
CBS	9.08×10^{-10}	5.30×10^{-8}	-3.22	3.74	3.20×10
RTN1	2.28×10^{-6}	5.49×10^{-5}	-1.68	3.74	1.17×10
VIPR1-AS1	1.80×10^{-4}	1.94×10^{-3}	-1.52	3.72	1.02×10
F5	4.39×10^{-11}	3.41×10^{-9}	-1.80	3.72	1.15×10
ANG	1.14×10^{-6}	3.02×10^{-5}	-1.67	3.68	1.19×10
LOC283335	2.74×10^{-8}	1.13×10^{-6}	-1.35	3.67	8.74
PRSS16	3.53×10^{-6}	8.06×10^{-5}	-1.12	3.67	7.72

KLF15	7.74×10^{-10}	4.56×10^{-8}	-2.23	3.66	1.63×10
NUDT8	2.99×10^{-4}	2.85×10^{-3}	-1.08	3.65	7.94
FHOD3	2.69×10^{-8}	1.12×10^{-6}	-1.29	3.62	8.97
CARMN	1.64×10^{-6}	4.11×10^{-5}	-1.79	3.55	1.23×10
PDE1B	9.21×10^{-4}	6.92×10^{-3}	-1.07	3.48	6.89
ADCY5	8.00×10^{-15}	1.30×10^{-12}	-2.38	3.42	1.72×10
HSPB6	2.59×10^{-8}	1.09×10^{-6}	-2.19	3.40	1.35×10
PCP2	1.37×10^{-4}	1.57×10^{-3}	-1.11	3.34	7.47
BTNL9	1.11×10^{-8}	5.07×10^{-7}	-1.56	3.32	9.30
BEND7	3.80×10^{-4}	3.42×10^{-3}	-1.37	3.28	8.20
LOC101928307	8.25×10^{-5}	1.04×10^{-3}	-1.41	3.28	7.62
NTRK3	3.10×10^{-3}	1.84×10^{-2}	-1.59	3.27	7.64
PAX1	5.11×10^{-4}	4.36×10^{-3}	-2.67	3.23	9.69
C4orf19	2.68×10^{-4}	2.63×10^{-3}	-1.45	3.20	8.00
GLYATL2	1.61×10^{-4}	1.77×10^{-3}	-2.25	3.17	1.13×10

SLC52A3	1.14×10^{-3}	8.24×10^{-3}	-1.27	3.15	8.88
DPP4	1.44×10^{-12}	1.51×10^{-10}	-1.72	3.14	1.12×10
PTCH2	2.27×10^{-17}	6.49×10^{-15}	-2.79	3.13	2.12×10
FAM46B	3.36×10^{-15}	5.79×10^{-13}	-2.26	3.13	1.44×10
NFASC	9.12×10^{-11}	6.65×10^{-9}	-1.24	3.11	7.32
S100B	1.45×10^{-6}	3.70×10^{-5}	-1.66	3.10	1.03×10
MELTF	3.54×10^{-7}	1.06×10^{-5}	-1.64	3.08	9.55
CSPG4	6.26×10^{-7}	1.77×10^{-5}	-1.10	3.07	6.34
TBC1D24	3.25×10^{-15}	5.66×10^{-13}	-1.44	3.05	8.33
CAPN8	3.96×10^{-7}	1.17×10^{-5}	-1.79	3.05	1.03×10
CRLF1	5.73×10^{-5}	7.81×10^{-4}	-2.36	3.05	1.45×10
ARFGEF3	4.29×10^{-3}	2.38×10^{-2}	-1.22	3.03	6.82
DACT3	5.82×10^{-6}	1.23×10^{-4}	-1.94	3.02	1.09×10
WNK2	1.62×10^{-16}	3.77×10^{-14}	-2.22	3.01	1.35×10
LGI4	6.42×10^{-18}	2.10×10^{-15}	-2.22	3.01	1.33×10

TTC39A	2.86×10^{-5}	4.57×10^{-4}	-1.26	2.98	6.74
LEPR	2.27×10^{-4}	2.32×10^{-3}	-1.11	2.95	6.49
SLC26A10	2.94×10^{-4}	2.82×10^{-3}	-1.45	2.94	8.27
SNPH	2.75×10^{-9}	1.43×10^{-7}	-1.75	2.94	9.43
BEX5	1.31×10^{-4}	1.51×10^{-3}	-1.21	2.90	6.54
COL28A1	8.37×10^{-8}	2.99×10^{-6}	-2.30	2.87	1.21×10
GJB1	8.17×10^{-8}	2.93×10^{-6}	-1.93	2.85	1.03×10
OSR1	9.80×10^{-6}	1.91×10^{-4}	-2.12	2.84	9.01
PRSS21	7.52×10^{-3}	3.69×10^{-2}	-1.52	2.83	7.81
COBL	5.20×10^{-8}	1.97×10^{-6}	-1.98	2.80	9.83
PPP1R14A	4.14×10^{-12}	3.91×10^{-10}	-1.85	2.79	9.46
HSPB2	6.50×10^{-3}	3.30×10^{-2}	-1.02	2.79	5.53
DACT1	2.54×10^{-4}	2.54×10^{-3}	-1.48	2.79	6.75
SYT7	9.73×10^{-10}	5.64×10^{-8}	-2.29	2.78	1.17×10
SPEG	2.04×10^{-11}	1.69×10^{-9}	-2.29	2.78	1.25×10

MSX1	7.76×10^{-6}	1.57×10^{-4}	-1.49	2.77	7.30
ABCA3	1.19×10^{-11}	1.04×10^{-9}	-1.75	2.76	9.03
NCMAP	1.87×10^{-3}	1.23×10^{-2}	-1.11	2.75	6.11
SMOC1	9.23×10^{-5}	1.14×10^{-3}	-1.65	2.75	7.53
GATA3	2.06×10^{-5}	3.46×10^{-4}	-1.12	2.74	6.24
FAHD2CP	1.00×10^{-9}	5.78×10^{-8}	-1.58	2.73	8.13
NDRG4	7.15×10^{-11}	5.36×10^{-9}	-1.17	2.71	6.16
ADAMTSL1	1.78×10^{-5}	3.06×10^{-4}	-1.93	2.68	1.03×10
PRELP	1.23×10^{-13}	1.59×10^{-11}	-1.89	2.66	9.13
CACNA1H	1.12×10^{-14}	1.72×10^{-12}	-1.42	2.63	7.14
ADGRG6	2.05×10^{-10}	1.37×10^{-8}	-2.68	2.57	1.41×10
ARHGAP5-AS1	1.72×10^{-3}	1.15×10^{-2}	-1.04	2.51	4.94
PRB2	3.39×10^{-11}	2.68×10^{-9}	-8.38	2.50	1.64×10^{3}
XDH	3.57×10^{-4}	3.27×10^{-3}	-1.80	2.46	7.62
AK4	6.45×10^{-5}	8.56×10^{-4}	-1.30	2.45	6.23

LOC102723927	1.32×10^{-5}	2.41×10^{-4}	-1.39	2.45	5.98
RBM24	3.13×10^{-5}	4.91×10^{-4}	-1.27	2.44	6.06
MKRN2OS	6.52×10^{-3}	3.31×10^{-2}	-1.03	2.43	4.66
PLIN4	2.34×10^{-6}	5.60×10^{-5}	-1.83	2.43	7.99
MAMDC2	7.65×10^{-3}	3.73×10^{-2}	-1.09	2.43	3.78
DMD	1.87×10^{-8}	8.09×10^{-7}	-1.08	2.42	5.16
ZBTB16	5.63×10^{-5}	7.73×10^{-4}	-1.83	2.39	7.45
EFNA3	1.70×10^{-4}	1.85×10^{-3}	-1.34	2.34	6.01
GARNL3	2.44×10^{-6}	5.83×10^{-5}	-1.66	2.32	6.79
TMEM139	6.37×10^{-4}	5.21×10^{-3}	-1.14	2.30	4.78
C2orf88	2.87×10^{-6}	6.73×10^{-5}	-1.09	2.30	4.71
RBPMS2	1.04×10^{-10}	7.43×10^{-9}	-2.03	2.28	8.43
ABTB2	4.60×10^{-9}	2.30×10^{-7}	-1.18	2.26	5.17
GSTM5	5.86×10^{-3}	3.05×10^{-2}	-1.40	2.25	4.19
SHANK2	3.63×10^{-4}	3.31×10^{-3}	-1.18	2.25	4.89

POM121L9P	3.96×10^{-3}	2.23×10^{-2}	-1.11	2.24	5.63
CCDC85A	4.05×10^{-6}	9.09×10^{-5}	-1.12	2.24	4.80
MN1	7.67×10^{-3}	3.74×10^{-2}	-1.16	2.23	4.26
APBA1	9.32×10^{-4}	6.99×10^{-3}	-1.06	2.22	4.34
GIPC3	3.58×10^{-8}	1.44×10^{-6}	-1.30	2.21	5.49
PDE5A	1.48×10^{-10}	1.02×10^{-8}	-1.43	2.20	6.20
PAX7	2.85×10^{-3}	1.73×10^{-2}	-1.86	2.17	6.76
PCSK1N	4.46×10^{-4}	3.90×10^{-3}	-1.70	2.15	7.31
CDO1	4.92×10^{-4}	4.23×10^{-3}	-1.76	2.14	4.69
UGT8	1.92×10^{-5}	3.25×10^{-4}	-1.10	2.12	4.63
NKX3-1	5.79×10^{-12}	5.22×10^{-10}	-3.83	2.09	3.21×10
LPO	2.92×10^{-18}	1.09×10^{-15}	-8.83	2.08	1.83×10^{2}
MYRIP	4.22×10^{-10}	2.64×10^{-8}	-1.89	2.05	6.88
C15orf65	4.11×10^{-4}	3.65×10^{-3}	-1.11	2.05	4.30
SRCIN1	1.36×10^{-3}	9.52×10^{-3}	-1.34	2.05	4.56

ATP10A	8.77×10^{-12}	7.77×10^{-10}	-1.45	2.04	5.62
PTGER3	7.37×10^{-5}	9.52×10^{-4}	-1.41	2.04	4.77
ANKRD36B	1.60×10^{-4}	1.77×10^{-3}	-1.02	2.03	4.22
RGN	4.96×10^{-6}	1.08×10^{-4}	-1.36	2.00	4.65
KCNIP2	1.17×10^{-9}	6.70×10^{-8}	-1.21	1.99	4.56
C3orf70	1.31×10^{-5}	2.41×10^{-4}	-1.68	1.98	4.96
HSPB7	3.63×10^{-11}	2.86×10^{-9}	-2.27	1.95	8.31
TNFRSF11A	4.86×10^{-24}	4.11×10^{-21}	-2.59	1.95	1.15×10
SYNM	4.33×10^{-38}	1.74×10^{-34}	-2.74	1.94	1.33×10
CEP85L	3.05×10^{-6}	7.10×10^{-5}	-1.22	1.94	4.59
ADGRB3	2.90×10^{-4}	2.80×10^{-3}	-1.21	1.94	3.85
B3GNT6	2.94×10^{-12}	2.83×10^{-10}	-2.91	1.92	1.10×10
CEP41	8.79×10^{-11}	6.47×10^{-9}	-1.72	1.91	6.45
RASAL1	1.61×10^{-14}	2.39×10^{-12}	-3.89	1.90	1.86×10
ZBED3	5.48×10^{-5}	7.57×10^{-4}	-1.07	1.89	3.95

GPAT2	1.16×10^{-7}	3.94×10^{-6}	-2.08	1.89	7.01
TMEM211	7.23×10^{-7}	2.02×10^{-5}	-1.88	1.88	6.85
PLN	2.88×10^{-19}	1.32×10^{-16}	-3.46	1.86	1.76×10
RNF152	7.36×10^{-3}	3.62×10^{-2}	-1.06	1.81	4.17
DMRTA1	2.94×10^{-4}	2.82×10^{-3}	-2.05	1.80	6.20
LOC101929719	3.40×10^{-3}	1.99×10^{-2}	-1.06	1.77	3.78
BAIAP2L2	5.77×10^{-5}	7.84×10^{-4}	-1.21	1.77	4.08
DIO2	3.57×10^{-9}	1.84×10^{-7}	-1.86	1.76	6.17
NOXO1	7.62×10^{-7}	2.12×10^{-5}	-1.83	1.76	5.42
HR	7.45×10^{-19}	3.14×10^{-16}	-2.33	1.76	8.23
SLAIN1	4.16×10^{-8}	1.63×10^{-6}	-1.94	1.76	7.50
PTX3	8.77×10^{-3}	4.13×10^{-2}	-1.36	1.73	3.93
RASD2	7.66×10^{-7}	2.13×10^{-5}	-1.50	1.72	4.17
KCNMB1	1.13×10^{-33}	2.26×10^{-30}	-2.99	1.72	1.33×10
HSPA12A	2.16×10^{-16}	4.87×10^{-14}	-1.48	1.70	4.78

GPR17	3.50×10^{-6}	8.02×10^{-5}	-1.56	1.68	4.44
FAM171B	8.75×10^{-4}	6.65×10^{-3}	-1.31	1.67	3.96
HIF3A	7.26×10^{-6}	1.48×10^{-4}	-2.84	1.65	7.90
RAMP2-AS1	2.63×10^{-3}	1.62×10^{-2}	-1.04	1.65	2.88
IRX5	1.48×10^{-4}	1.65×10^{-3}	-1.95	1.63	4.55
GPM6A	1.57×10^{-3}	1.07×10^{-2}	-1.87	1.62	2.90
FABP4	1.82×10^{-11}	1.53×10^{-9}	-2.13	1.60	6.31
SCUBE3	1.06×10^{-5}	2.03×10^{-4}	-1.27	1.59	3.72
TLN2	1.28×10^{-3}	9.08×10^{-3}	-1.11	1.59	3.09
TTLL7	1.89×10^{-9}	1.02×10^{-7}	-1.42	1.58	4.40
DBNDD1	1.12×10^{-6}	2.96×10^{-5}	-1.54	1.57	4.71
TYRO3	1.39×10^{-5}	2.51×10^{-4}	-1.28	1.57	3.76
CPED1	4.32×10^{-8}	1.68×10^{-6}	-1.70	1.56	5.00
KIRREL3	1.86×10^{-3}	1.22×10^{-2}	-1.38	1.55	3.25
CASQ2	4.30×10^{-7}	1.26×10^{-5}	-2.33	1.54	6.55

KIAA1755	5.66×10^{-7}	1.62×10^{-5}	-1.96	1.53	5.29
KCNMA1	2.82×10^{-22}	1.88×10^{-19}	-2.42	1.53	8.07
CBR3-AS1	7.79×10^{-9}	3.73×10^{-7}	-2.14	1.52	6.84
ADH1B	1.88×10^{-11}	1.57×10^{-9}	-2.08	1.51	5.58
NRTN	2.69×10^{-8}	1.12×10^{-6}	-1.63	1.51	4.74
CYP4F24P	6.56×10^{-3}	3.32×10^{-2}	-1.11	1.49	2.83
OVOL1	1.34×10^{-4}	1.53×10^{-3}	-1.78	1.49	5.08
MEOX2	1.36×10^{-4}	1.55×10^{-3}	-1.05	1.47	2.96
INPP5J	1.64×10^{-8}	7.28×10^{-7}	-1.76	1.46	4.80
SOSTDC1	1.05×10^{-5}	2.01×10^{-4}	-1.88	1.46	4.82
GALNT15	3.16×10^{-3}	1.87×10^{-2}	-1.19	1.46	2.77
RAB27B	1.04×10^{-10}	7.43×10^{-9}	-1.41	1.45	3.97
KANK4	2.68×10^{-7}	8.37×10^{-6}	-2.60	1.44	6.84
MPZ	2.54×10^{-11}	2.06×10^{-9}	-2.87	1.44	1.13×10
RNF180	2.72×10^{-4}	2.67×10^{-3}	-1.05	1.43	2.87

LNP1	$1.64×10^{-8}$	$7.28×10^{-7}$	-1.32	1.43	3.51
KAZALD1	$1.04×10^{-27}$	$1.05×10^{-24}$	-2.35	1.42	7.07
RTN4RL1	$4.14×10^{-4}$	$3.67×10^{-3}$	-1.42	1.42	3.84
SLC22A3	$1.04×10^{-17}$	$3.20×10^{-15}$	-4.03	1.41	2.08×10
PLCB1	$1.58×10^{-6}$	$3.97×10^{-5}$	-1.32	1.41	3.42
CA3	$5.41×10^{-7}$	$1.55×10^{-5}$	-2.41	1.40	7.00
MYCN	$9.12×10^{-4}$	$6.87×10^{-3}$	-1.60	1.38	3.73
GRIP1	$3.07×10^{-4}$	$2.92×10^{-3}$	-1.29	1.37	3.09
PRB4	$4.44×10^{-17}$	$1.21×10^{-14}$	-12.0	1.37	$5.16×10^{3}$
GNAZ	$6.20×10^{-4}$	$5.11×10^{-3}$	-1.04	1.37	2.75
ACTA2-AS1	$4.55×10^{-10}$	$2.81×10^{-8}$	-2.95	1.37	7.82
LRRN2	$2.60×10^{-6}$	$6.15×10^{-5}$	-1.37	1.36	3.59
WIPF3	$2.89×10^{-6}$	$6.75×10^{-5}$	-1.49	1.35	3.64
WBSCR17	$1.80×10^{-6}$	$4.48×10^{-5}$	-2.62	1.33	7.68
FAM177B	$1.71×10^{-5}$	$2.97×10^{-4}$	-1.58	1.32	3.90

PLP1	6.25×10^{-6}	1.31×10^{-4}	-3.20	1.31	9.41
AATK	4.73×10^{-4}	4.09×10^{-3}	-1.26	1.30	2.99
CCDC68	1.52×10^{-3}	1.05×10^{-2}	-1.37	1.29	3.03
GLI2	9.60×10^{-5}	1.17×10^{-3}	-1.22	1.29	2.86
PPP2R3A	4.95×10^{-6}	1.08×10^{-4}	-1.18	1.29	2.99
FOXC2	4.22×10^{-4}	3.73×10^{-3}	-2.14	1.27	3.62
ITIH3	9.53×10^{-18}	3.00×10^{-15}	-3.07	1.27	9.68
KIAA1324L	2.79×10^{-5}	4.48×10^{-4}	-1.24	1.27	2.83
PYGM	2.30×10^{-14}	3.35×10^{-12}	-1.67	1.24	3.90
LDB3	1.19×10^{-12}	1.27×10^{-10}	-2.52	1.24	6.21
FNDC5	3.47×10^{-3}	2.02×10^{-2}	-1.02	1.23	2.10
CLDN8	3.50×10^{-3}	2.03×10^{-2}	-1.19	1.22	2.54
MGAM2	3.15×10^{-10}	2.00×10^{-8}	-2.86	1.20	7.79
TTR	2.53×10^{-4}	2.53×10^{-3}	-2.52	1.19	5.42
FOLH1	6.80×10^{-17}	1.71×10^{-14}	-3.84	1.18	1.60×10

SLC16A8	7.01×10^{-10}	4.20×10^{-8}	-1.86	1.18	4.13
TRIL	3.87×10^{-5}	5.80×10^{-4}	-1.70	1.18	3.64
IRX2	1.78×10^{-15}	3.36×10^{-13}	-3.31	1.18	9.93
SOX8	6.26×10^{-13}	7.18×10^{-11}	-2.80	1.17	7.17
MYOM1	3.29×10^{-7}	1.00×10^{-5}	-2.12	1.17	4.50
RANBP17	2.47×10^{-6}	5.88×10^{-5}	-1.37	1.16	2.98
TBC1D30	1.11×10^{-6}	2.93×10^{-5}	-1.94	1.16	5.05
GPD1	2.16×10^{-10}	1.43×10^{-8}	-3.21	1.14	1.09×10
ABCA10	1.22×10^{-9}	6.93×10^{-8}	-1.78	1.13	3.96
DES	6.41×10^{-52}	1.03×10^{-47}	-7.93	1.13	2.15×10^{2}
HMGCS2	2.67×10^{-14}	3.82×10^{-12}	-5.29	1.12	2.88×10
LINC00920	3.60×10^{-8}	1.44×10^{-6}	-1.61	1.12	3.40
SLC2A4	1.68×10^{-9}	9.12×10^{-8}	-1.82	1.12	3.91
TPTEP1	2.11×10^{-7}	6.72×10^{-6}	-2.97	1.09	7.03
DSCC1	2.12×10^{-4}	2.21×10^{-3}	-1.03	1.08	2.19

COLGALT2	$3.80×10^{-8}$	$1.51×10^{-6}$	-2.37	1.07	5.03
DENND2A	$9.36×10^{-9}$	$4.39×10^{-7}$	-1.28	1.06	2.55
RNF128	$1.40×10^{-10}$	$9.74×10^{-9}$	-3.26	1.05	8.57
CCDC129	$3.73×10^{-9}$	$1.91×10^{-7}$	-3.66	1.05	8.41
OXGR1	$6.88×10^{-10}$	$4.16×10^{-8}$	-2.94	1.04	6.99
CNTFR	$2.85×10^{-15}$	$5.07×10^{-13}$	-3.92	1.04	1.25×10
NDNF	$1.10×10^{-5}$	$2.09×10^{-4}$	-1.65	1.02	2.71
C6orf141	$7.20×10^{-5}$	$9.37×10^{-4}$	-2.16	1.01	2.69
C2orf82	$3.68×10^{-9}$	$1.89×10^{-7}$	-2.65	1.01	8.68
GFRA3	$1.82×10^{-7}$	$5.90×10^{-6}$	-2.80	1.01	5.47
CRISP2	$5.38×10^{-11}$	$4.11×10^{-9}$	-5.48	1.00	2.33×10
DKK1	$6.69×10^{-3}$	$3.37×10^{-2}$	-1.66	$9.98×10^{-1}$	5.83
LAMA2	$4.94×10^{-5}$	$6.98×10^{-4}$	-1.18	$9.96×10^{-1}$	2.25
SGCA	$1.30×10^{-14}$	$1.95×10^{-12}$	-2.70	$9.93×10^{-1}$	5.78
ADGRL3	$9.38×10^{-4}$	$7.01×10^{-3}$	-1.24	$9.90×10^{-1}$	2.15

KCNAB1	$5.18{\times}10^{-7}$	$1.50{\times}10^{-5}$	-1.06	$9.87{\times}10^{-1}$	2.05
ADRA2B	$7.90{\times}10^{-6}$	$1.60{\times}10^{-4}$	-2.07	$9.84{\times}10^{-1}$	4.07
RTN4RL2	$4.33{\times}10^{-5}$	$6.30{\times}10^{-4}$	-1.22	$9.83{\times}10^{-1}$	2.28
KCNQ4	$1.85{\times}10^{-15}$	$3.45{\times}10^{-13}$	-2.13	$9.82{\times}10^{-1}$	4.28
NAALADL2	$1.20{\times}10^{-6}$	$3.14{\times}10^{-5}$	-1.34	$9.77{\times}10^{-1}$	2.44
GPRC5D	$4.96{\times}10^{-3}$	$2.66{\times}10^{-2}$	-1.20	$9.66{\times}10^{-1}$	2.33
AGT	$1.25{\times}10^{-4}$	$1.45{\times}10^{-3}$	-1.48	$9.58{\times}10^{-1}$	2.41
PRB3	$3.16{\times}10^{-46}$	$1.69{\times}10^{-42}$	-11.2	$9.56{\times}10^{-1}$	$4.87{\times}10^{3}$
C1orf95	$9.19{\times}10^{-7}$	$2.49{\times}10^{-5}$	-1.66	$9.51{\times}10^{-1}$	2.83
ADCY1	$5.22{\times}10^{-6}$	$1.13{\times}10^{-4}$	-1.23	$9.41{\times}10^{-1}$	2.07
TMEM35	$1.50{\times}10^{-9}$	$8.26{\times}10^{-8}$	-2.14	$9.35{\times}10^{-1}$	4.06
RAD51B	$1.10{\times}10^{-7}$	$3.77{\times}10^{-6}$	-1.45	$9.33{\times}10^{-1}$	2.61
PRAP1	$2.76{\times}10^{-4}$	$2.70{\times}10^{-3}$	-1.25	$9.25{\times}10^{-1}$	2.18
CILP	$5.92{\times}10^{-7}$	$1.69{\times}10^{-5}$	-1.26	$9.24{\times}10^{-1}$	2.27
AR	$4.22{\times}10^{-5}$	$6.21{\times}10^{-4}$	-1.19	$9.17{\times}10^{-1}$	2.10

RGMB-AS1	$5.39×10^{-6}$	$1.16×10^{-4}$	-1.97	$9.17×10^{-1}$	3.31
PPP1R9A	$3.59×10^{-6}$	$8.19×10^{-5}$	-1.57	$9.13×10^{-1}$	2.77
L1CAM	$1.30×10^{-22}$	$9.06×10^{-20}$	-2.82	$9.05×10^{-1}$	6.15
LINC01197	$2.38×10^{-4}$	$2.41×10^{-3}$	-1.14	$9.03×10^{-1}$	1.88
TMEM56	$1.99×10^{-8}$	$8.57×10^{-7}$	-1.47	$9.02×10^{-1}$	2.49
MYOZ1	$5.29×10^{-6}$	$1.14×10^{-4}$	-1.53	$8.93×10^{-1}$	2.61
ADAMTS17	$5.87×10^{-10}$	$3.58×10^{-8}$	-1.70	$8.83×10^{-1}$	2.82
GRIK2	$2.62×10^{-3}$	$1.61×10^{-2}$	-1.22	$8.70×10^{-1}$	2.04
PRDM16	$6.80×10^{-3}$	$3.41×10^{-2}$	-1.38	$8.60×10^{-1}$	1.54
DOCK3	$7.28×10^{-10}$	$4.33×10^{-8}$	-2.16	$8.46×10^{-1}$	3.43
SLCO1A2	$4.48×10^{-5}$	$6.47×10^{-4}$	-3.50	$8.40×10^{-1}$	3.05
C3orf35	$8.26×10^{-4}$	$6.39×10^{-3}$	-1.09	$8.35×10^{-1}$	1.82
NXPH3	$2.30×10^{-4}$	$2.35×10^{-3}$	-1.70	$8.34×10^{-1}$	2.53
CLGN	$8.74×10^{-3}$	$4.12×10^{-2}$	-1.13	$8.30×10^{-1}$	1.50
CCDC110	$3.53×10^{-6}$	$8.06×10^{-5}$	-1.06	$8.28×10^{-1}$	1.75

PRRG3	9.22×10^{-9}	4.34×10^{-7}	-2.10	8.24×10^{-1}	3.13
FAM19A5	1.20×10^{-5}	2.23×10^{-4}	-1.50	8.23×10^{-1}	2.31
PON3	6.88×10^{-8}	2.52×10^{-6}	-1.95	8.23×10^{-1}	3.08
HRCT1	1.76×10^{-5}	3.04×10^{-4}	-1.68	8.22×10^{-1}	2.66
ASPA	2.09×10^{-4}	2.19×10^{-3}	-1.67	8.20×10^{-1}	1.84
KIF12	1.25×10^{-3}	8.94×10^{-3}	-1.12	8.20×10^{-1}	1.59
FAM180A	2.55×10^{-4}	2.54×10^{-3}	-1.83	8.14×10^{-1}	2.47
GDF10	1.01×10^{-6}	2.70×10^{-5}	-3.06	8.01×10^{-1}	6.78
VIPR2	7.28×10^{-4}	5.82×10^{-3}	-1.13	7.95×10^{-1}	1.81
SMR3B	2.60×10^{-15}	4.68×10^{-13}	-5.75	7.95×10^{-1}	4.03×10
GJC3	5.73×10^{-9}	2.79×10^{-7}	-4.19	7.93×10^{-1}	1.21×10
SLC7A3	3.24×10^{-5}	5.06×10^{-4}	-1.97	7.92×10^{-1}	2.52
AZGP1P1	2.51×10^{-11}	2.05×10^{-9}	-5.28	7.91×10^{-1}	1.41×10
CLSTN2	6.36×10^{-4}	5.21×10^{-3}	-1.19	7.88×10^{-1}	1.74
C5orf38	4.77×10^{-5}	6.79×10^{-4}	-2.52	7.88×10^{-1}	3.56

MCOLN3	3.28×10^{-8}	1.33×10^{-6}	-1.58	7.81×10^{-1}	2.37
FBXL22	2.08×10^{-9}	1.11×10^{-7}	-1.97	7.81×10^{-1}	3.11
VSIG10L	4.36×10^{-13}	5.14×10^{-11}	-3.55	7.74×10^{-1}	9.84
STAC2	7.41×10^{-8}	2.68×10^{-6}	-2.20	7.72×10^{-1}	3.38
SRL	1.21×10^{-5}	2.24×10^{-4}	-1.32	7.71×10^{-1}	1.88
KLHDC8A	3.57×10^{-8}	1.44×10^{-6}	-2.43	7.68×10^{-1}	4.54
DYNC1I1	2.39×10^{-7}	7.58×10^{-6}	-2.21	7.64×10^{-1}	3.48
CCDC15	1.31×10^{-4}	1.51×10^{-3}	-1.32	7.62×10^{-1}	1.86
ANO7	3.35×10^{-7}	1.01×10^{-5}	-1.68	7.56×10^{-1}	2.38
SFTPD	1.47×10^{-5}	2.62×10^{-4}	-1.31	7.54×10^{-1}	1.87
REEP1	4.01×10^{-8}	1.57×10^{-6}	-2.10	7.53×10^{-1}	3.22
ENTPD8	1.25×10^{-5}	2.31×10^{-4}	-1.84	7.50×10^{-1}	2.81
CLUL1	1.84×10^{-3}	1.21×10^{-2}	-1.05	7.49×10^{-1}	1.48
CHRM3	9.40×10^{-8}	3.31×10^{-6}	-2.42	7.44×10^{-1}	4.15
GLRB	7.31×10^{-11}	5.46×10^{-9}	-2.63	7.37×10^{-1}	3.94

ACADL	2.59×10^{-6}	6.15×10^{-5}	-2.62	7.31×10^{-1}	3.58
TRABD2B	3.03×10^{-7}	9.30×10^{-6}	-1.73	7.29×10^{-1}	2.25
CRHBP	1.40×10^{-7}	4.63×10^{-6}	-3.00	7.24×10^{-1}	6.40
CCBE1	8.71×10^{-3}	4.11×10^{-2}	-2.19	7.22×10^{-1}	1.24
MYO7B	1.40×10^{-4}	1.59×10^{-3}	-1.13	7.19×10^{-1}	1.50
HSD17B6	2.27×10^{-15}	4.14×10^{-13}	-2.83	7.15×10^{-1}	4.75
GLIS1	1.38×10^{-9}	7.71×10^{-8}	-1.79	7.14×10^{-1}	2.33
IRX1	3.97×10^{-7}	1.17×10^{-5}	-2.59	7.03×10^{-1}	3.45
CRTAC1	3.42×10^{-4}	3.17×10^{-3}	-1.93	6.98×10^{-1}	2.64
HPSE2	2.93×10^{-5}	4.65×10^{-4}	-2.87	6.97×10^{-1}	4.76
LINC01152	7.20×10^{-4}	5.77×10^{-3}	-1.32	6.81×10^{-1}	1.92
IL17D	8.84×10^{-5}	1.10×10^{-3}	-1.39	6.76×10^{-1}	1.67
LINC00930	1.19×10^{-5}	2.22×10^{-4}	-1.57	6.74×10^{-1}	1.81
GSTO2	7.84×10^{-18}	2.52×10^{-15}	-2.32	6.71×10^{-1}	3.35
ERBB4	1.07×10^{-5}	2.04×10^{-4}	-1.86	6.60×10^{-1}	2.07

MYZAP	5.07×10^{-4}	4.33×10^{-3}	-1.15	6.59×10^{-1}	1.40
MAPK8IP2	5.26×10^{-4}	4.47×10^{-3}	-1.73	6.59×10^{-1}	2.04
ZNF385C	6.15×10^{-4}	5.08×10^{-3}	-1.28	6.58×10^{-1}	1.47
PRIMA1	7.58×10^{-17}	1.85×10^{-14}	-3.17	6.57×10^{-1}	5.39
ASTN2	3.34×10^{-7}	1.01×10^{-5}	-1.39	6.57×10^{-1}	1.79
CCL16	6.52×10^{-3}	3.31×10^{-2}	-1.38	6.51×10^{-1}	1.78
ESRRG	1.72×10^{-4}	1.87×10^{-3}	-1.83	6.51×10^{-1}	2.22
NIPAL1	4.64×10^{-4}	4.03×10^{-3}	-1.28	6.50×10^{-1}	1.73
GRIN1	5.86×10^{-16}	1.21×10^{-13}	-3.87	6.48×10^{-1}	7.34
RGS6	6.98×10^{-3}	3.48×10^{-2}	-1.16	6.47×10^{-1}	1.09
PRKG1	2.55×10^{-3}	1.58×10^{-2}	-1.04	6.46×10^{-1}	1.29
MLLT4-AS1	9.36×10^{-3}	4.34×10^{-2}	-1.06	6.46×10^{-1}	1.23
ID2-AS1	1.69×10^{-3}	1.13×10^{-2}	-1.09	6.46×10^{-1}	1.34
DAB1	1.02×10^{-8}	4.73×10^{-7}	-1.88	6.43×10^{-1}	2.28
LINC01395	3.53×10^{-7}	1.06×10^{-5}	-2.20	6.39×10^{-1}	2.66

CSDC2	1.06×10^{-7}	3.65×10^{-6}	-3.15	6.37×10^{-1}	3.28
LOC644838	1.62×10^{-10}	1.11×10^{-8}	-4.36	6.32×10^{-1}	8.86
GPR37	7.00×10^{-4}	5.63×10^{-3}	-1.51	6.28×10^{-1}	1.80
AGMAT	4.93×10^{-5}	6.98×10^{-4}	-1.19	6.27×10^{-1}	1.36
KRT81	2.42×10^{-11}	1.98×10^{-9}	-2.57	6.26×10^{-1}	3.31
ST7-AS1	3.28×10^{-4}	3.08×10^{-3}	-1.34	6.22×10^{-1}	1.47
CADM3	4.55×10^{-10}	2.81×10^{-8}	-2.80	6.21×10^{-1}	3.48
PCSK2	3.13×10^{-7}	9.58×10^{-6}	-3.16	6.15×10^{-1}	3.41
GATA6-AS1	8.97×10^{-3}	4.21×10^{-2}	-1.19	6.15×10^{-1}	1.19
LONRF3	1.70×10^{-4}	1.85×10^{-3}	-1.63	6.12×10^{-1}	2.19
MBNL1-AS1	8.12×10^{-10}	4.78×10^{-8}	-1.37	6.10×10^{-1}	1.56
C1orf64	1.58×10^{-5}	2.77×10^{-4}	-2.57	6.08×10^{-1}	3.25
ADAMTSL3	5.33×10^{-5}	7.43×10^{-4}	-1.57	6.01×10^{-1}	1.72
RADIL	5.43×10^{-5}	7.51×10^{-4}	-1.01	6.00×10^{-1}	1.17
FIBIN	6.24×10^{-5}	8.35×10^{-4}	-2.43	5.97×10^{-1}	3.12

SLCO4A1-AS1	9.72×10^{-11}	7.02×10^{-9}	-3.28	5.96×10^{-1}	4.33
ZFHX4	1.45×10^{-5}	2.59×10^{-4}	-1.30	5.90×10^{-1}	1.39
ANGPTL1	1.29×10^{-15}	2.50×10^{-13}	-3.95	5.89×10^{-1}	7.78
OCA2	1.60×10^{-5}	2.80×10^{-4}	-1.30	5.86×10^{-1}	1.43
ANK2	1.34×10^{-6}	3.48×10^{-5}	-1.26	5.75×10^{-1}	1.24
HEXA-AS1	2.45×10^{-6}	5.83×10^{-5}	-1.49	5.73×10^{-1}	1.55
IL19	8.57×10^{-10}	5.02×10^{-8}	-1.93	5.68×10^{-1}	2.24
DGKG	2.66×10^{-16}	5.84×10^{-14}	-2.48	5.68×10^{-1}	3.18
JPH2	3.80×10^{-16}	8.24×10^{-14}	-3.34	5.68×10^{-1}	4.57
ACVR2B-AS1	1.07×10^{-2}	4.85×10^{-2}	-1.12	5.62×10^{-1}	1.07
RAB9B	9.82×10^{-8}	3.43×10^{-6}	-1.27	5.53×10^{-1}	1.37
DLX6-AS1	4.97×10^{-4}	4.27×10^{-3}	-1.95	5.48×10^{-1}	1.60
ISM1	3.19×10^{-11}	2.53×10^{-9}	-2.08	5.45×10^{-1}	2.32
PRSS35	1.60×10^{-3}	1.09×10^{-2}	-1.61	5.41×10^{-1}	1.43
DLG2	1.50×10^{-4}	1.67×10^{-3}	-1.37	5.41×10^{-1}	1.29

LINC01554	1.26×10^{-15}	2.46×10^{-13}	-3.13	5.36×10^{-1}	5.51
KLHL35	3.10×10^{-3}	1.84×10^{-2}	-1.03	5.33×10^{-1}	1.14
CORIN	1.25×10^{-4}	1.45×10^{-3}	-3.10	5.32×10^{-1}	1.09×10
FLRT1	1.56×10^{-9}	8.58×10^{-8}	-1.57	5.30×10^{-1}	1.54
LGI2	1.95×10^{-4}	2.07×10^{-3}	-1.42	5.26×10^{-1}	1.23
GPC6	5.80×10^{-4}	4.83×10^{-3}	-1.11	5.25×10^{-1}	1.11
SGCD	6.00×10^{-6}	1.26×10^{-4}	-1.61	5.23×10^{-1}	1.43
NPY5R	5.16×10^{-6}	1.12×10^{-4}	-1.89	5.23×10^{-1}	1.89
ACR	5.46×10^{-3}	2.87×10^{-2}	-1.52	5.23×10^{-1}	1.30
VWDE	5.15×10^{-7}	1.49×10^{-5}	-2.32	5.19×10^{-1}	2.35
SATB2	2.95×10^{-11}	2.35×10^{-9}	-1.57	5.15×10^{-1}	1.58
ITPR1-AS1	1.66×10^{-3}	1.12×10^{-2}	-1.12	5.12×10^{-1}	1.10
AQP7P1	1.69×10^{-28}	1.94×10^{-25}	-3.17	5.10×10^{-1}	4.41
FOXL1	5.59×10^{-10}	3.42×10^{-8}	-1.93	5.09×10^{-1}	1.83
TUSC8	1.20×10^{-5}	2.23×10^{-4}	-3.44	5.08×10^{-1}	3.38

DIRAS1	5.98×10^{-18}	2.00×10^{-15}	-2.96	5.05×10^{-1}	3.88
ALX1	3.60×10^{-7}	1.08×10^{-5}	-3.00	5.02×10^{-1}	4.15
RPRM	2.69×10^{-5}	4.35×10^{-4}	-2.76	4.98×10^{-1}	3.00
PI16	1.44×10^{-7}	4.75×10^{-6}	-3.71	4.97×10^{-1}	4.42
ADGRG2	7.33×10^{-8}	2.66×10^{-6}	-2.95	4.86×10^{-1}	3.56
SYDE2	1.61×10^{-4}	1.77×10^{-3}	-1.12	4.85×10^{-1}	1.05
TNFSF15	3.82×10^{-5}	5.76×10^{-4}	-1.24	4.80×10^{-1}	1.10
AQP2	4.52×10^{-3}	2.46×10^{-2}	-1.57	4.79×10^{-1}	1.00
CNGA1	1.83×10^{-3}	1.21×10^{-2}	-1.73	4.75×10^{-1}	1.86
EEF1A2	2.02×10^{-3}	1.30×10^{-2}	-1.55	4.75×10^{-1}	1.45
CABP1	2.14×10^{-6}	5.20×10^{-5}	-1.52	4.74×10^{-1}	1.30
PLA2G2A	2.83×10^{-8}	1.17×10^{-6}	-6.39	4.68×10^{-1}	2.08×10
SLC1A7	3.77×10^{-4}	3.40×10^{-3}	-1.06	4.63×10^{-1}	9.14×10^{-1}
MYOCD	1.75×10^{-12}	1.79×10^{-10}	-2.64	4.61×10^{-1}	2.49
KRT86	3.93×10^{-8}	1.55×10^{-6}	-1.76	4.59×10^{-1}	1.58

B3GAT1	$5.47×10^{-6}$	$1.17×10^{-4}$	-1.32	$4.59×10^{-1}$	1.15
BVES	$1.85×10^{-8}$	$8.06×10^{-7}$	-1.53	$4.47×10^{-1}$	1.37
TMOD1	$2.60×10^{-18}$	$9.94×10^{-16}$	-2.56	$4.46×10^{-1}$	2.54
ENDOU	$4.20×10^{-3}$	$2.34×10^{-2}$	-1.35	$4.42×10^{-1}$	1.04
C2CD4D	$8.72×10^{-4}$	$6.63×10^{-3}$	-1.41	$4.41×10^{-1}$	1.16
EGF	$3.17×10^{-14}$	$4.43×10^{-12}$	-4.76	$4.41×10^{-1}$	7.26
MYL4	$6.74×10^{-17}$	$1.71×10^{-14}$	-3.39	$4.31×10^{-1}$	4.30
DTNA	$7.60×10^{-17}$	$1.85×10^{-14}$	-2.21	$4.31×10^{-1}$	1.96
PTH2R	$8.03×10^{-6}$	$1.62×10^{-4}$	-1.50	$4.31×10^{-1}$	1.22
LOC101927070	$7.00×10^{-4}$	$5.63×10^{-3}$	-1.20	$4.30×10^{-1}$	1.10
RNF112	$1.63×10^{-18}$	$6.53×10^{-16}$	-2.75	$4.30×10^{-1}$	3.05
LOC102723854	$1.08×10^{-3}$	$7.89×10^{-3}$	-2.23	$4.29×10^{-1}$	1.31
SSPO	$7.89×10^{-5}$	$1.00×10^{-3}$	-1.00	$4.26×10^{-1}$	$8.15×10^{-1}$
KCNK3	$5.23×10^{-14}$	$6.99×10^{-12}$	-3.42	$4.25×10^{-1}$	4.28
FBXW4P1	$2.31×10^{-3}$	$1.45×10^{-2}$	-1.05	$4.13×10^{-1}$	1.00

GRIA2	3.06×10^{-4}	2.91×10^{-3}	-3.41	4.12×10^{-1}	2.10
PLA2G5	1.87×10^{-3}	1.23×10^{-2}	-2.23	4.09×10^{-1}	1.52
EBF2	1.13×10^{-3}	8.20×10^{-3}	-1.32	4.08×10^{-1}	9.44×10^{-1}
GABRG3	1.49×10^{-5}	2.65×10^{-4}	-2.38	4.05×10^{-1}	1.56
KCNH8	3.36×10^{-13}	4.09×10^{-11}	-2.63	3.99×10^{-1}	2.32
OGN	5.74×10^{-9}	2.79×10^{-7}	-4.34	3.98×10^{-1}	3.86
MFAP5	5.82×10^{-6}	1.23×10^{-4}	-3.68	3.93×10^{-1}	2.40
LINC00327	8.14×10^{-15}	1.31×10^{-12}	-2.73	3.89×10^{-1}	2.57
POU5F1B	2.18×10^{-4}	2.26×10^{-3}	-1.62	3.88×10^{-1}	1.13
IRX4	1.23×10^{-4}	1.44×10^{-3}	-2.28	3.80×10^{-1}	1.25
ADORA1	1.11×10^{-4}	1.31×10^{-3}	-1.64	3.74×10^{-1}	1.24
ZFPM2	1.02×10^{-5}	1.97×10^{-4}	-1.66	3.72×10^{-1}	1.20
B4GALNT1	8.05×10^{-11}	5.96×10^{-9}	-2.21	3.70×10^{-1}	1.61
ANKS1B	1.02×10^{-3}	7.52×10^{-3}	-1.10	3.65×10^{-1}	7.65×10^{-1}
PEBP4	5.65×10^{-4}	4.74×10^{-3}	-2.85	3.65×10^{-1}	2.63

CHRM1	3.66×10^{-15}	6.18×10^{-13}	-5.69	3.64×10^{-1}	1.20×10
EPHB1	3.07×10^{-5}	4.84×10^{-4}	-1.81	3.62×10^{-1}	1.33
PGR	3.04×10^{-4}	2.89×10^{-3}	-1.35	3.60×10^{-1}	7.76×10^{-1}
PART1	5.36×10^{-11}	4.11×10^{-9}	-4.36	3.59×10^{-1}	5.49
SYN2	2.88×10^{-4}	2.78×10^{-3}	-1.42	3.57×10^{-1}	8.53×10^{-1}
B3GALT5	9.23×10^{-6}	1.82×10^{-4}	-2.29	3.55×10^{-1}	1.36
LINC01239	5.00×10^{-6}	1.09×10^{-4}	-2.73	3.52×10^{-1}	2.04
LINC01354	1.16×10^{-4}	1.37×10^{-3}	-1.67	3.49×10^{-1}	9.60×10^{-1}
FRMPD3	1.46×10^{-4}	1.64×10^{-3}	-1.51	3.45×10^{-1}	8.65×10^{-1}
CHRNA9	1.01×10^{-3}	7.46×10^{-3}	-1.24	3.40×10^{-1}	8.89×10^{-1}
MPO	3.41×10^{-21}	2.02×10^{-18}	-6.26	3.39×10^{-1}	1.75×10
NOX5	3.53×10^{-4}	3.25×10^{-3}	-2.54	3.39×10^{-1}	1.84
TRIM9	2.24×10^{-17}	6.49×10^{-15}	-2.13	3.32×10^{-1}	1.38
FEZF1-AS1	8.83×10^{-4}	6.70×10^{-3}	-1.91	3.32×10^{-1}	1.40
PLXNA4	1.04×10^{-5}	2.00×10^{-4}	-1.98	3.06×10^{-1}	1.09

VEPH1	1.07×10^{-3}	7.84×10^{-3}	-1.23	3.06×10^{-1}	6.56×10^{-1}
EPHA10	1.56×10^{-10}	1.07×10^{-8}	-2.79	3.06×10^{-1}	1.64
MYBPC1	4.29×10^{-20}	2.15×10^{-17}	-4.82	3.05×10^{-1}	6.87
SLC16A10	8.41×10^{-5}	1.05×10^{-3}	-1.38	3.04×10^{-1}	8.48×10^{-1}
NXPE4	1.01×10^{-6}	2.70×10^{-5}	-1.63	3.03×10^{-1}	8.37×10^{-1}
CYP3A5	3.06×10^{-4}	2.91×10^{-3}	-2.64	3.01×10^{-1}	1.78
C10orf82	2.92×10^{-12}	2.83×10^{-10}	-2.43	3.01×10^{-1}	1.50
MATN4	1.29×10^{-9}	7.28×10^{-8}	-3.51	3.00×10^{-1}	3.05
GREB1	6.27×10^{-5}	8.38×10^{-4}	-1.35	2.98×10^{-1}	7.49×10^{-1}
OTUD7A	2.21×10^{-4}	2.28×10^{-3}	-1.21	2.94×10^{-1}	6.68×10^{-1}
CPLX3	8.56×10^{-8}	3.04×10^{-6}	-4.03	2.93×10^{-1}	5.81
GALNT13	2.87×10^{-11}	2.30×10^{-9}	-4.36	2.90×10^{-1}	3.26
RNF182	3.43×10^{-4}	3.18×10^{-3}	-1.35	2.89×10^{-1}	7.88×10^{-1}
PLA2G3	2.69×10^{-7}	8.37×10^{-6}	-1.76	2.87×10^{-1}	1.06
PPP1R1A	1.23×10^{-4}	1.43×10^{-3}	-2.61	2.86×10^{-1}	1.40

SCN3B	3.11×10^{-4}	2.95×10^{-3}	-1.29	2.86×10^{-1}	7.17×10^{-1}
HMCN2	4.06×10^{-11}	3.16×10^{-9}	-2.05	2.85×10^{-1}	1.19
SLC27A6	1.90×10^{-6}	4.71×10^{-5}	-2.58	2.78×10^{-1}	1.48
MROH7	5.90×10^{-4}	4.91×10^{-3}	-1.07	2.76×10^{-1}	6.01×10^{-1}
TRIM58	6.11×10^{-3}	3.15×10^{-2}	-1.07	2.69×10^{-1}	5.19×10^{-1}
UNC5A	4.99×10^{-3}	2.67×10^{-2}	-1.36	2.69×10^{-1}	6.41×10^{-1}
KY	3.27×10^{-3}	1.92×10^{-2}	-1.20	2.68×10^{-1}	6.56×10^{-1}
ZNF536	3.30×10^{-3}	1.94×10^{-2}	-1.35	2.67×10^{-1}	5.23×10^{-1}
KLHL30	3.11×10^{-8}	1.27×10^{-6}	-2.11	2.56×10^{-1}	1.15
SEMA3E	4.96×10^{-7}	1.44×10^{-5}	-1.93	2.52×10^{-1}	9.38×10^{-1}
NPAS4	1.66×10^{-5}	2.88×10^{-4}	-3.50	2.49×10^{-1}	2.22
GP2	7.07×10^{-8}	2.58×10^{-6}	-10.3	2.48×10^{-1}	2.10×10
SOX9-AS1	1.03×10^{-4}	1.24×10^{-3}	-1.47	2.47×10^{-1}	6.34×10^{-1}
PRKG2	5.58×10^{-7}	1.59×10^{-5}	-1.87	2.43×10^{-1}	7.88×10^{-1}
RYR2	7.83×10^{-6}	1.58×10^{-4}	-1.91	2.42×10^{-1}	9.49×10^{-1}

CCSER1	7.55×10^{-4}	5.99×10^{-3}	-1.04	2.42×10^{-1}	5.42×10^{-1}
SLITRK5	2.14×10^{-12}	2.16×10^{-10}	-4.36	2.42×10^{-1}	3.37
TMEM145	5.33×10^{-8}	1.99×10^{-6}	-2.12	2.42×10^{-1}	1.02
CHMP1B2P	8.82×10^{-3}	4.15×10^{-2}	-1.59	2.41×10^{-1}	5.84×10^{-1}
GLP1R	1.18×10^{-3}	8.45×10^{-3}	-1.26	2.39×10^{-1}	5.58×10^{-1}
KCNS1	9.45×10^{-12}	8.33×10^{-10}	-2.94	2.36×10^{-1}	2.19
RPLP0P2	1.50×10^{-3}	1.04×10^{-2}	-1.12	2.34×10^{-1}	4.64×10^{-1}
FAM150B	1.25×10^{-21}	8.03×10^{-19}	-3.57	2.34×10^{-1}	2.65
PSD2	3.87×10^{-5}	5.80×10^{-4}	-1.44	2.32×10^{-1}	6.45×10^{-1}
GRIK1	9.70×10^{-3}	4.48×10^{-2}	-1.79	2.31×10^{-1}	1.73
TGM5	1.01×10^{-5}	1.96×10^{-4}	-2.27	2.30×10^{-1}	9.07×10^{-1}
LOC101927583	5.57×10^{-4}	4.68×10^{-3}	-1.71	2.19×10^{-1}	7.01×10^{-1}
RNASE7	4.11×10^{-29}	5.99×10^{-26}	-3.55	2.17×10^{-1}	2.62
CASKIN1	8.43×10^{-8}	3.01×10^{-6}	-2.21	2.15×10^{-1}	8.44×10^{-1}
EDN3	2.69×10^{-5}	4.35×10^{-4}	-3.26	2.13×10^{-1}	1.60

KCNK2	3.46×10^{-5}	5.31×10^{-4}	-2.94	2.13×10^{-1}	9.84×10^{-1}
C20orf166-AS1	2.67×10^{-14}	3.82×10^{-12}	-3.00	2.11×10^{-1}	1.71
ASXL3	8.50×10^{-4}	6.51×10^{-3}	-1.18	2.06×10^{-1}	4.29×10^{-1}
TNNI1	5.94×10^{-4}	4.93×10^{-3}	-1.01	2.04×10^{-1}	4.12×10^{-1}
ACAN	2.65×10^{-3}	1.62×10^{-2}	-1.36	2.04×10^{-1}	5.10×10^{-1}
NTNG1	7.56×10^{-3}	3.70×10^{-2}	-1.57	2.03×10^{-1}	6.61×10^{-1}
FBN3	1.35×10^{-13}	1.70×10^{-11}	-2.66	2.03×10^{-1}	1.17
MAPT	5.46×10^{-6}	1.17×10^{-4}	-2.33	2.02×10^{-1}	9.53×10^{-1}
FAM196B	1.18×10^{-3}	8.45×10^{-3}	-1.35	2.02×10^{-1}	6.01×10^{-1}
FOXD2	8.88×10^{-3}	4.17×10^{-2}	-1.27	2.02×10^{-1}	5.03×10^{-1}
SLC6A17	5.13×10^{-14}	6.92×10^{-12}	-3.14	2.01×10^{-1}	1.64
PRR4	1.51×10^{-36}	4.03×10^{-33}	-5.14	2.00×10^{-1}	7.17
DLGAP1-AS5	8.72×10^{-6}	1.74×10^{-4}	-6.07	1.98×10^{-1}	3.67
PKHD1L1	1.72×10^{-3}	1.15×10^{-2}	-1.41	1.98×10^{-1}	4.55×10^{-1}
DLGAP1	3.29×10^{-3}	1.93×10^{-2}	-1.25	1.97×10^{-1}	4.07×10^{-1}

CLDN10-AS1	1.41×10^{-8}	6.37×10^{-7}	-4.10	1.94×10^{-1}	2.74
GDF5	9.81×10^{-8}	3.43×10^{-6}	-2.42	1.93×10^{-1}	1.11
PDZRN3-AS1	2.60×10^{-5}	4.24×10^{-4}	-1.66	1.93×10^{-1}	5.67×10^{-1}
FGF12	2.69×10^{-14}	3.82×10^{-12}	-3.16	1.92×10^{-1}	1.74
CGREF1	1.06×10^{-20}	5.89×10^{-18}	-4.82	1.88×10^{-1}	4.91
LMAN1L	9.36×10^{-14}	1.21×10^{-11}	-4.75	1.88×10^{-1}	6.01
NIM1K	2.44×10^{-5}	4.03×10^{-4}	-1.55	1.86×10^{-1}	5.19×10^{-1}
ANO5	5.10×10^{-10}	3.14×10^{-8}	-2.48	1.86×10^{-1}	9.39×10^{-1}
LOC101929563	2.11×10^{-3}	1.35×10^{-2}	-1.06	1.85×10^{-1}	3.95×10^{-1}
GFAP	1.76×10^{-3}	1.17×10^{-2}	-2.31	1.85×10^{-1}	6.48×10^{-1}
ROPN1	2.72×10^{-11}	2.19×10^{-9}	-3.41	1.84×10^{-1}	1.88
LINGO4	5.99×10^{-8}	2.21×10^{-6}	-2.45	1.84×10^{-1}	9.47×10^{-1}
KCNT1	8.05×10^{-3}	3.86×10^{-2}	-1.27	1.82×10^{-1}	4.67×10^{-1}
NLGN4X	5.09×10^{-9}	2.52×10^{-7}	-2.29	1.81×10^{-1}	8.01×10^{-1}
IGSF11	1.32×10^{-10}	9.28×10^{-9}	-2.20	1.78×10^{-1}	7.98×10^{-1}

ABCC8	8.66×10^{-3}	4.09×10^{-2}	-2.18	1.77×10^{-1}	9.18×10^{-1}
LAMC3	1.72×10^{-3}	1.15×10^{-2}	-1.18	1.76×10^{-1}	3.95×10^{-1}
GPR63	3.34×10^{-5}	5.18×10^{-4}	-1.06	1.75×10^{-1}	3.69×10^{-1}
LOC100506497	1.14×10^{-5}	2.14×10^{-4}	-1.77	1.74×10^{-1}	6.06×10^{-1}
ASIC4	5.58×10^{-12}	5.06×10^{-10}	-2.48	1.74×10^{-1}	1.07
ESYT3	3.95×10^{-3}	2.22×10^{-2}	-1.30	1.73×10^{-1}	4.35×10^{-1}
C14orf180	3.85×10^{-8}	1.53×10^{-6}	-4.32	1.72×10^{-1}	1.98
TMEM255A	6.36×10^{-8}	2.33×10^{-6}	-2.51	1.72×10^{-1}	8.67×10^{-1}
PHYHIPL	1.47×10^{-3}	1.02×10^{-2}	-1.76	1.70×10^{-1}	5.53×10^{-1}
LMO3	4.83×10^{-8}	1.84×10^{-6}	-3.48	1.62×10^{-1}	1.22
HFM1	3.36×10^{-5}	5.20×10^{-4}	-1.36	1.62×10^{-1}	3.95×10^{-1}
TRHDE-AS1	6.57×10^{-3}	3.32×10^{-2}	-1.14	1.62×10^{-1}	2.87×10^{-1}
ADRA1A	2.42×10^{-10}	1.59×10^{-8}	-4.36	1.61×10^{-1}	2.59
EPHA7	5.63×10^{-4}	4.72×10^{-3}	-2.27	1.56×10^{-1}	6.51×10^{-1}
PLCXD3	5.01×10^{-9}	2.49×10^{-7}	-2.93	1.45×10^{-1}	9.43×10^{-1}

PLG	1.16×10^{-12}	1.25×10^{-10}	-2.60	1.38×10^{-1}	8.13×10^{-1}
ADCYAP1R1	2.93×10^{-4}	2.81×10^{-3}	-3.29	1.35×10^{-1}	5.27×10^{-1}
INSC	1.63×10^{-20}	8.71×10^{-18}	-2.90	1.34×10^{-1}	9.82×10^{-1}
RELN	2.13×10^{-8}	9.16×10^{-7}	-2.52	1.34×10^{-1}	6.66×10^{-1}
SCN4A	5.51×10^{-6}	1.17×10^{-4}	-1.65	1.32×10^{-1}	4.00×10^{-1}
SLC35F1	6.24×10^{-12}	5.59×10^{-10}	-2.83	1.32×10^{-1}	8.33×10^{-1}
TRHDE	4.49×10^{-3}	2.45×10^{-2}	-1.19	1.26×10^{-1}	2.34×10^{-1}
GRIN2A	6.95×10^{-5}	9.09×10^{-4}	-2.29	1.23×10^{-1}	5.03×10^{-1}
SLC1A2	8.26×10^{-23}	6.31×10^{-20}	-2.97	1.22×10^{-1}	9.91×10^{-1}
FAM189A1	4.62×10^{-12}	4.31×10^{-10}	-2.55	1.20×10^{-1}	6.70×10^{-1}
CECR2	2.81×10^{-3}	1.71×10^{-2}	-1.45	1.17×10^{-1}	2.60×10^{-1}
GLYATL1	1.07×10^{-15}	2.15×10^{-13}	-3.46	1.16×10^{-1}	1.26
ACVR1C	1.62×10^{-7}	5.31×10^{-6}	-1.70	1.08×10^{-1}	3.72×10^{-1}
TDRD5	1.40×10^{-8}	6.33×10^{-7}	-2.78	1.08×10^{-1}	6.34×10^{-1}
DRP2	7.89×10^{-5}	1.00×10^{-3}	-1.78	1.06×10^{-1}	4.36×10^{-1}

TENM1	6.07×10^{-8}	2.23×10^{-6}	-1.63	1.05×10^{-1}	3.03×10^{-1}
SCN7A	8.06×10^{-23}	6.31×10^{-20}	-4.51	1.04×10^{-1}	2.17
LRAT	4.19×10^{-6}	9.34×10^{-5}	-2.27	1.03×10^{-1}	4.29×10^{-1}
COL19A1	4.83×10^{-3}	2.60×10^{-2}	-1.11	9.99×10^{-2}	1.93×10^{-1}
SEMG2	3.35×10^{-4}	3.12×10^{-3}	-6.99	9.59×10^{-2}	4.51
GRIK3	1.91×10^{-7}	6.15×10^{-6}	-4.92	8.52×10^{-2}	1.65
RNASE13	8.56×10^{-13}	9.48×10^{-11}	-4.76	8.51×10^{-2}	2.40
OPCML	1.65×10^{-5}	2.88×10^{-4}	-4.32	8.25×10^{-2}	9.98×10^{-1}
PDZRN4	1.79×10^{-17}	5.32×10^{-15}	-3.59	7.74×10^{-2}	9.26×10^{-1}
SORCS1	2.24×10^{-6}	5.39×10^{-5}	-4.48	7.50×10^{-2}	7.51×10^{-1}
SYT13	1.35×10^{-7}	4.51×10^{-6}	-3.98	7.18×10^{-2}	1.08
ADAMTS19	2.15×10^{-6}	5.20×10^{-5}	-2.61	6.84×10^{-2}	3.29×10^{-1}
CDH19	1.22×10^{-17}	3.69×10^{-15}	-5.98	6.81×10^{-2}	3.31
CACNA1E	5.39×10^{-16}	1.12×10^{-13}	-3.10	6.54×10^{-2}	5.05×10^{-1}
PMP2	4.57×10^{-6}	1.01×10^{-4}	-5.33	6.50×10^{-2}	1.07

NRG3	$4.71×10^{-15}$	$7.88×10^{-13}$	-7.00	$6.13×10^{-2}$	5.52
ANGPTL7	$1.99×10^{-10}$	$1.34×10^{-8}$	-6.38	$5.56×10^{-2}$	2.10
RASGEF1C	$6.09×10^{-25}$	$5.43×10^{-22}$	-4.48	$5.20×10^{-2}$	1.20
RXRG	$8.94×10^{-29}$	$1.10×10^{-25}$	-4.30	$5.13×10^{-2}$	1.03
UNC80	$1.06×10^{-5}$	$2.03×10^{-4}$	-2.79	$4.97×10^{-2}$	$2.45×10^{-1}$
IGSF1	$1.24×10^{-12}$	$1.31×10^{-10}$	-3.81	$4.87×10^{-2}$	$6.93×10^{-1}$
ART3	$2.28×10^{-30}$	$3.66×10^{-27}$	-5.67	$4.69×10^{-2}$	2.89
CNTN2	$4.52×10^{-12}$	$4.24×10^{-10}$	-5.04	$4.36×10^{-2}$	1.07
C10orf90	$4.13×10^{-14}$	$5.66×10^{-12}$	-5.43	$4.19×10^{-2}$	1.96
CELF5	$7.66×10^{-13}$	$8.60×10^{-11}$	-3.68	$3.71×10^{-2}$	$4.28×10^{-1}$
IGFN1	$5.58×10^{-21}$	$3.20×10^{-18}$	-5.77	$3.10×10^{-2}$	1.38
NRXN1	$4.91×10^{-29}$	$6.57×10^{-26}$	-4.49	$2.92×10^{-2}$	$6.34×10^{-1}$
FREM3	$4.01×10^{-17}$	$1.11×10^{-14}$	-6.03	$2.31×10^{-2}$	1.46
RHAG	$2.77×10^{-17}$	$7.80×10^{-15}$	-6.98	$2.20×10^{-2}$	2.00
SCGN	$3.53×10^{-15}$	$6.02×10^{-13}$	-6.24	$1.90×10^{-2}$	1.28

CADM2	$2.12×10^{-10}$	$1.41×10^{-8}$	-4.14	$1.87×10^{-2}$	$2.53×10^{-1}$
HS6ST3	$5.22×10^{-12}$	$4.81×10^{-10}$	-4.83	$1.64×10^{-2}$	$2.99×10^{-1}$
ACTC1	$7.33×10^{-51}$	$5.88×10^{-47}$	-8.89	$9.67×10^{-3}$	4.29

Supplementary Table S3

Periostin deposition pattern in RNA-seq group (n=6).

	Diagnosis	Deposition type
Patient 1	Non-ECRS	Superficial type
Patient 2	Non-ECRS	Superficial type
Patient 3	Moderate ECRS	Diffuse type
Patient 4	Moderate ECRS	Diffuse type
Patient 5	Moderate ECRS	Diffuse type
Patient 6	Severe ECRS	Diffuse type

ECRS, eosinophilic chronic rhinosinusitis
